# *N*-Boc-α-diazo glutarimide as efficient reagent for assembling N-heterocycle-glutarimide diads via Rh(II)-catalyzed N–H insertion reaction

**DOI:** 10.3762/bjoc.19.136

**Published:** 2023-12-07

**Authors:** Grigory Kantin, Pavel Golubev, Alexander Sapegin, Alexander Bunev, Dmitry Dar’in

**Affiliations:** 1 Saint Petersburg State University, Saint Petersburg, 199034, Russian Federation; 2 Medicinal Chemistry Center, Togliatti State University, Togliatti, 445020, Russian Federation; 3 Saint Petersburg Research Institute of Phthisiopulmonology, Saint Petersburg, 191036, Russian Federation; 4 Department of Medicinal Chemistry, Institute of Chemistry, Saint Petersburg State University, 26 Universitetskiy prospekt, Peterhof 198504, Russian Federation

**Keywords:** CRBN ligands, diazocarbonyl compounds, N–H insertion reaction, N-heterocycles, Rh(II)-catalysis

## Abstract

A technique has been proposed for incorporating a heterocyclic component into a glutarimide framework employing a Rh_2_(esp)_2_-catalyzed N–H insertion with the involvement of *N*-Boc-α-diazo glutarimide. The new diazo reagent is more stable, soluble and convenient to prepare than the previously suggested one. The approach permits the application of diverse heterocycles, including both aromatic and saturated NH-substrates. This yields structures that are appealing for generating cereblon ubiquitin-ligase ligands and for potential use in crafting PROTAC molecules.

## Introduction

Targeted protein degradation (TPD) has transformed the field of drug discovery [1–2]. Utilizing proximity-induced pharmacological strategies [3], this method has fostered the creation of numerous molecular glues and proteolysis-targeting chimeras (PROTACs). By manipulation of the internal ubiquitin-proteasome degradation system, it has been achieved to approach previously considered undruggable targets and conceive remedies for drug-resistant targets [4]. The given TPD's benefit over traditional inhibition; numerous proteins have been set for proteasomal degradation by transmuting accessible small molecule inhibitors into PROTACs. The RAS proteins, once seen as drug-resistant, became susceptible and a series of covalent inhibitors [5–7] were synthesized to bind to KRAS^G12C^. The application of the PROTAC principle has demonstrated the feasibility of endogenous degradation of KRAS [8–10], potentially opening a path for its use in treating KRAS-induced cancers.

**Figure 1:**
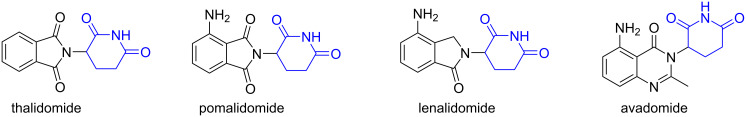
Glutarimide-based immunomodulatory drugs (IMiDs) and CRBN ligands.

A common characteristic of the degraders elaborated in the literature involves the compulsory integration of an E3-ligase ligand motif into the PROTAC configuration [11]. The E3-ligase most frequently utilized in TPD strategies is cereblon (CRBN), the target focus of a collection of immunomodulatory drugs containing the glutarimide moiety such as thalidomide, pomalidomide, lenalidomide [12–13], and avadomide [14] (Figure 1). These ligands, although prevalent recruiters in PROTAC design, present several drawbacks including the degradation of lymphoid transcription factors [15–17] IKZF1, IKZF3, and SALL4 where the latter's degradation could result in a significant teratogenic effect [18]. In addition, these glutarimide derivatives are highly susceptible to hydrolysis and enzymatic cleavage under physiological circumstances, considerably impacting their pharmacological utility [19–20], Moreover, the conventional CRBN recruiters restrict structural modification options necessary to maintain satisfactory affinity for the E3-ligase [21–24]. These limitations underscore the relevance of expanding the chemical space of cereblon ligands.

**Scheme 1:**
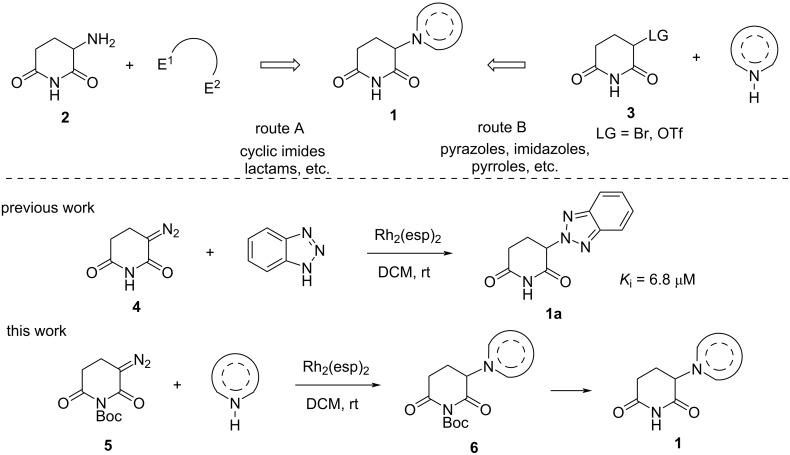
Main literature approaches towards α-hetaryl glutarimides **1** (routes A and B) and new “diazo” methodology based on Rh(II)-catalyzed N–H-insertion reaction.

Research teams and pharmaceutical companies worldwide are actively conducting studies to discover new CRBN ligands based on α-hetaryl-substituted glutarimides of general formula **1** (Scheme 1). Consequently, a significant number of patents describing the synthesis of new PROTAC molecules and CRBN ligands are being published (over 400K patents in the last 5 years according to SciFinder). Various nitrogen heterocycles were utilized as a heterocyclic moiety linked to a glutarimide core via a nitrogen atom. In addition to the phthalimide fragment, the most commonly studied ones are pyrazole derivatives (including indazole), benzimidazole, 1,2,3-triazole, indole, carbazole, indoline, quinazoline, and isoquinoline. Nevertheless, many heterocyclic motifs still remain beyond the attention of researchers. For example, glutarimides that incorporate tetrazole and 1,2,4-triazole substituents at the α-position have not yet been studied.

Typically, two primary strategies are employed to build an N-heterocycle-glutarimide diad. The first involves assembling a heterocyclic fragment utilizing glutamic acid imide (**2**, Scheme 1, route A), while the second involves alkylating NH-heterocycle with α-bromo (α-oxysulfonyl) glutarimide (**3**, Scheme 1, route B). Although the first approach has readily available starting reagents, a simple synthesis process, and gives generally high yields, it is restricted in terms of the type of heterocycle that can be assembled (primarily cyclic imides and lactams). The second approach, which asserts its universality, frequently displays inadequate yield of target compounds. As such, the endeavour to discover novel approaches for synthesizing *N*-heterocycle-glutarimide ensembles of type **1** persists as imperative.

Aliphatic diazocarbonyl reagents are widely acknowledged to be effective in introducing substituents to the nitrogen atoms of NH-heterocycles by means of carbene insertion into the N–H bond upon catalytic or photolytic decomposition of diazo compounds [25]. Furthermore, the reaction of N-heterocycles containing multiple non-equivalent nitrogen atoms with a diazo reagent under neutral conditions may give rise to a separate regioisomer which is different from the product obtained by reaction with an alkyl halide under basic conditions.

Recently, we designed a thalidomide analogue **1a** in which the phthalimide moiety was replaced with benzotriazole, using a new synthesis strategy based on the usage of diazo glutarimide **4** (Scheme 1). Compared to thalidomide (Figure 1), the resulting “benzotriazolo thalidomide” has a similar binding mode, but improved properties, as revealed in crystallographic analyses, affinity assays and cell culture [26]. However, the proposed diazo reagent **4** possesses several drawbacks, primarily, insufficient stability during storage and comparatively low solubility in non-polar solvents, as well as complications when isolating it in a pure form.

This study focuses on the development of a "diazo" technique to incorporate a heterocyclic fragment into the glutarimide core, creating potential CRBN ligands and crucial building blocks for assembling PROTACs. In this paper, a more convenient diazo reagent **5** is introduced and its effectiveness in reacting with a broad variety of NH-heterocycles and in providing *N*-Boc-protected precursors **6** of hetaryl-substituted glutarimides **1** is demonstrated.

## Results and Discussion

**Scheme 2:**
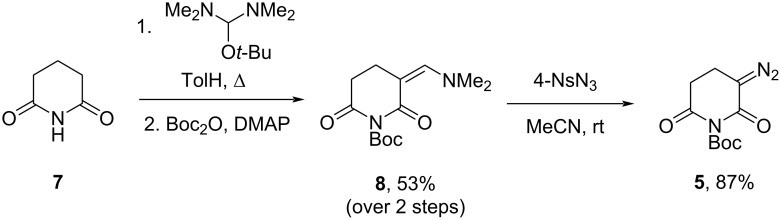
Preparation of diazo reagent **5**.

To address the challenges of working with *N*-unsubstituted diazo glutarimide **4**, a protective and easily removable group was introduced to the molecule. The key diazo reagent **5** was synthesized using modified standard procedures detailed below (Scheme 2). Glutarimide (**7**), which is commercially available, was (dimethylamino)methylenated at the α-position with the Bredereck's reagent before adding the Boc group to the imide nitrogen atom of the crude enamine. The interim derivative **8** was obtained with a yield of 53% over two steps and readily entered the diazo transfer reaction with 4-nitrophenylsulfonyl azide (4-NsN_3_). The resulting diazo reagent **5** was produced in a high yield after undergoing straightforward chromatographic purification.

The use of a Boc group at the nitrogen atom of the diazo imide significantly simplified the process of isolating compound **5**, as compared to the prior publication [26], and notably increased its stability. Furthermore, the solubility of the new diazo reagent in non-polar solvents, particularly DCM, was significantly improved, leading to a beneficial impact on the course of Rh(II)-catalyzed N–H insertion reactions involving NH-heterocycles. The proposed method enables the synthesis of multigram quantities of diazo compound **5** rapidly. Furthermore, it can be stored up to several weeks in the refrigerator (5 °C) without any observable alterations.

**Scheme 3:**
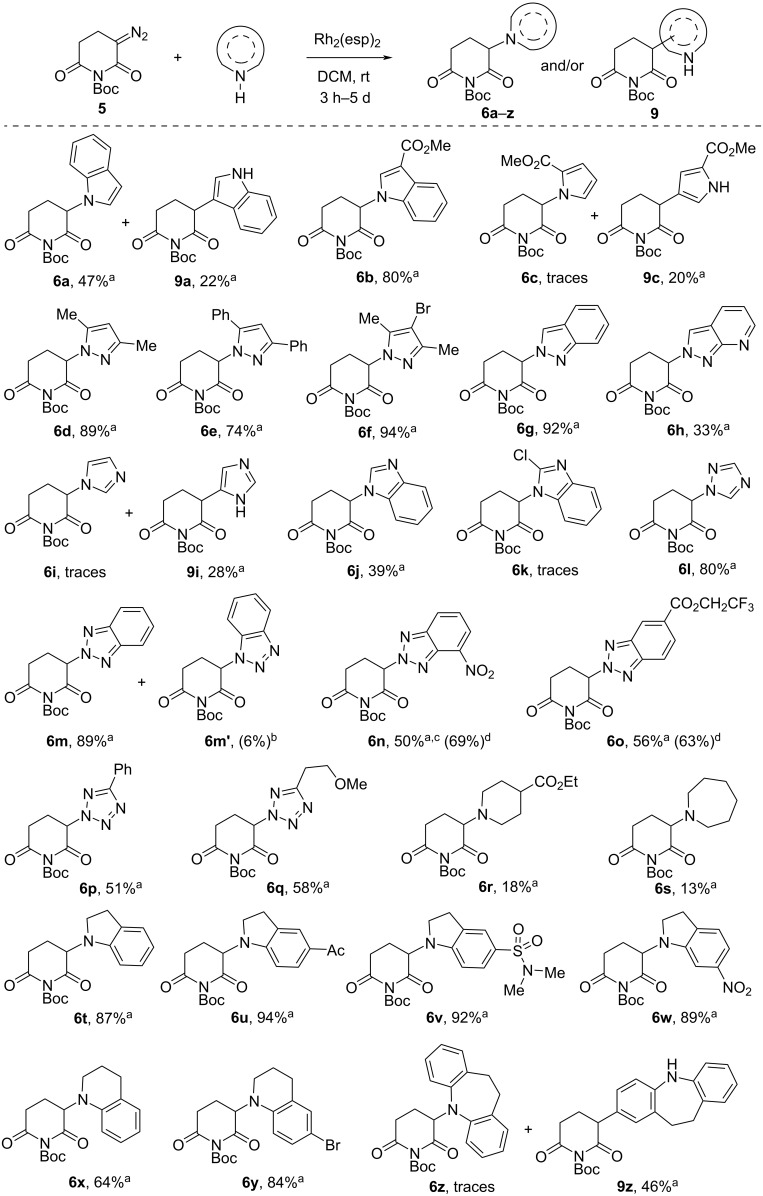
Scope of NH insertion reaction of *N*-Boc-α-diazo glutarimide and various *N*-heterocycles. ^a^Isolated yield; reaction scale ‒ 0.33 mmol of NH-heterocycle, 0.4 mmol of diazo reagent **5**, catalyst ‒ 2.5 mM Rh_2_(esp)_2_ in DCM, 100‒300 μL (0.06‒0.18 mol %); ^b^NMR yield; ^c^structure confirmed by single-crystal X-ray data; ^d^calculated yield based on incomplete conversion of NH-substrate.

A diverse array of NH-heterocycles with varying characteristics were selected as substrates for the studied insertion reaction, encompassing both aromatic and non-aromatic compounds differing in the number and arrangement of nitrogen atoms (Scheme 3). Notably, several of these heterocycles, including tetrahydroquinoline, 1,2,4-triazole, and tetrazoles, had not been previously utilized in the CRBN ligands design. Catalytic decomposition reactions of diazo compound **5** with NH-heterocycles were conducted in a dry DCM solution using dirhodium espinoate (Rh_2_(esp)_2_, 0.06–0.18 mol %). The Rh_2_(esp)_2_ catalyst was selected for its excellent versatility and efficiency in various XH insertion reactions [27–29].

In many cases (e.g., indoles, indazoles, benzotriazoles, tetrazoles, Scheme 3) the reaction progressed expeditiously (as indicated by gas evolution upon adding diazo reagent to the mixture of NH-substrate and catalyst) and was completed in 3–5 h (TLC control). Meanwhile, the disappearance of the diazo reagent was considerably slower in the case of more basic heterocycles (pyrazoles, indolines, tetrahydroquinolines), taking anywhere from 16 to 24 hours, and in some cases (imidazole, 7-azaindazole, ethyl isonipecotate, hexamethylenimine), up to 2–3 days, along with the addition of an extra portion or two of catalyst to complete the reaction. Furthermore, the yields of the NH-insertion products in the latter reactions were moderate or low (see **6h**, **6r**, **6s**, Scheme 3). It should be noted that we have also tested some Cu(II) catalysts (Cu(OTf)_2_ and Cu(acac)_2_ in DCE at 80 °C) in reaction with ethyl isonipecotate (see Supporting Information File 1 for details). While in the case of Cu(OTf)_2_ (5 mol %), the reaction mixture did not contain the desired product **6r**, using Cu(acac)_2_ (10 mol %) resulted in 25% NMR yield of the insertion product. However, this does not show much advantage over Rh_2_(esp)_2_ (0.18 mol %), which gave a preparative yield of 18%. It is important to note that despite the higher cost, the rhodium catalyst offers milder reaction conditions.

In the case of some electron-rich substrates, in addition to N–H insertion products **6**, C–H insertion products **9** were also observed. Thus, when reacting with indole, the product of carbenoid attack at position 3 (**9a**) was isolated along with target compound **6a**. Introduction of a carbomethoxy group into this position of indole leads to the exceptional formation of the N–H insertion product **6b** in high yield. The reaction with methyl pyrrole-2-carboxylate resulted in the isolation of only the C–H insertion product **9c** in low yield. Similar reaction progress was observed in the case with imidazole, the product N–H insertion was observed only in trace amounts (according to NMR data of the reaction mixture). The structure of the main reaction product **9i** was confirmed by 2D HSQC NMR spectroscopy.

To evaluate the influence of the catalyst on chemoselectivity of the reaction with indole (ratio **6a**/**9a**) we have performed additional testing with Rh_2_(TFA)_4_ and Rh_2_(OAc)_4_, which differ from the Rh_2_(esp)_2_ in both electronic and steric factors (see Supporting Information File 1). When Rh_2_(OAc)_4_ (1 mol %) was used, a 1.7:1 ratio of **6a**/**9a** insertion products was observed, which is not much different from the result obtained with Rh_2_(esp)_2_. However, the overall yield of insertion products decreased (62% (NMR) vs 69% (isolated)). When Rh_2_(TFA)_4_ (1 mol %) was used, a reversal of the ratio of **6a**/**9a** insertion products (1:2) was observed in the test reaction, although the total yield estimated by NMR was only 32%. Thus, in the series of tested Rh(II) catalysts Rh_2_(esp)_2_ is the most successful catalyst for obtaining the product of insertion into the NH bond of the heterocycle.

An effort to obtain the N–H insertion product with dibenzoazepine proved fruitless. Instead, the product of insertion into the C–H bond of the activated benzene ring (**9z**) was isolated in moderate yield. The chemo- and regioselectivity of this reaction can be attributed to several factors, including steric hindrances to the attack of the nitrogen atom by the carbenoid. When reacting with tetrahydroquinoline (with insignificant steric shielding of the *N*-nucleophilic center), the product of N–H insertion **6x** was obtained in good yield. As a side process, а carbenoid's repeated attack at the 6-position of the tetrahydroquinoline ring of compound **6x** was observed according to NMR data. Introducing a bromine atom to block the 6-position led to a significant increase in the target product **6y** yield.

The decomposition of diazo reagent **5** in the presence of symmetrically substituted pyrazoles produced the relevant products of N–H insertion **6d–f** in high yields. The substitution of methyl groups with phenyl groups (**6d** vs **6e**) had no significant impact on the outcome of the reaction. The synthesis of compound **6f** was conducted on a gram scale, and enhancing the scale of the reaction did not affect the product yield (93% vs 94%).

Benzimidazole displayed relatively low reactivity, resulting in moderate yield of the desired product **6j**. The introduction of a chlorine atom at the 2-position of the substrate (2-chlorobenzimidazole) actively suppressed the targeted reaction. Merely traces of the N–H insertion product were detectable in NMR data, with insignificant conversion of the initial heterocycle.

Examples of N–H insertion reactions with azoles containing non-equivalent nitrogen atoms deserve separate discussion. As the only product of the reaction with indazole, compound **6g**, formed as a result of the attack of the carbenoid on the 2-N atom, was obtained. The regioselectivity of the reaction with 7-azaindazole was similar, however, the introduction of an additional basic nitrogen atom resulted in a significant increase in reaction time and a decrease in the yield of the target compound **6h**. The structures of the obtained regioisomers **6g** and **6h** were confirmed by the NOESY spectra, in which cross-peaks between the protons of the glutarimide ring and the singlet of the proton of the pyrazole cycle are observed.

Reactions with benzotriazoles are also highly regioselective, proceeding via attack on the 2-N atom to give products **6m–o**. In the instance of unsubstituted benzotriazole, a minor regioisomer **6m'** was identified with a calculated yield of 6% using NMR data. Benzotriazoles featuring electron-withdrawing substituents showed incomplete conversion of the NH-heterocycle, and the yields of target compounds **6n** and **6o** were slightly diminished. The single-crystal X-ray data verified the structure of product **6n**. The data obtained on the regioselectivity of reactions with benzotriazoles are in agreement with those previously reported in the literature [30].

Despite the low reactivity (the reaction was carried out for 5 days with triple portion of the catalyst), the yield of the N–H insertion product involving 1,2,4-triazole was unexpectedly high (80%). Compound **6l** was obtained as a single regioisomer. The reaction with tetrazoles is also characterized by high regioselectivity. We obtained tetrazol-2-yl glutarimides **6p and 6q** in moderate yields. Alternative regioisomers (tetrazol-1-yl glutarimides) were observed only in trace amounts (according to NMR data). It should be noted that in this work the insertion of the diazocarbonyl reagent into the N–H bond of tetrazoles and 1,2,4-triazole was realized for the first time. This transformation can serve as a powerful tool to carry out *N*-modification of these heterocycles.

**Figure 2:**
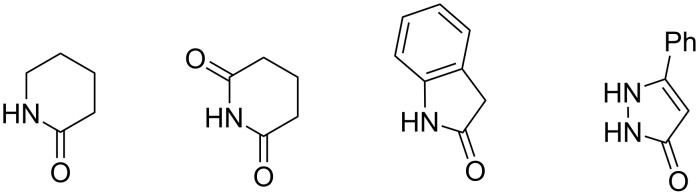
Examples of α-carbonyl NH-heterocycles for which N–H insertion products could not be obtained.

Despite all our efforts, it was not possible to obtain N–H insertion products with *N*-heterocycles containing an α-carbonyl group (Figure 2). We observed formation of complex mixtures in the case of δ-valerolactam, glutarimide and oxindole. This observation can be attributed to the competing direction of the attack of the carbenoid onto the carbonyl oxygen atom resulting in unstable intermediates. In the reaction with 5-phenylpyrazolin-3-one, an exceptionally low conversion of the starting heterocycle was observed.

**Scheme 4:**
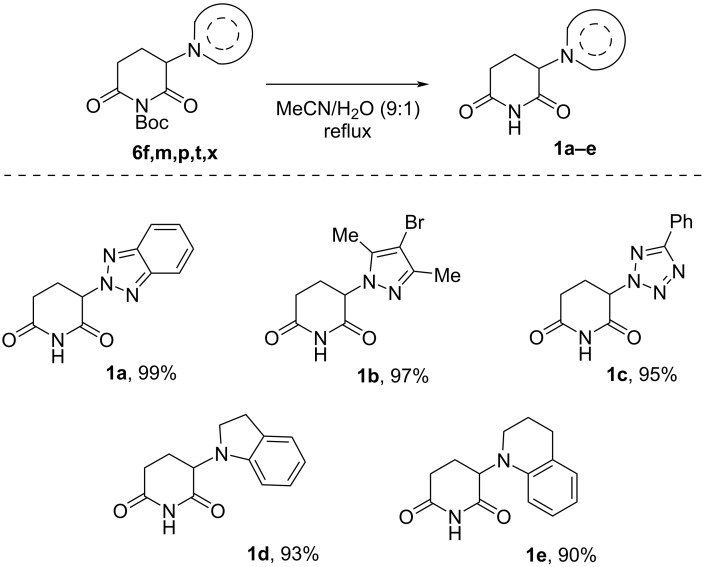
Examples of *N*-deprotection of α-modified glutarimides **1**.

In the next step, we have demonstrated the possibility of removing the protective Boc group under mild conditions without acid or base catalysis (Scheme 4). Deprotection occurs in high, near quantitative yields, resulting in glutarimides with a heterocyclic fragment at the α-position **1a**–**e** – structures in demand for the design of CRBN ligands and immunomodulatory drugs.

**Scheme 5:**
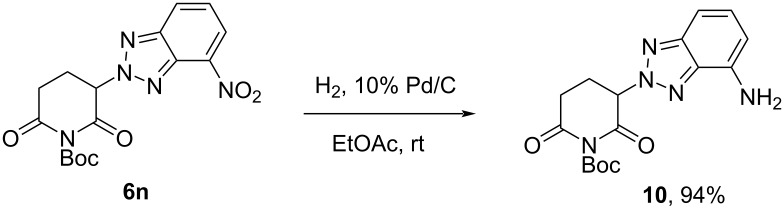
Preparation of NH_2_-containing derivative **10** via reduction of **6n**.

In compound **6n**, catalytic hydrogenation was used to reduce the nitro group, resulting in the production of a benzotriazole analog of pomalidomide/lenalidomide precursor **10** with a high yield (Scheme 5). In the near future, after removal of the protective group, the biological profile of the compound obtained will be studied (i.e., antimyeloid activity, degradation of transcription factors IKZF1/2/3, antiangiogenic activity, and cytokine secretion), which will be reported in the following works.

## Conclusion

In summary, a new diazo reagent for the convenient incorporation of heterocyclic substituents at the alpha-position of glutarimide by Rh(II)-catalyzed insertion of carbene into the N–H bond of nitrogen heterocycles has been proposed. The method allows the preparation of modified glutarimides with a wide range of aromatic and aliphatic NH-heterocycles under mild conditions in moderate to high yields. It is shown that electron-rich substrates tend to give C–H insertion products. The N-modification of tetrazoles and 1,2,4-triazoles using a diazocarbonyl reagent is presented for the first time. The protective group is removed without acid catalysis with near quantitative yields. New benzotriazole derivatives containing functional groups capable of participating in the subsequent modification for linker attachment to assemble the PROTAC molecule have been obtained.

## Supporting Information

Deposition number 2298240 (for **6n**) contain the supplementary crystallographic data for this paper. These data are provided free of charge by the joint Cambridge Crystallographic Data Centre and Fachinformationszentrum Karlsruhe Access Structures service https://www.ccdc.cam.ac.uk/structures.

File 1 General experimental information, X-ray crystallographic data, synthetic procedures, analytical data and NMR spectra for the reported compounds.
