# Supplementary material for: N-Boc-α-diazo glutarimide as efficient reagent for assembling N-heterocycle-glutarimide diads via Rh(II)-catalyzed N–H insertion reaction
Source: Beilstein J Org Chem. 2023 Dec 7;19:1841–8. doi: 10.3762/bjoc.19.136 (PMC10714472; doi:10.3762/bjoc.19.136)

BEILSTEIN JOURNAL OF ORGANIC CHEMISTRY

Supporting Information

for

N-Boc-α-diazo glutarimide as efficient reagent for assembling N-heterocycle-glutarimide diads via Rh(II)-catalyzed N–H insertion reaction

Grigory Kantin, Pavel Golubev, Alexander Sapegin, Alexander Bunev and Dmitry Dar’in

Beilstein J. Org. Chem. **2023,** *19,* 1841–1848. doi:10.3762/bjoc.19.136

General experimental information, X-ray crystallographic data, synthetic procedures, analytical data and NMR spectra for the reported compounds

License and Terms: This is a supporting information file under the terms of the Creative Commons Attribution License (https://creativecommons.org/licenses/by/4.0). Please note that the reuse, redistribution and reproduction in particular requires that the author(s) and source are credited and that individual graphics may be subject to special legal provisions.
The license is subject to the *Beilstein Journal of Organic Chemistry* terms and conditions: (https://www.beilstein-journals.org/bjoc/terms)

Table of contents

1. Experimental procedures and characterization data S2

2. Crystallographic data for compound **6n**……………………………………………..……………..……S13

3. Copies of ^{1}H, ^{13}C NMR, ^{19}F and NOESY NMR spectra S15

1. Experimental procedures and characterization data

General considerations. All reagents were used as purchased from commercial suppliers without further purification. Dichloromethane (DCM) was freshly distilled over P_2O_5. NMR spectra were recorded using Bruker Avance III spectrometer in $CDCl_3$ or DMSO-d_6 (^{1}H: 400.13 MHz; ^{13}C: 100.61 MHz; ^{19}F 376.50 MHz); chemical shifts are reported as parts per million (δ, ppm); the residual solvent peaks were used as internal standard: 7.26 or 2.5 for ^{1}H and 77.16 or 39.7 ppm for ^{13}C in $CDCl_3$ or DMSO-d_6, respectively; multiplicities are abbreviated as follows: s = singlet, d = doublet, t = triplet, q = quartet, m = multiplet, dd = doublet of doublets, dt = doublet of triplets, ddd = doublet/doublets of doublets; coupling constants, *J*, are reported in Hz. Mass spectra were recorded using a Bruker microTOF spectrometer (ionization by electrospray, positive ions detection). Melting points were determined in open capillary tubes on a Stuart SMP50 Automatic Melting Point Apparatus. Analytical thin-layer chromatography was carried out on UV-254 silica gel plates using appropriate eluents. Compounds were visualized with short-wavelength UV light. Column chromatography was performed using silica gel Merk grade 60 (0.040–0.063 mm) 230–400 mesh.

Synthesis of diazo reagent 5

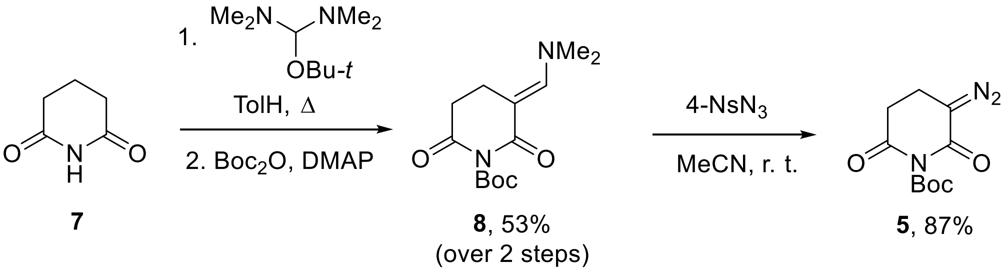

***tert*-Butyl (*E*)-3-((dimethylamino)methylene)-2,6-dioxopiperidine-1-carboxylate (8).** Glutarimide **7**

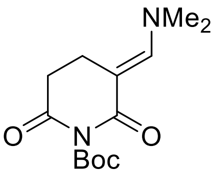

(4.52 g, 0.04 mol) was placed in a 100 mL round bottom flask, followed by 30 mL of anhydrous toluene and Bredereck's reagent (9.07 mL, 0.044 mol). The resulting mixture was stirred at 75 °C for 5 h. After cooling to room temperature, the precipitate was filtered off, washed with 2 × 5 mL of toluene and 2 × 10 mL of petroleum ether and dried in air to obtain 3.59 g of light-yellow crystals which were used in the next step.

α-Dimethylaminomethylene glutarimide (3.57 g, 0.021 mol), Boc_2O (5.0 g, 0.023 mol) and DMAP (0.122 g, 1.0 mmol) were mixed with 40 mL of anhydrous DCM in a 100 mL round bottom flask. The reaction mixture was stirred overnight at room temperature. The product was isolated by column chromatography (80 g silica, eluted with DCM). Yield: 5.63 g (53%). Light-yellow solid; m.p. 139.1–140.3 °C. ^{1}H NMR (400 MHz, $CDCl_3$) δ 7.50 (s, 1 H), 3.11 (s, 6 H), 2.84–2.81 (m, 2 H), 2.59–2.55 (m, 2 H), 1.57 (s, 9 H) ppm. ^{13}C{^{1}H} NMR (101 MHz, $CDCl_3$) δ 170.3, 167.1, 150.8, 150.3, 90.6, 84.9, 43.5, 32.2, 27.5, 19.4 ppm. HRMS (ESI), *m/z* calcd for $C_{13}H_{20}N_2NaO_4$ $[M+Na]^+$ 291.1315 found 291.1302.

***tert*-Butyl 3-diazo-2,6-dioxopiperidine-1-carboxylate (5).** Boc-protected dimethylaminomethylene

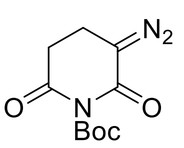

glutarimide (**8**) (1.072 g, 4 mmol) was dissolved in 8 mL of anhydrous acetonitrile. *p*-Nosylazide (0.958 g, 4.2 mmol) was added, and the mixture was stirred overnight at room temperature. Volatiles were evaporated, and the residue was purified by column chromatography (40 g silica, eluted with 10–50% acetone in *n*-hexane). Yield: 0.83 g (87%). Light-yellow or greenish yellow solid; m.p. 92.7–93.1 °C. ^{1}H NMR (400 MHz, DMSO-d_6) δ 2.92–2.88 (m, 2 H), 2.78–2.74 (m, 2 H), 1.48 (s, 9 H) ppm. ^{13}C{^{1}H} NMR (101 MHz, DMSO-d_6) δ 169.5. 164.5, 149.0, 85.9, 55.8, 30.8, 27.5, 15.5 ppm. HRMS (ESI), *m/z* calcd for $C_{10}H_{13}N_3NaO_4$ [M+Na]$^+$ 262.0798 found 262.0801.

General procedure GP1 for the preparation of compounds 6a–z, 9a,c,i,z.

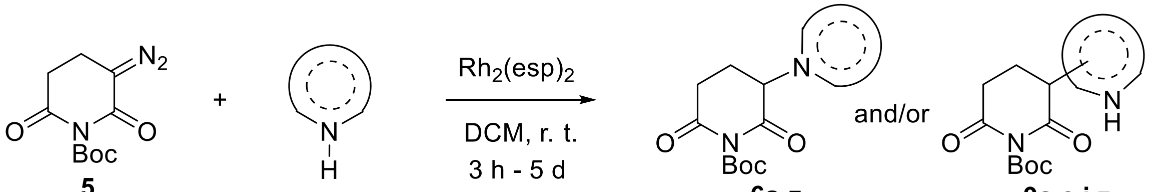

To a solution/suspension of the corresponding NH-substrate (0.33 mmol, 1 equiv) in DCM (1 mL) was added the catalyst solution (2.5 mM $Rh_2(esp)_2$ in DCM, 100 µL, 0.06 mol %). To the vigorously stirred mixture the solution of diazo reagent **5** (96 mg, 0.4 mmol, 1.2 equiv) in DCM (1 mL) was added during 1–2 minutes. The reaction mixture was stirred at ambient temperature until full consumption of starting material (controlled by TLC). If starting materials were present after 16 h additional portion of the catalyst solution was added and stirring was continued for another day. Upon full conversion of diazo regent, the reaction mixture was directly subjected to column chromatography on silica gel (*n*-hexane–acetone) to afford pure product.

***tert*-Butyl 3-(1*H*-indol-1-yl)-2,6-dioxopiperidine-1-carboxylate (6a).** Prepared according to GP1 using

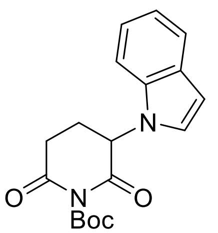

indole as NH-substrate; reaction time – 16 h; eluent – *n*-hexane–acetone (10% to 40% of acetone). Yield: 51 mg (47%). White solid; m.p. 145.7–148.5 °C (decomp.). ^{1}H NMR (400 MHz, $CDCl_3$) δ 7.69–7.66 (m, 1 H), 7.27–7.26 (m, 2 H), 7.19–7.15 (m, 1 H), 7.09 (d, J = 3.3 Hz, 1 H), 6.64 (d, J = 3.3 Hz, 1 H), 5.18 (dd, J = 12.7, 5.0 Hz, 1 H), 3.05–2.99 (m, 1 H), 2.95–2.86 (m, 1 H), 2.71–2.60 (m, 1 H), 2.43–2.36 (m, 1 H), 1.59 (s, 9 H) ppm. ^{13}C{^{1}H} NMR (101 MHz, $CDCl_3$) δ 168.6, 167.1, 147.9, 136.2, 128.7, 125.4, 122.4, 121.5, 120.3, 109.1, 103.7, 87.2, 55.9, 31.6, 27.4, 24.4 ppm. HRMS (ESI), *m/z* calcd for $C_{18}H_{20}N_2NaO_4$ [M+Na]$^+$ 351.1315 found 351.1306.

***tert*-Butyl 3-(1*H*-indol-3-yl)-2,6-dioxopiperidine-1-carboxylate (9a).** Yield: 24 mg (22%). Pale yellow

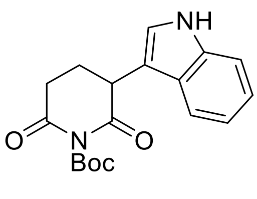

amorphous solid. ^{1}H NMR (400 MHz, $CDCl_3$) δ 8.39 (br. s, 1 H), 7.55 (d, J = 7.9 Hz, 1 H), 7.38 (d, J = 8.1 Hz, 1 H), 7.25–7.21 (m, 1 H), 7.17–7.13 (m, 1 H), 7.01–7.00 (m, 1 H), 4.20–4.17 (m, 1 H), 2.74–2.70 (m, 2 H), 2.39–2.28 (m, 2 H), 1.62 (s, 9 H) ppm. ^{13}C{^{1}H} NMR (101 MHz, $CDCl_3$) δ 171.4, 170.4, 149.3, 136.4, 126.1, 122.6, 122.4, 119.9, 118.6, 111.7, 111.1, 86.5, 39.9, 30.6, 27.5, 24.2 ppm. HRMS (ESI), *m/z* calcd for $C_{18}H_{20}N_2NaO_4$ [M+Na]$^+$ 351.1315 found 351.1305.

Methyl 1-(1-(*tert*-butoxycarbonyl)-2,6-dioxopiperidin-3-yl)-1*H*-indole-3-carboxylate (6b). Prepared according to GP1 using methyl indole-3-carboxylate as NH-substrate; reaction time – 16 h; eluent – *n*-hexane–acetone (10% to 40% of acetone). Yield: 102 mg (80%). White solid; m.p. 147.9–149.2 °C (decomp.). ^{1}H NMR (400 MHz, $CDCl_3$) δ 8.26–8.22 (m, 1 H), 7.85 (s, 1 H), 7.34–7.29 (m, 3 H), 5.22 (dd, J = 13.0, 5.0 Hz, 1 H), 3.93 (s, 3 H), 3.10–3.03 (m, 1 H), 2.98-2.89 (m, 1 H), 2.74–2.63 (m, 1 H), 2.48-2.41 (m, 1 H), 1.59 (s, 9 H) ppm. ^{13}C{^{1}H} NMR (101 MHz, $CDCl_3$) δ 168.2, 166.3, 165.1, 147.7, 136.7, 132.0, 126.5, 123.6, 122.6, 122.2, 109.6, 109.4, 87.5, 56.2, 51.2, 31.5, 27.4, 24.3 ppm. HRMS (ESI), *m/z* calcd for $C_{20}H_{22}N_2NaO_6$ $[M+Na]^+$ 409.1370 found 409.1370.

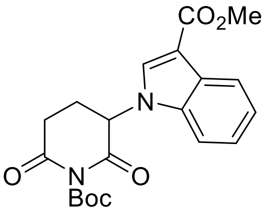

***tert*-Butyl 3-(5-(methoxycarbonyl)-1*H*-pyrrol-3-yl)-2,6-dioxopiperidine-1-carboxylate (9c).** Prepared according to GP1 using methyl pyrrole-2-carboxylate as NH-substrate; reaction time – 16 h; eluent – *n*-hexane–acetone (10% to 40% of acetone). Yield: 22 mg (20%). White amorphous solid. ^{1}H NMR (400 MHz, $CDCl_3$) δ 9.92 (br. s, 1 H), 6.89–6.87 (m, 1 H), 6.14–6.12 (m, 1 H), 3.94–3.90 (m, 1 H), 3.85 (s, 3 H), 2.89 (dt, J = 17.6, 5.1 Hz, 1 H), 2.79–2.71 (m, 1 H), 2.48–2.30 (m, 2 H), 1.58 (s, 9 H) ppm. ^{13}C{^{1}H} NMR (101 MHz, $CDCl_3$) δ 169.9, 169.4, 161.4, 148.5, 130.8, 123.2, 115.5, 108.6, 86.9, 51.5, 40.4, 31.0, 27.4, 22.4 ppm. HRMS (ESI), *m/z* calcd for $C_{16}H_{20}N_2NaO_6$ $[M+Na]^+$ 359.1213 found 359.1210.

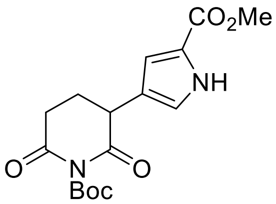

***tert*-Butyl 3-(3,5-dimethyl-1*H*-pyrazol-1-yl)-2,6-dioxopiperidine-1-carboxylate (6d).** Prepared according to GP1 using 3,5-dimethylpyrazole as NH-substrate; reaction time – 16 h; eluent – *n*-hexane–acetone (10% to 40% of acetone). Yield: 90 mg (89%). White solid; m.p. 162.3–163.3 °C. ^{1}H NMR (400 MHz, $CDCl_3$) δ 5.90 (s, 1 H), 4.85 (dd, J = 11.4, 5.1 Hz, 1 H), 3.10 (dt, J = 17.1, 4.0 Hz, 1 H), 2.98–2.88 (m, 1 H), 2.84–2.76 (m, 1 H), 2.41–2.34 (m, 1 H), 2.28 (s, 3 H), 2.22 (s, 3 H), 1.56 (s, 9 H) ppm. ^{13}C{^{1}H} NMR (101 MHz, $CDCl_3$) δ 168.9, 166.8, 149.0, 148.0, 140.8, 106.1, 86.9, 56.9, 31.1, 27.4, 23.3, 13.6, 11.0 ppm. HRMS (ESI), *m/z* calcd for $C_{15}H_{22}N_3O_4$ $[M+H]^+$ 308.1605 found 308.1617.

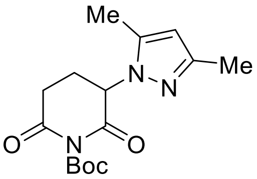

***tert*-Butyl 3-(3,5-diphenyl-1*H*-pyrazol-1-yl)-2,6-dioxopiperidine-1-carboxylate (6e).** Prepared according to GP1 using 3,5-diphenylpyrazole as NH-substrate; reaction time – 16 h; eluent – *n*-hexane–acetone (10% to 40% of acetone). Yield: 112 mg (74%). White solid; m.p. 129.3–131.4 °C. ^{1}H NMR (400 MHz, $CDCl_3$) δ 7.87–7.85 (m, 2 H), 7.54–7.50 (m, 5 H), 7.45–7.41 (m, 2 H), 7.37–7.32 (m, 1 H), 6.68 (s, 1 H), 5.07 (dd, J = 10.4, 4.9 Hz, 1 H), 3.13 (dt, J = 17.5, 4.6 Hz, 1 H), 3.03–2.93 (m, 1 H), 2.77–2.69 (m, 1 H), 2.39–2.32 (m, 1 H), 1.60 (s, 9 H) ppm. ^{13}C{^{1}H} NMR (101 MHz, $CDCl_3$) δ 169.1, 166.9, 151.9, 148.1, 146.8, 132.9, 129.9, 129.3, 129.14, 129.09, 128.6, 128.1, 125.9, 103.9, 86.9, 57.4, 30.7, 27.5, 24.0 ppm. HRMS (ESI), *m/z* calcd for $C_{25}H_{25}N_3NaO_4$ $[M+Na]^+$ 454.1737 found 454.1745.

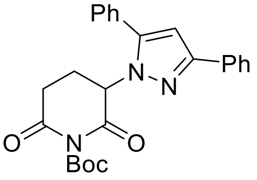

***tert*-Butyl 3-(4-bromo-3,5-dimethyl-1*H*-pyrazol-1-yl)-2,6-dioxopiperidine-1-carboxylate (6f).**

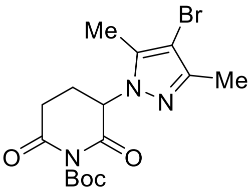

Prepared according to GP1 using 4-bromo-3,5-dimethylpyrazole as NH-substrate; reaction time – 16 h; eluent – *n*-hexane–acetone (10% to 40% of acetone). Yield: 120 mg (94%). White solid; m.p. 237.1–239.2 °C. ^{1}H NMR (400 MHz, $CDCl_3$) δ 4.93 (dd, *J* = 11.2, 5.0 Hz, 1 H), 3.13–3.07 (m, 1 H), 2.96–2.77 (m, 2 H), 2.40–2.34 (m, 1 H), 2.29 (s, 3 H), 2.23 (s, 3 H), 1.57 (s, 9 H) ppm. $^{13}C\{^1H\}$ NMR (101 MHz, $CDCl_3$) δ 168.7, 166.3, 147.8, 147.6, 139.1, 95.6, 87.1, 57.9, 30.9, 27.4, 23.0, 12.4, 10.5 ppm. HRMS (ESI), *m/z* calcd for $C_{15}H_{21}N_3O_4Br$ $[M+H]^+$ 386.0710/388.689 found 386.0711/388.0692.

The of compound **6f** synthesis was run according to GP1 on a gram scale (3.3 mmol); yield 1.19 g (93%).

***tert*-Butyl 3-(2*H*-indazol-2-yl)-2,6-dioxopiperidine-1-carboxylate (6g).** Prepared according to GP1 using indazole as NH-substrate; reaction time – 16 h; eluent – *n*-hexane–acetone (10% to 40% of acetone). Yield: 100 mg (92%). White solid; m.p. 143.2–144.6 °C. ^{1}H NMR (400 MHz, $CDCl_3$) δ 8.06 (s, 1 H), 7.72–7.69 (m, 2 H), 7.34–7.31 (m, 1 H), 7.14–7.11 (m, 1 H), 5.33 (dd, *J* = 10.7, 4.5 Hz, 1 H), 3.13 (dt, *J* = 17.2, 4.5 Hz, 1 H), 3.03–2.94 (m, 1 H), 2.90–2.82 (m, 1 H), 2.58–2.52 (m, 1 H), 1.57 (s, 9 H) ppm. $^{13}C\{^1H\}$ NMR (101 MHz, $CDCl_3$) δ 168.6, 166.0, 147.8, 126.8, 124.3, 122.4, 121.8, 120.4, 117.7, 87.2, 61.8, 30.7, 27.4, 24.2 ppm. HRMS (ESI), *m/z* calcd for $C_{17}H_{20}N_3O_4$ $[M+H]^+$ 330.1449 found 330.1453.

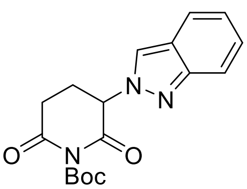

***tert*-Butyl 2,6-dioxo-3-(2*H*-pyrazolo[3,4-*b*]pyridin-2-yl)piperidine-1-carboxylate (6h).** Prepared according to GP1 using pyrazolo[3,4-*b*]pyridine as NH-substrate; reaction time – 3 days, after 16 h additional portion of catalyst was added; eluent – *n*-hexane–acetone (10% to 40% of acetone). Yield: 36 mg (33%). Pale grey amorphous solid. ^{1}H NMR (400 MHz, $CDCl_3$) δ 8.73 (s, 1 H), 8.17 (s, 1 H), 8.11–8.01 (m, 1H), 7.09 (dd, *J* = 8.4, 4.1 Hz, 1 H), 5.50 (dd, *J* = 10.7, 5.0 Hz, 1 H), 3.25–3.13 (m, 1 H), 3.11–3.00 (m, 1 H), 2.98–2.86 (m, 1 H), 2.69–2.54 (m, 1 H), 1.55 (s, 9 H) ppm. $^{13}C\{^1H\}$ NMR (101 MHz, $CDCl_3$) δ 168.7, 165.9, 158.1, 152.2, 147.8, 130.4, 124.9, 118.4, 114.1, 87.3, 62.2, 30.6, 27.4, 23.9 ppm. HRMS (ESI), *m/z* calcd for $C_{16}H_{19}N_4O_4$ $[M+H]^+$ 331.1401 found 331.1399.

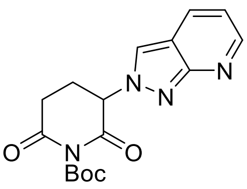

***tert*-Butyl 3-(1*H*-imidazol-5-yl)-2,6-dioxopiperidine-1-carboxylate (9i).** Prepared according to GP1 using imidazole as NH-substrate; reaction time – 3 days, after 16 h additional portion of catalyst was added; eluent – *n*-hexane–acetone (15% to 80% of acetone). Yield: 26 mg (28%). Pale grey amorphous solid. ^{1}H NMR (400 MHz, $CDCl_3$) δ 8.27 (s, 1 H), 8.13 (s, 1 H), 6.98 (s, 1 H), 4.91 (s, 1 H), 3.11–3.03 (m, 1 H), 2.75 (dt, *J* = 18.1, 5.2 Hz, 1 H), 2.47–2.39 (m, 1 H), 2.36–2.26 (m, 1 H), 1.53 (s, 9 H) ppm. $^{13}C\{^1H\}$ NMR (101 MHz, $CDCl_3$) δ 171.8, 166.9, 150.3, 134.7, 127.9, 127.2, 83.6, 36.5, 30.7, 27.9, 24.3 ppm. HRMS (ESI), *m/z* calcd for $C_{13}H_{18}N_3O_4$ $[M+H]^+$ 280.1292 found 280.1283.

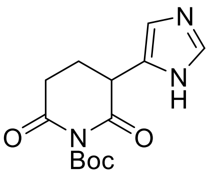

***tert*-Butyl 3-(1*H*-benzo[*d*]imidazol-1-yl)-2,6-dioxopiperidine-1-carboxylate (6j).** Prepared according to GP1 using benzimidazole as NH-substrate; reaction time – 2 days, after 16 h additional portion of catalyst was added; eluent – *n*-hexane–acetone (10% to 40% of acetone). Yield: 43 mg (39%). Grey amorphous solid. ^{1}H NMR (400 MHz, $CDCl_3$) δ 8.06 (s, 1 H), 7.91–7.77 (m, 1 H), 7.40–7.29 (m, 3 H), 5.29 (dd, *J* = 13.2, 4.9 Hz, 1 H), 3.05 (ddd, *J* = 17.8, 4.8, 2.6 Hz, 1 H), 2.94 (ddd, *J* = 17.9, 12.9, 5.0 Hz, 1 H), 2.74 (qd, *J* = 13.0, 4.8 Hz, 1 H), 2.49–2.38 (m, 1 H), 1.58 (s, 9 H) ppm. $^{13}C\{^{1}H\}$ NMR (101 MHz, $CDCl_3$) δ 168.2, 166.0, 147.6, 143.3, 141.8, 123.8, 123.0, 120.7, 109.9, 87.6, 55.5, 31.4, 27.4, 24.2 ppm. HRMS (ESI), *m/z* calcd for $C_{17}H_{20}N_3O_4$ $[M+H]^+$ 330.1448 found 330.1447.

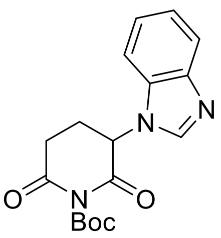

***tert*-Butyl 2,6-dioxo-3-(1*H*-1,2,4-triazol-1-yl)piperidine-1-carboxylate (6l).** Prepared according to GP1 using 1,2,4-triazole as NH-substrate; reaction time – 4 days, after 16 h and 40 h 2 additional portions of catalyst were sequentially added; eluent – *n*-hexane–acetone (10% to 40% of acetone). Yield: 74 mg (80%). White solid; m.p. 128.4–132.5 °C. ^{1}H NMR (400 MHz, $CDCl_3$) δ 8.34 (br. s, 1 H), 8.03 (br. s, 1 H), 5.24-5.22 (m, 1 H), 3.12–3.03 (m, 1 H), 2.91–2.76 (m, 2 H), 2.53–2.49 (m, 1 H), 1.57 (s, 9 H) ppm. $^{13}C\{^{1}H\}$ NMR (101 MHz, $CDCl_3$) δ 168.2, 165.5, 152.2, 147.6, 140.8, 87.5, 58.4, 30.6, 27.4, 23.6 ppm. HRMS (ESI), *m/z* calcd for $C_{12}H_{17}N_4O_4$ $[M+H]^+$ 281.1245 found 281.1247.

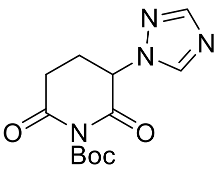

***tert*-Butyl 3-(2*H*-benzotriazol-2-yl)-2,6-dioxopiperidine-1-carboxylate (6m).** Prepared according to GP1 using benzotriazole as NH-substrate; reaction time – 5 h; eluent – *n*-hexane–acetone (10% to 40% of acetone). Yield: 103 mg (89%). White amorphous solid. ^{1}H NMR (400 MHz, $CDCl_3$) δ 7.92–7.88 (m, 2 H), 7.46–7.42 (m, 2 H), 5.83 (dd, *J* = 10.5, 5.0 Hz, 1 H), 3.15–3.02 (m, 2 H), 2.95–2.86 (m, 1 H), 2.67–2.59 (m, 1 H), 1.58 (s, 9 H) ppm. $^{13}C\{^{1}H\}$ NMR (101 MHz, $CDCl_3$) δ 168.3, 164.9, 147.5, 144.7, 127.2, 118.3, 87.3, 64.7, 30.3, 27.4, 23.9 ppm. HRMS (ESI), *m/z* calcd for $C_{16}H_{19}N_4O_4$ $[M+H]^+$ 331.1401 found 331.1392.

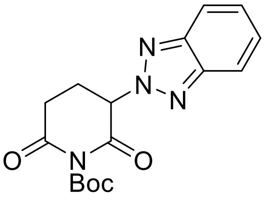

***tert*-Butyl 3-(4-nitro-2*H*-benzotriazol-2-yl)-2,6-dioxopiperidine-1-carboxylate (6n).** Prepared according to GP1 using 1*H*-4-nitrobenzotriazole as NH-substrate; reaction time – 5 h; eluent – *n*-hexane–acetone (10% to 40% of acetone). Yield: 62 mg (50%). Grey amorphous solid. ^{1}H NMR (400 MHz, $CDCl_3$) δ 8.47–8.45 (m, 1 H), 8.33–8.31 (m, 1 H), 7.63–7.59 (m, 1 H), 6.02 (dd, *J* = 11.5, 5.0 Hz, 1 H), 3.25–3.12 (m, 2 H), 3.02–2.93 (m, 1 H), 2.74–2.67 (m, 1 H), 1.57 (s, 9 H) ppm. $^{13}C\{^{1}H\}$ NMR (101 MHz, $CDCl_3$) δ 167.9, 164.4, 147.3, 146.7, 138.2, 137.4, 126.5, 125.8, 125.4, 87.6, 65.6, 30.4, 27.4, 23.9 ppm. HRMS (ESI), *m/z* calcd for $C_{16}H_{17}N_5NaO_6$ $[M+Na]^+$ 398.1071 found 398.1073.

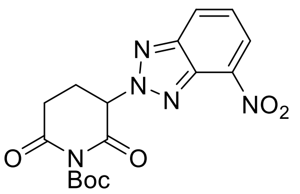

2,2,2-Trifluoroethyl 2-(1-(*tert*-butoxycarbonyl)-2,6-dioxopiperidin-3-yl)-2*H*-benzotriazole-5-carboxylate (6o). Prepared according to GP1 using 1*H*-4-nitrobenzotriazole as NH-substrate; reaction time – 5 h; eluent – *n*-hexane–acetone (10% to 40% of acetone). Yield: 84 mg (56%). White amorphous solid. ^{1}H NMR (400 MHz, $CDCl_3$) δ 8.77 (dd, *J* = 1.5, 0.9 Hz, 1 H), 8.09 (dd, *J* = 9.0, 1.5 Hz, 1 H), 7.98 (dd, *J* = 9.0, 0.9 Hz, 1 H), 5.88 (dd, *J* = 10.8, 4.9 Hz, 1 H), 4.78 (q, *J* = 8.4 Hz,

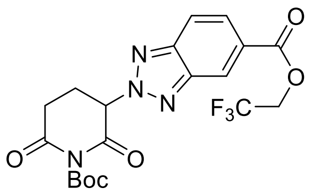

2 H), 3.17–3.06 (m, 2 H), 2.97–2.87 (m, 1 H), 2.70–2.63 (m, 1 H), 1.58 (s, 9 H) ppm. $^{13}C\{^{1}H\}$ NMR (101 MHz, $CDCl_3$) δ 168.0, 164.5, 164.4, 147.3, 146.7, 143.9, 127.3, 126.9, 123.1, 123.0 (q, J = 275.6 Hz), 118.9, 87.5, 61.1 (q, J = 36.5 Hz), 30.3, 27.4, 23.9 ppm. ^{19}F NMR (376.5 MHz, $CDCl_3$) δ –73.61 ppm. HRMS (ESI), *m/z* calcd for $C_{19}H_{19}N_4NaO_6F_3$ $[M+Na]^+$ 479.1149 found 479.1150.

***tert*-Butyl 2,6-dioxo-3-(5-phenyl-2*H*-tetrazol-2-yl)piperidine-1-carboxylate (6p).** Prepared according to GP1 using 5-phenyltetrazole as NH-substrate; reaction time – 16 h; eluent – *n*-hexane–acetone (10% to 40% of acetone). Yield: 60 mg (51%). White solid; m.p. 149.4–150.4 °C. ^{1}H NMR (400 MHz, $CDCl_3$) δ 8.19–8.16 (m, 2 H), 7.53–7.49 (m, 3 H), 5.89–5.85 (m, 1 H), 3.16–3.06 (m, 1 H), 3.04–2.90 (m, 2 H), 2.65–2.59 (m, 1 H), 1.58 (s, 9 H) ppm. $^{13}C\{^{1}H\}$ NMR (101 MHz, $CDCl_3$) δ 167.9, 165.7, 163.9, 147.3, 130.7, 128.9, 127.1, 126.8, 87.6, 61.8, 30.4, 27.4, 23.4 ppm. HRMS (ESI), *m/z* calcd for $C_{17}H_{19}N_5NaO_4$ $[M+Na]^+$ 380.1329 found 380.1329.

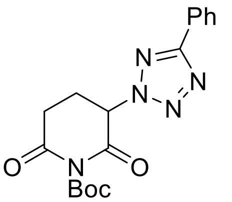

***tert*-Butyl 3-(5-(2-methoxyethyl)-2*H*-tetrazol-2-yl)-2,6-dioxopiperidine-1-carboxylate (6q).** Prepared according to GP1 using 5-(2-methoxyethyl)tetrazole as NH-substrate; reaction time – 2 days, after 16 h additional portion of catalyst was added; eluent – *n*-hexane–acetone (10% to 40% of acetone). Yield: 65 mg (58%). Colorless viscous oil. ^{1}H NMR (400 MHz, $CDCl_3$) δ 5.79–5.75 (m, 1 H), 3.83 (t, J = 6.7 Hz, 2 H), 3.39 (s, 3 H), 3.22 (t, J = 6.7 Hz, 2 H), 3.11–3.06 (m, 1 H), 2.99–2.86 (m, 2 H), 2.62–2.54 (m, 1 H), 1.58 (s, 9 H) ppm. $^{13}C\{^{1}H\}$ NMR (101 MHz, $CDCl_3$) δ 167.8, 164.9, 163.9, 147.2, 87.6, 69.8, 61.6, 58.8, 30.4, 27.4, 26.4, 23.3 ppm. HRMS (ESI), *m/z* calcd for $C_{14}H_{22}N_5O_5$ $[M+H]^+$ 340.1616 found 340.1609.

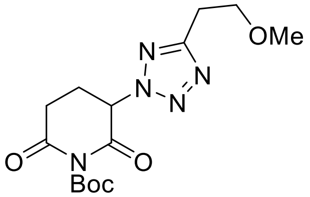

***tert*-Butyl 3-(4-ethoxycarbonylpiperidin-1-yl)-2,6-dioxopiperidine-1-carboxylate (6r).** Prepared according to GP1 using ethyl isonipecotate as NH-substrate; reaction time – 5 days, after 16 h and 40 h 2 additional portions of catalyst were sequentially added; eluent – *n*-hexane–acetone (10% to 40% of acetone). Yield: 22 mg (18%). Colorless oil. ^{1}H NMR (400 MHz, $CDCl_3$) δ 4.15 (q, J = 7.2 Hz, 2 H), 3.37 (dd, J = 10.4, 4.3 Hz, 1 H), 2.94–2.82 (m, 3 H), 2.79–2.73 (m, 1 H), 2.66–2.53 (m, 2 H), 2.37–2.29 (m, 1 H), 2.21–2.01 (m, 2 H), 1.96–1.92 (m, 2 H), 1.81–1.71 (m, 2 H), 1.57 (s, 9 H), 1.26 (t, J = 7.2 Hz, 3 H) ppm. $^{13}C\{^{1}H\}$ NMR (101 MHz, $CDCl_3$) δ 174.8, 169.9, 169.6, 148.7, 86.4, 64.4, 60.4, 50.5, 47.8, 40.9, 30.8, 28.7, 28.4, 27.5, 21.0, 14.2 ppm. HRMS (ESI), *m/z* calcd for $C_{18}H_{29}N_2O_6$ $[M+H]^+$ 369.2019 found 369.2011.

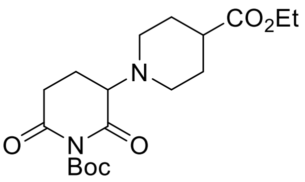

***tert*-Butyl 3-(azepan-1-yl)-2,6-dioxopiperidine-1-carboxylate (6s).** Prepared according to GP1 using hexamethyleneimine as NH-substrate; reaction time – 5 days, after 16 h and 40 h 2 additional portions of catalyst were sequentially added; eluent – *n*-hexane–acetone (10% to 40% of acetone). Yield: 13 mg (13%). Colorless oil. ^{1}H NMR (400 MHz, $CDCl_3$) δ 3.55–3.51 (m, 1 H), 2.98–2.93 (m, 2 H), 2.87–2.78 (m, 3 H), 2.68–2.59 (m, 1 H), 2.14–2.08 (m, 2 H), 1.74–1.59 (m, 8 H), 1.58 (s, 9 H) ppm. $^{13}C\{^{1}H\}$ NMR (101 MHz, $CDCl_3$) δ 171.1, 169.9, 148.9, 86.3, 65.9, 52.5, 31.6, 29.7, 27.5, 27.0, 22.9 ppm. HRMS (ESI), *m/z* calcd for $C_{16}H_{27}N_2O_4$ $[M+H]^+$ 311.1966 found 311.1967.

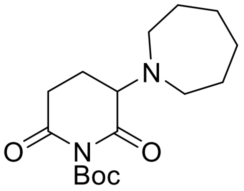

***tert*-Butyl 3-(indolin-1-yl)-2,6-dioxopiperidine-1-carboxylate (6t).** Prepared according to GP1 using indoline as NH-substrate; reaction time – 16 h; eluent – *n*-hexane–acetone (10% to 40% of acetone). Yield: 101 mg (87%). White solid; m.p. 151.9–153.4 °C. ^1H NMR (400 MHz, $CDCl_3$) δ 7.13–7.11 (m, 1 H), 7.10–7.05 (m, 1 H), 6.75–6.71 (m, 1 H), 6.46 (d, *J* = 7.8 Hz, 1 H), 4.38 (dd, *J* = 12.6, 5.1 Hz, 1 H), 3.56–3.45 (m, 2 H), 3.16–3.01 (m, 2 H), 2.99–2.93 (m, 1 H), 2.84–2.75 (m, 1 H), 2.36–2.18 (m, 2 H), 1.57 (s, 9 H) ppm. $^{13}C\{^1H\}$ NMR (101 MHz, $CDCl_3$) δ 169.2, 168.1, 149.9, 148.3, 129.5, 127.3, 124.9, 118.8, 107.1, 86.7, 56.6, 48.8, 31.9, 28.4, 27.5, 21.5 ppm. HRMS (ESI), *m/z* calcd for $C_{18}H_{23}N_2O_4$ $[M+H]^+$ 331.1653 found 331.1651.

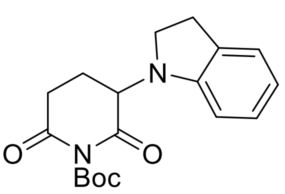

***tert*-Butyl 3-(5-acetylindolin-1-yl)-2,6-dioxopiperidine-1-carboxylate (6u).** Prepared according to GP1 using 5-acetylindoline as NH-substrate; reaction time – 16 h; eluent – *n*-hexane–acetone (10% to 40% of acetone). Yield: 116 mg (94%). White solid; m.p. 168.2–171.5 °C. ^1H NMR (400 MHz, $CDCl_3$) δ 7.76–7.73 (m, 2 H), 6.40 (d, *J* = 8.2 Hz, 1 H), 4.46 (dd, *J* = 13.0, 5.0 Hz, 1 H), 3.67–3.52 (m, 2 H), 3.20–3.04 (m, 2 H), 3.02–2.95 (m, 1 H), 2.89–2.79 (m, 1 H), 2.50 (s, 3 H), 2.40–2.29 (m, 1 H), 2.28–2.18 (m, 1 H), 1.56 (s, 9 H) ppm. $^{13}C\{^1H\}$ NMR (101 MHz, $CDCl_3$) δ 196.4, 168.9, 167.6, 154.9, 148.1, 130.3, 129.3, 128.3, 125.2, 105.1, 86.9, 55.9, 48.6, 31.8, 27.5, 27.4, 26.2, 22.0 ppm. HRMS (ESI), *m/z* calcd for $C_{20}H_{25}N_2O_5$ $[M+H]^+$ 373.1758 found 373.1757.

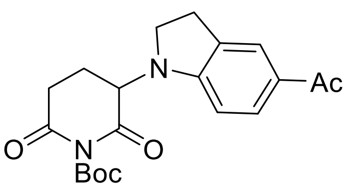

***tert*-Butyl 3-(5-(*N*,*N*-dimethylsulfamoyl)indolin-1-yl)-2,6-dioxopiperidine-1-carboxylate (6v).** Prepared according to GP1 using *N*,*N*-dimethylindoline-5-sulfonamide as NH-substrate; reaction time – 16 h; eluent – *n*-hexane–acetone (10% to 40% of acetone). Yield: 133 mg (92%). White solid; m.p. 157.1–158.2 °C. ^1H NMR (400 MHz, $CDCl_3$) δ 7.48–7.45 (m, 1 H), 7.42 (m, 1 H), 6.43 (d, *J* = 8.3 Hz, 1 H), 4.46 (dd, *J* = 12.9, 4.9 Hz, 1 H), 3.68–3.62 (m, 1 H), 3.60–3.54 (m, 1 H), 3.20–3.05 (m, 2 H), 3.01–2.95 (m, 1 H), 2.91–2.82 (m, 1 H), 2.67 (s, 6 H), 2.38–2.21 (m, 2 H), 1.56 (s, 9 H) ppm. $^{13}C\{^1H\}$ NMR (101 MHz, $CDCl_3$) δ 168.9, 167.7, 154.4, 148.1, 129.8, 129.1, 124.4, 123.6, 105.4, 86.9, 55.7, 48.5, 38.0, 31.8, 27.6, 27.4, 22.0 ppm. HRMS (ESI), *m/z* calcd for $C_{20}H_{28}N_3O_6S$ $[M+H]^+$ 438.1694 found 438.1699.

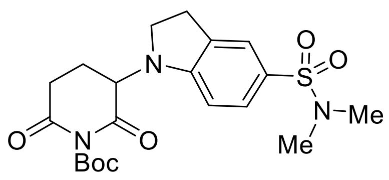

***tert*-Butyl 3-(6-nitroindolin-1-yl)-2,6-dioxopiperidine-1-carboxylate (6w).** Prepared according to GP1 using 6-nitroindoline as NH-substrate; reaction time – 16 h; eluent – *n*-hexane–acetone (10% to 40% of acetone). Yield: 110 mg (89%). Yellow solid; m.p. >250 °C. ^1H NMR (400 MHz, $CDCl_3$) δ 7.61 (dd, *J* = 8.0, 1.8 Hz, 1 H), 7.21 (d, *J* = 1.8 Hz, 1 H), 7.18–7.15 (m, 1 H), 4.50–4.46 (m, 1 H), 4.48 (dd, *J* = 12.8, 4.9 Hz, 1 H), 3.69–3.63 (m, 1 H), 3.60–3.53 (m, 1 H), 3.25–3.07 (m, 2 H), 3.03–2.98 (m, 1 H), 2.93–2.84 (m, 1 H), 2.40–2.24 (m, 2 H), 1.56 (s, 9 H) ppm. $^{13}C\{^1H\}$ NMR (101 MHz, $CDCl_3$) δ 168.8, 167.7, 151.9, 148.5, 148.0, 137.0, 124.5, 114.5, 100.8, 87.0, 56.0, 48.7, 31.9, 28.3, 27.4, 22.2 ppm. HRMS (ESI), *m/z* calcd for $C_{18}H_{22}N_3O_6$ $[M+H]^+$ 376.1503 found 376.1499.

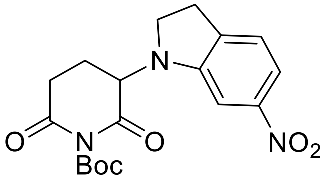

***tert*-Butyl 3-(tetrahydroquinolin-1-yl)-2,6-dioxopiperidine-1-carboxylate (6x).** Prepared according to GP1 using 1,2,3,4-tetrahydroquinoline as NH-substrate; reaction time – 16 h; eluent – *n*-hexane–acetone (10% to 40% of acetone). Yield: 73 mg (64%). White solid; m.p. 141.6–142.1 °C. ^{1}H NMR (400 MHz, $CDCl_3$) δ 7.08–7.04 (m, 1 H), 7.03–7.00 (m, 1 H), 6.70–6.66 (m, 1 H), 6.56 (d, *J* = 8.2 Hz, 1 H), 4.61 (dd, *J* = 12.7, 4.9 Hz, 1 H), 3.24 (t, J = 5.8 Hz, 2 H), 2.95 (ddd, J = 17.7, 4.5, 2.7 Hz, 1 H), 2.87–2.73 (m, 3 H), 2.42 (qd, J = 13.2, 4.5 Hz, 1 H), 2.18–2.11 (m, 1 H), 2.09–1.93 (m, 2 H), 1.59 (s, 9 H) ppm. ^{13}C{^{1}H} NMR (101 MHz, $CDCl_3$) δ 169.3, 168.9, 148.4, 144.2, 129.6, 127.0, 124.2, 117.4, 110.9, 86.6, 58.9, 45.4, 32.1, 28.0, 27.5, 22.4, 21.5 ppm. HRMS (ESI), *m/z* calcd for $C_{19}H_{25}N_2O_4$ $[M+H]^+$ 345.1809 found 345.1801.

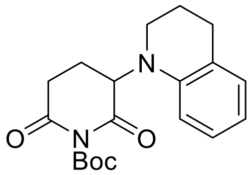

***tert*-Butyl 3-(6-bromotetrahydroquinolin-1-yl)-2,6-dioxopiperidine-1-carboxylate (6y).** Prepared according to GP1 using 6-bromo-1,2,3,4-tetrahydroquinoline as NH-substrate; reaction time – 16 h; eluent – *n*-hexane–acetone (10% to 40% of acetone). Yield: 118 mg (84%). White solid; m.p. 173.8–174.9 °C. ^{1}H NMR (400 MHz, $CDCl_3$) δ 7.14–7.12 (m, 2 H), 6.44 (d, *J* = 8.4 Hz, 1 H), 4.54 (dd, *J* = 12.8, 4.8 Hz, 1 H), 3.27–3.17 (m, 2 H), 2.96 (ddd, *J* = 17.7, 4.5, 2.5 Hz, 1 H), 2.85–2.68 (m, 3 H), 2.42 (qd, *J* = 13.2, 4.5 Hz, 1 H), 2.17–2.11 (m, 1 H), 2.06–1.92 (m, 2 H), 1.58 (s, 9 H) ppm. ^{13}C{^{1}H} NMR (101 MHz, $CDCl_3$) δ 169.0, 168.6, 148.3, 143.4, 131.9, 129.6, 126.4, 112.7, 109.3, 86.8, 59.0, 45.3, 32.1, 27.8, 27.5, 22.1, 21.6 ppm. HRMS (ESI), *m/z* calcd for $C_{19}H_{24}BrN_2O_4$ $[M+H]^+$ 423.0914/425.0893 found 423.0913/423.0895.

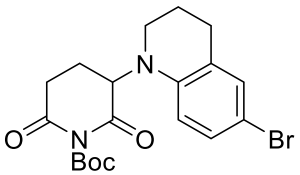

***tert*-Butyl 3-(10,11-dihydro-5*H*-dibenzo[*b,f*]azepin-2-yl)-2,6-dioxopiperidine-1-carboxylate (9z).** Prepared according to GP1 using 10,11-dihydro-5*H*-dibenzo[*b,f*]azepine as NH-substrate; reaction time – 16 h; eluent – *n*-hexane–acetone (10% to 40% of acetone). Yield: 62 mg (46%). White amorphous solid. ^{1}H NMR (400 MHz, $CDCl_3$) δ 7.12–7.05 (m, 2 H), 6.93–6.89 (m, 2 H), 6.83–6.73 (m, 3 H), 3.76–3.72 (m, 1 H), 3.10–3.05 (m, 4 H), 2.79 (dt, *J* = 17.7, 5.4 Hz, 1 H), 2.73–2.64 (m, 1 H), 2.28–2.21 (m, 2 H), 1.61 (s, 9 H) ppm. ^{13}C{^{1}H} NMR (101 MHz, $CDCl_3$) δ 171.5, 170.1, 149.1, 142.2, 142.0, 130.6, 130.4, 128.8, 128.7, 127.1, 126.9, 126.4, 119.8, 118.5, 118.1, 86.4, 47.0, 35.1, 34.7, 31.0, 27.5, 25.3 ppm. HRMS (ESI), *m/z* calcd for $C_{24}H_{27}N_2O_4$ $[M+H]^+$ 407.1966 found 407.1957.

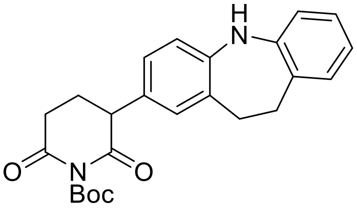

Additional experiments on catalysts screening

General procedure for testing of Rh(II) catalysts in the reaction of 5 with indole.

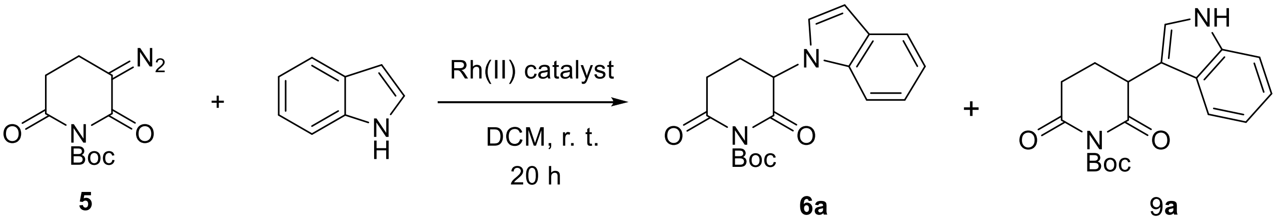

To a solution of indole (19 mg, 0.165 mmol, 1 equiv.) in DCM (0.5 mL) was added the Rh(II) catalyst ($Rh_2(TFA)_4$ or $Rh_2(OAc)_4$, 1 mol %). To the vigorously stirred mixture the solution of diazo reagent **5** (48 mg, 0.2 mmol, 1.2 equiv.) in DCM (0.5 mL) was added during 1-2 minutes. The reaction mixture was stirred at ambient temperature until full consumption of starting material (20 h, controlled by TLC). To the reaction mixture was added 2,4-dinitrotoluene (27 mg, 0.15 mmol) as the internal NMR standard, the solvent was evaporated and the residue was redissolved in $CDCl_3$ (1 mL) followed by registration of ^{1}H NMR spectrum.

Table S1. Results of testing $Rh_2(TFA)_4$ and $Rh_2(OAc)_4$ catalysts for the reaction 5→6a/9a.

Entry	Rh(II) catalyst	NMR yield of **6a**	NMR yield of **6a**	Ratio **6a/9a**
1	$Rh_2(TFA)_4$	11%	21%	34:66
2	$Rh_2(OAc)_4$	40%	22%	64:36

General procedure for testing of Cu(II) catalysts in the reaction of 5 with ethyl isonipecotate.

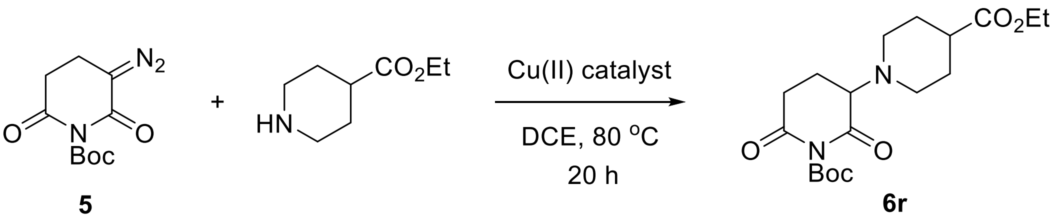

To a solution of ethyl isonipecotate (26 mg, 0.165 mmol, 1 equiv.) in DCE (0.5 mL) were added the Cu(II) catalyst ($Cu(OTf)_2$, 5 mol% or $Cu(acac)_2$, 10 mol %) and the solution of diazo reagent **5** (48 mg, 0.2 mmol, 1.2 equiv.) in DCE (0.5 mL) . The mixture was vigorously stirred in a closed vessel at 80 °C until full consumption of starting material (20 h, controlled by TLC). Upon cooling to ambient temperature to the reaction mixture was added 2,4-dinitrotoluene (27 mg, 0.15 mmol) as the internal NMR standard, the solvent was evaporated and the residue was redissolved in $CDCl_3$ (1 mL) followed by registration of ^{1}H NMR spectrum.

Table S2. Results of testing $Cu(OTf)_2$ and $Cu(acac)_2$ catalysts for the reaction 5→6r.

Entry	Cu(II) catalyst	NMR yield of **6r**
1	$Cu(OTf)_2$ (5 mol%)	0%
2	$Cu(acac)_2$ (10 mol %)	25%

General procedure GP1 for the preparation of compounds 1a-e by Boc-group removal

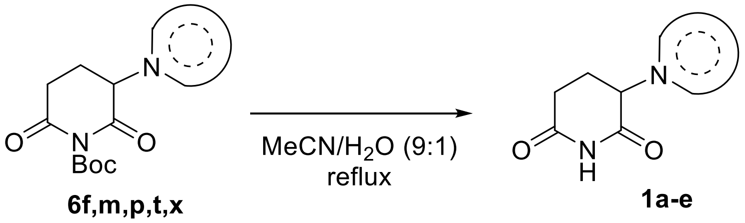

6f,m,p,t,x **1a-e**

To a solution of the corresponding *N*-Boc-protected substrate **6** (0.10 mmol) in a mixture of MeCN and H_2O (9:1, 700 μL) was heated at reflux for 8 hours. Then, the solvents were removed under reduced pressure and the resulting substance was dried in high vacuum at ambient temperature.

3-(2*H*-Benzo[*d*][1,2,3]triazol-2-yl)piperidine-2,6-dione (1a). Prepared according to GP2 using compound **6m**. Yield: 23 mg (99%). White solid; m.p. > 250 °C. ^{1}H NMR (400 MHz, DMSO-d_6) δ 11.29 (s, 1H), 8.01–7.91 (m, 2H), 7.52–7.43 (m, 2H), 6.23–6.11 (m, 1H), 3.05 – 2.53 (m, 4H) ppm. NMR data are in accordance with previously reported.[1] HRMS (ESI), *m/z* calcd for $C_{11}H_{11}N_4O_2$ $[M+H]^+$ 231.0877 found 231.0890.

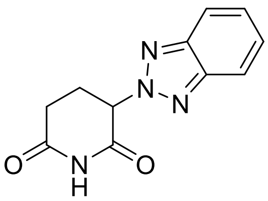

3-(4-Bromo-3,5-dimethyl-1*H*-pyrazol-1-yl)piperidine-2,6-dione (1b). Prepared according to GP2 using compound **6f**. Yield: 28 mg (97%). White solid; m.p. 241.7–243.5 °C. ^{1}H NMR (400 MHz, DMSO-d_6) δ 11.04 (br. s, 1H), 5.34 (dd, *J* = 12.1, 5.1 Hz, 1H), 2.85–2.72 (m, 1H), 2.70–2.54 (m, 2H), 2.21 (s, 3H), 2.19–2.15 (m, 1H), 2.10 (s, 3H) ppm. ^{13}C{^{1}H} NMR (101 MHz, DMSO-d_6) δ 173.1, 170.6, 145.6, 139.4, 93.6, 58.0, 31.1, 23.8, 12.6, 10.4 ppm. HRMS (ESI), *m/z* calcd for $C_{10}H_{13}BrN_3O_2$ $[M+H]^+$ 286.0186 found 286.0188.

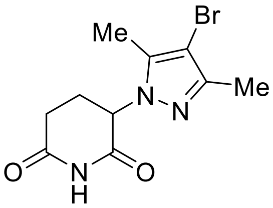

3-(5-Phenyl-2*H*-tetrazol-2-yl)piperidine-2,6-dione (1c). Prepared according to GP2 using compound **6p**. Yield: 24 mg (95%). White solid; m.p. 197.4–199.1 °C. ^{1}H NMR (400 MHz, DMSO-d_6) δ 11.38 (br. s, 1H), 8.14–8.03 (m, 2H), 7.65–7.50 (m, 3H), 6.34 (dd, *J* = 12.2, 5.0 Hz, 1H), 2.99– .70 (m, 3H), 2.60–2.48 (m, 1H) ppm. ^{13}C{^{1}H} NMR (101 MHz, DMSO-d_6) δ 172.6, 168.8, 164.6, 131.2, 129.8 (2C), 127.1, 126.8 (2C), 62.5, 30.8, 24.1 ppm. HRMS (ESI), *m/z* calcd for $C_{12}H_{12}N_5O_2$ $[M+H]^+$ 258.0986 found 258.0983.

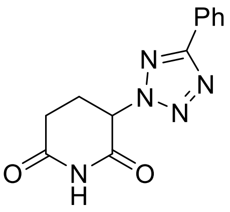

3-(Indolin-1-yl)piperidine-2,6-dione (1d). Prepared according to GP2 compound **6t**. Yield: 21 mg (93%). White solid; m.p. 146.6–148.4 °C. ^{1}H NMR (400 MHz, Acetone-d_6) δ 9.55 (br. s, 1H), 7.09–6.80 (m, 2H), 6.80–6.36 (m, 2H), 4.65 (dd, J = 13.0, 5.0 Hz, 1H), 3.64–3.37 (m, 2H), 3.11–2.70 (m, 4H), 2.35 (qd, J = 13.1, 4.5 Hz, 1H), 2.23–2.09 (m, 1H) ppm. $^{13}C\{^{1}H\}$ NMR (101 MHz, DMSO-d_6) δ 173.5, 172.0, 151.9, 129.1, 127.3, 124.6, 117.3, 107.0, 55.3, 48.1, 32.0, 28.2, 22.2 ppm. HRMS (ESI), *m/z* calcd for $C_{13}H_{15}N_2O_2$ $[M+H]^+$ 231.1128 found 231.1125.

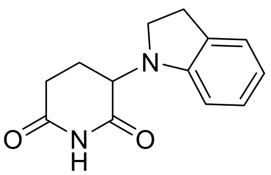

3-(3,4-Dihydroquinolin-1(2*H*)-yl)piperidine-2,6-dione (1e). Prepared according to GP2 using compound **6x**. Yield: 22 mg (90%). White solid; m.p. 155.4–157.0 °C. ^{1}H NMR (400 MHz, DMSO-d_6) δ 10.79 (br. s, 1H), 6.99–6.87 (m, 2H), 6.68 (d, J = 8.3 Hz, 1H), 6.51 (t, J = 7.2 Hz, 1H), 4.86 (dd, J = 12.7, 4.9 Hz, 1H), 3.15 (dt, J = 11.5, 5.3 Hz, 1H), 3.06 (dt, J = 11.5, 5.3 Hz, 1H), 2.86 (ddd, J = 17.2, 13.6, 5.3 Hz, 1H), 2.75–2.61 (m, 2H), 2.61–2.53 (m, 1H), 2.39–2.21 (m, 1H), 1.93–1.76 (m, 3H) ppm. $^{13}C\{^{1}H\}$ NMR (101 MHz, DMSO-d_6) δ 173.5, 172.7, 145.5, 129.4, 127.0, 122.9, 116.2, 111.4, 57.7, 44.7, 32.0, 28.1, 22.2, 22.0 ppm. HRMS (ESI), *m/z* calcd for $C_{14}H_{17}N_2O_2$ $[M+H]^+$ 245.1285 found 245.1289.

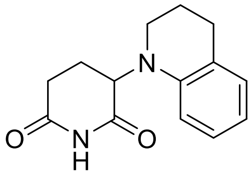

Preparation of compound 10

***tert*-Butyl 3-(4-amino-2*H*-benzo[*d*][1,2,3]triazol-2-yl)-2,6-dioxopiperidine-1-carboxylate (10).** In a 5 mL Schlenk flask, compound **6n** (30 mg, 0.1 mmol), 10% Pt/C catalyst (3 mg) and 600 μL EtOAc were added. The mixture was stirred at room temperature for 12 h under H_2 atmosphere. After the reaction was completed, the solution was filtered through a pad of Celite. The filtrate was concentrated under reduced pressure to obtain 27 mg (94%) of the titled compound as grey oil. ^{1}H NMR (400 MHz, $CDCl_3$) δ 7.25–7.16 (m, 2H), 6.50 (dd, J = 5.5, 2.5 Hz, 1H), 5.79 (dd, J = 10.8, 5.1 Hz, 1H), 4.38–3.69 (m, 3H), 3.13–2.94 (m, 2H), 2.94–2.79 (m, 1H), 2.61–2.47 (m, 1H), 1.57 (s, 9H) ppm. $^{13}C\{^{1}H\}$ NMR (101 MHz, $CDCl_3$) δ 168.5, 165.1, 147.6, 145.6, 137.7, 137.3, 128.6, 106.7, 106.3, 87.3, 64.4, 30.3 27.41, 23.8nppm. HRMS (ESI), *m/z* calcd for $C_{16}H_{20}N_5O_4$ $[M+H]^+$ 346.1510 found 346.1513.

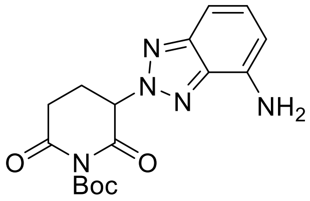

Crystallographic data for compounds 6n

X-ray Single Crystal analysis was performed on SuperNova, Single source at offset/far, HyPix3000 diffractometer. Crystal growth was performed by slow evaporation of solution in *n*-hexane/acetone mixture (1:1) at 5 °C. The crystal was kept at 100 K during data collection. Using Olex2 [2], the structure was solved with the SHELXD [3] structure solution program using Dual Space and refined with the SHELXL[4] refinement package using Least Squares minimization. CCDC 2298240 (**6n**) contains the supplementary crystallographic data for this paper. These data can be obtained free of charge from The Cambridge Crystallographic Data Centre via https://www.ccdc.cam.ac.uk/.

Table S1. Crystal data and ORTEP representation for **6n** (2298240)

**Empirical Formula**	$C_{16}H_{17}N_5O_6$
**Formula weight**	500.46
**Temperature, K**	99.99(10)
**Crystal system**	monoclinic
**Space group**	$P2_1/n$
**a/Å**	5.5905(2)
**b/Å**	27.5779(9)
**c/Å**	11.1992(4)
**α/°**	90
**β/°**	93.357(3)
**γ/°**	90
**Volume/Å^3**	1723.67(10)
**Z**	3
**ρ_{calc}g/cm^3**	1.446
**μ/mm^{-1}**	0.960
**F(000)**	784
**Radiation**	CuKα (λ = 1.54184)
**2Θ range for data collection/°**	6.41 to 138.636
**Index ranges**	$-6 \leq h \leq 6$, $-33 \leq k \leq 33$, $-11 \leq l \leq 13$
**Reflections collected**	15668
**Independent reflections**	3211 [R_{int} = 0.0448, R_{sigma} = 0.0369]
**Data/restraints/parameters**	3211/0/298
**Goodness-of-fit on F^2**	1.063
**Final R indexes [I>=2σ (I)]**	R_1 = 0.0975, wR_2 = 0.2355
**Final R indexes [all data]**	R_1 = 0.1097, wR_2 = 0.2426
**Largest diff. peak/hole / e Å^{-3}**	0.37/–0.38

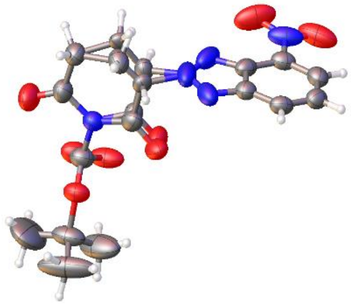

Figure S1. ORTEP representation of compound **6n** (thermal ellipsoids are shown at 50% probability)

References

1. Krasavin, M.; Bubyrev, A.; Kazantsev, A.; Heim, C.; Maiwald, S.; Zhukovsky, D.; Dar'in, D.; Hartmann, M. D.; Bunev, A. *J. Enz. Inh.Med. Chem.* **2022**, *37*(1), 527–530.
2. Dolomanov, O. V; Bourhis, L. J.; Gildea, R. J.; Howard, J. A. K.; Puschmann, H. *J. Appl. Crystallogr.* **2009**, *42*, 339–341.
3. Sheldrick, G. M. *Acta Crystallogr. Sect. A* **2008**, *64*, 112–122.
4. Sheldrick, G. M. *Acta Crystallogr. Sect. C* **2015**, *71*, 3–8.

1. Copies of ^{1}H, ^{13}C NMR, ^{19}F and NOESY NMR spectra

Copies of ^{1}H (400.13 MHz, $CDCl_3$) and $^{13}C\{^{1}H\}$ (100.61 MHz, $CDCl_3$) spectra of **8**

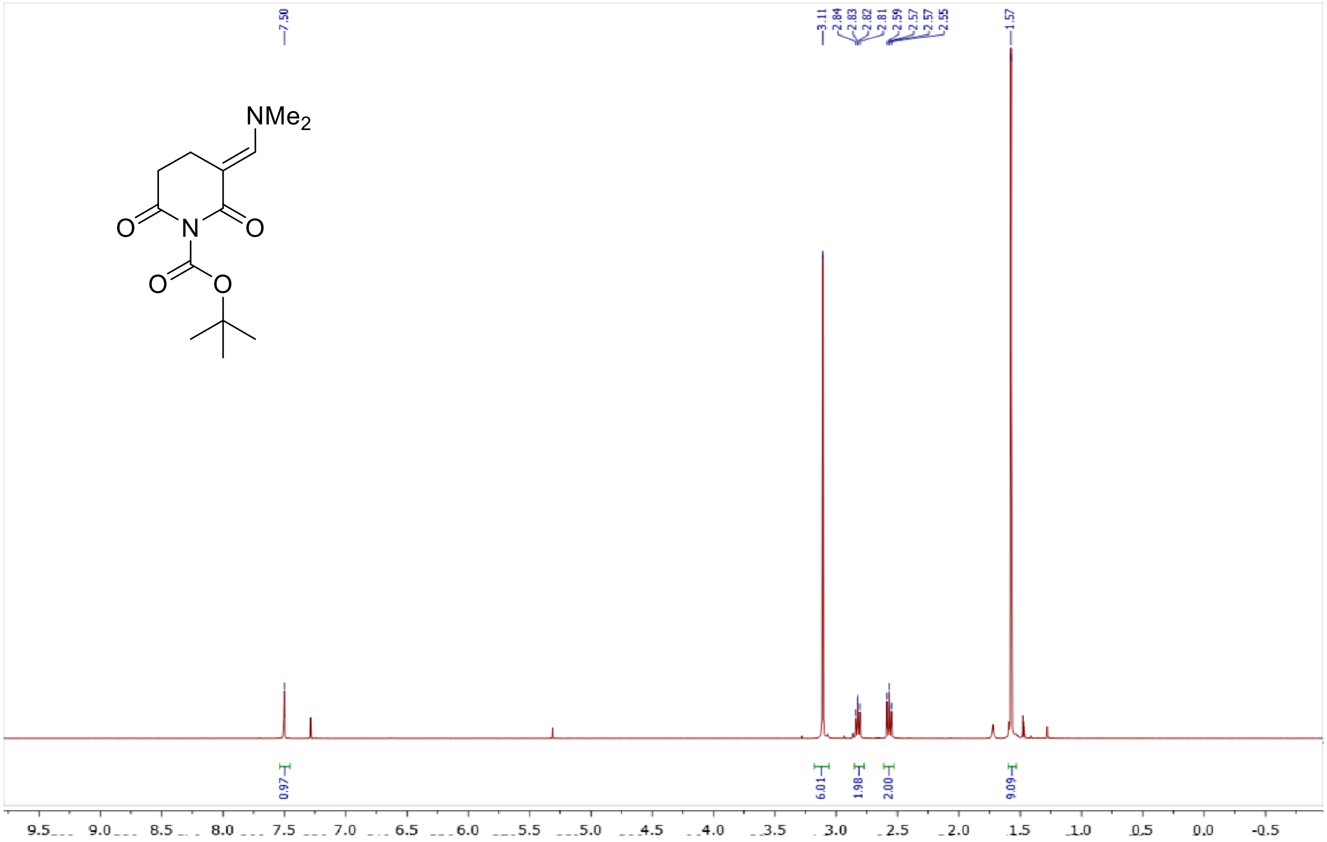

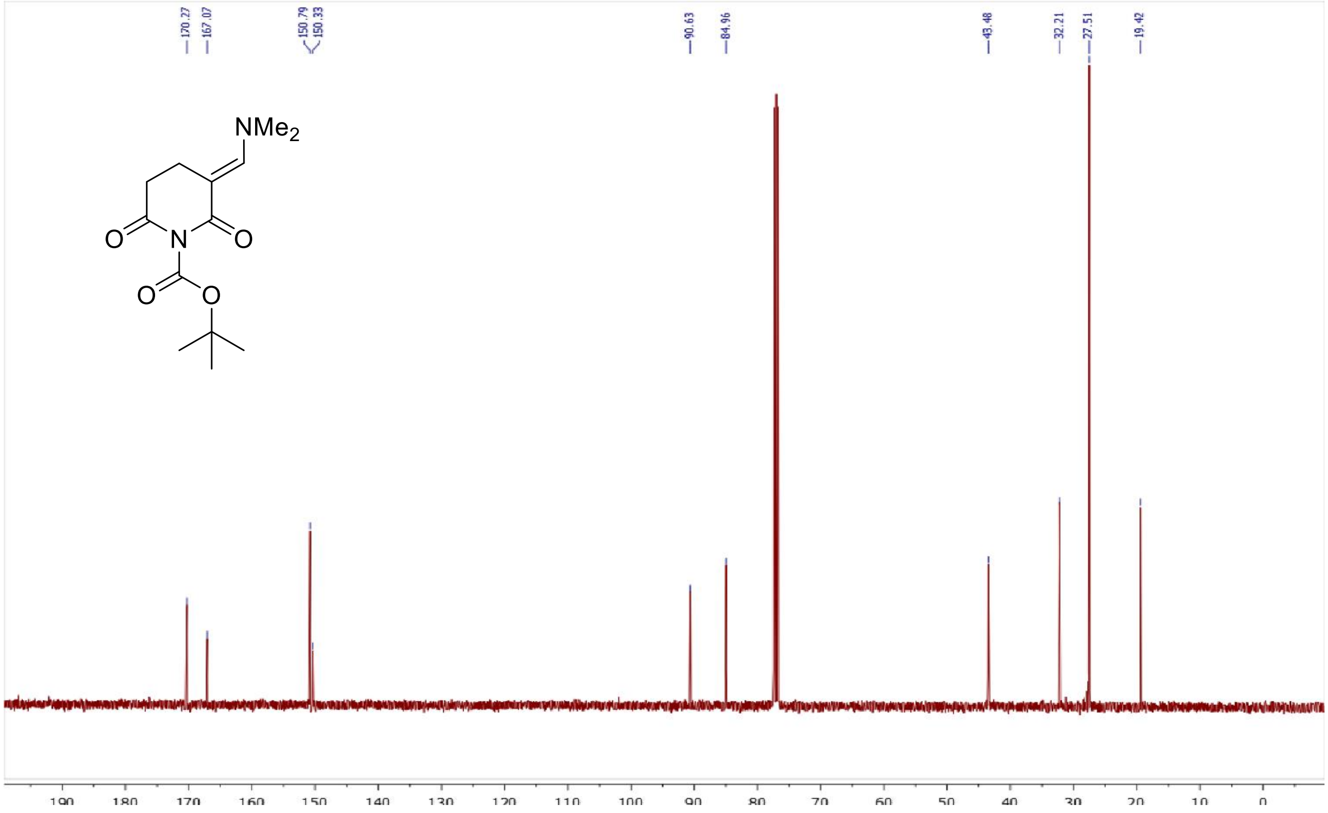

Copies of ^{1}H (400.13 MHz, DMSO-d_6) and $^{13}C\{^{1}H\}$ (100.61 MHz, DMSO-d_6) spectra of **5**

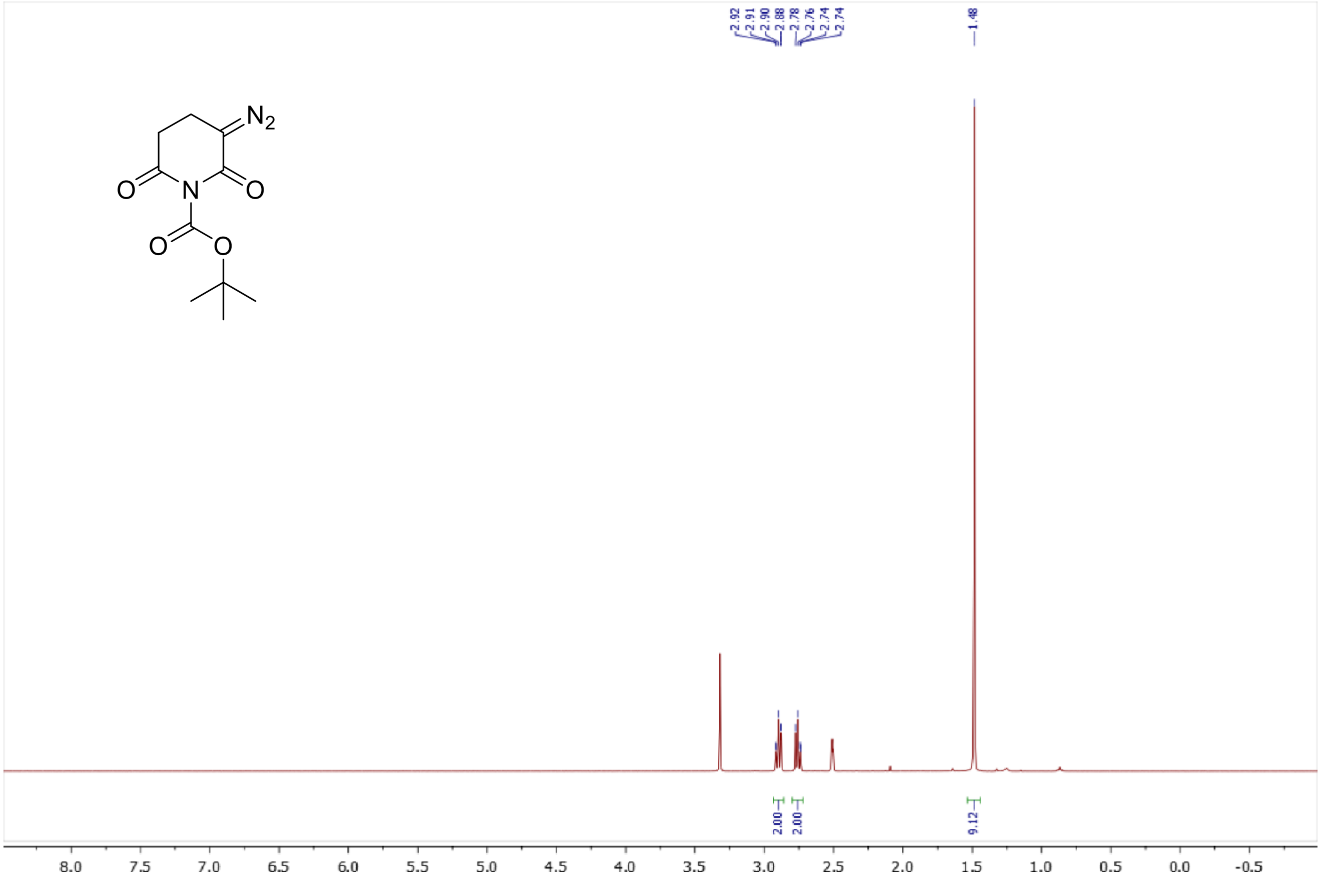

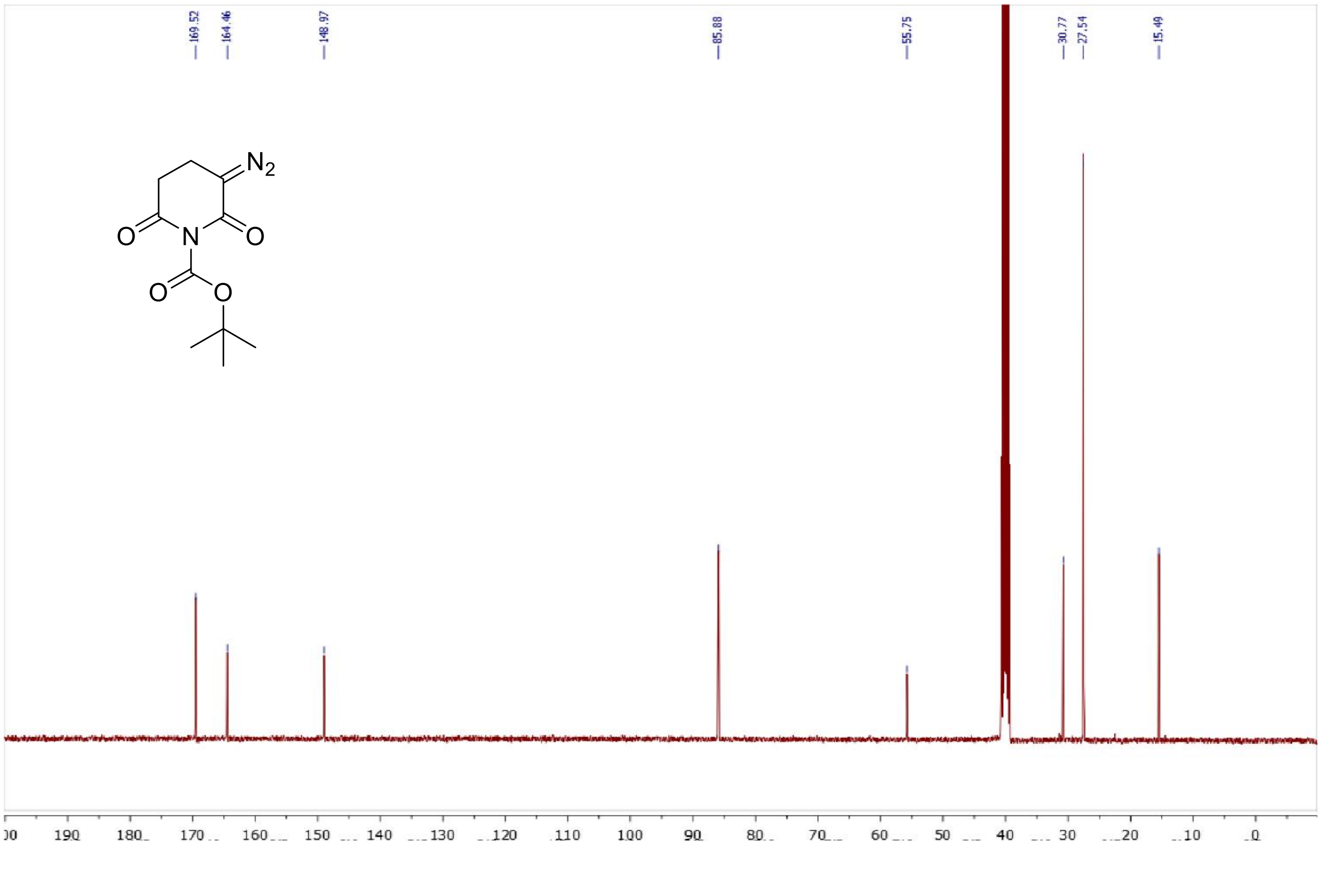

Copies of ^{1}H (400.13 MHz, $CDCl_3$) and $^{13}C\{^{1}H\}$ (100.61 MHz, $CDCl_3$) spectra of **6a**

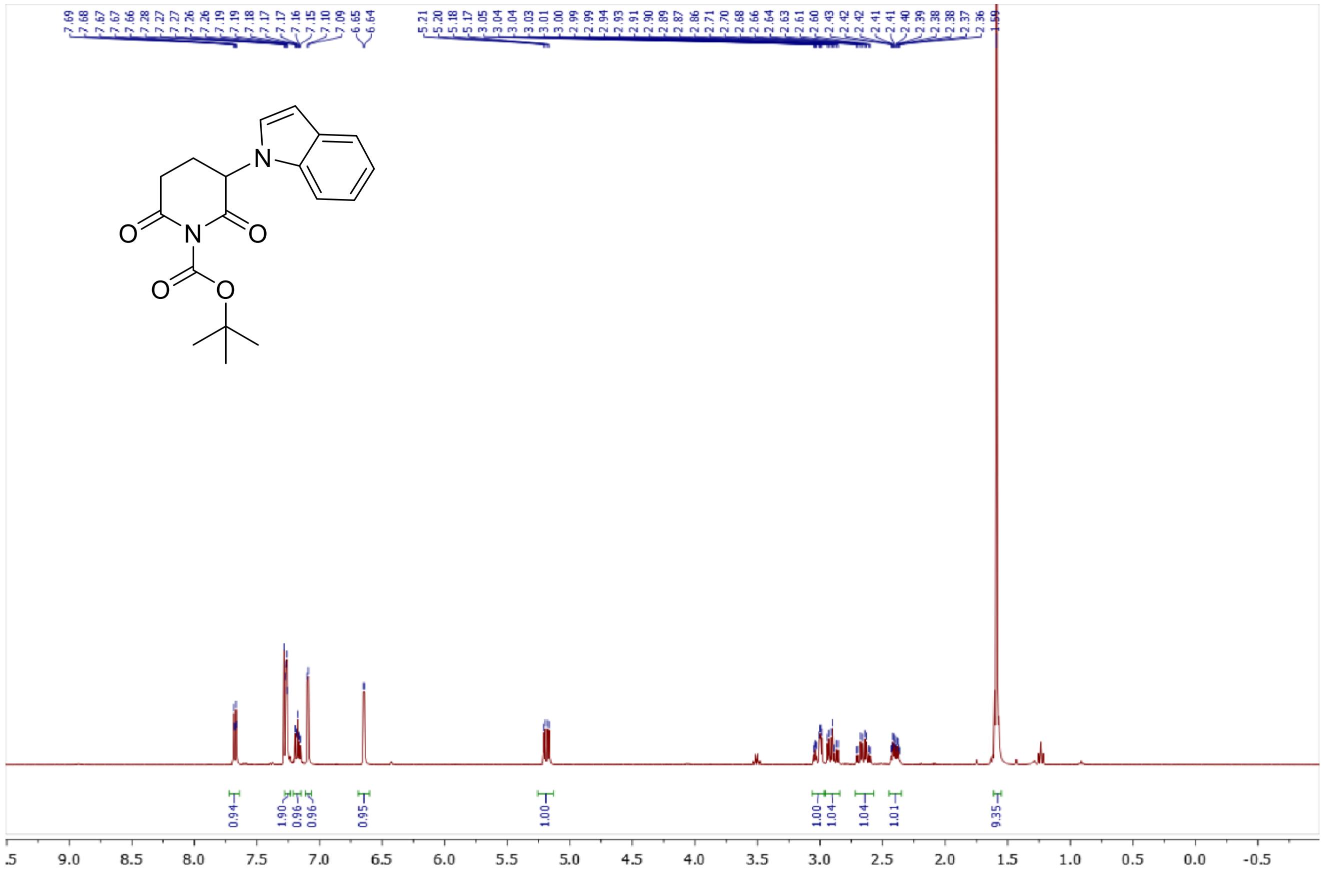

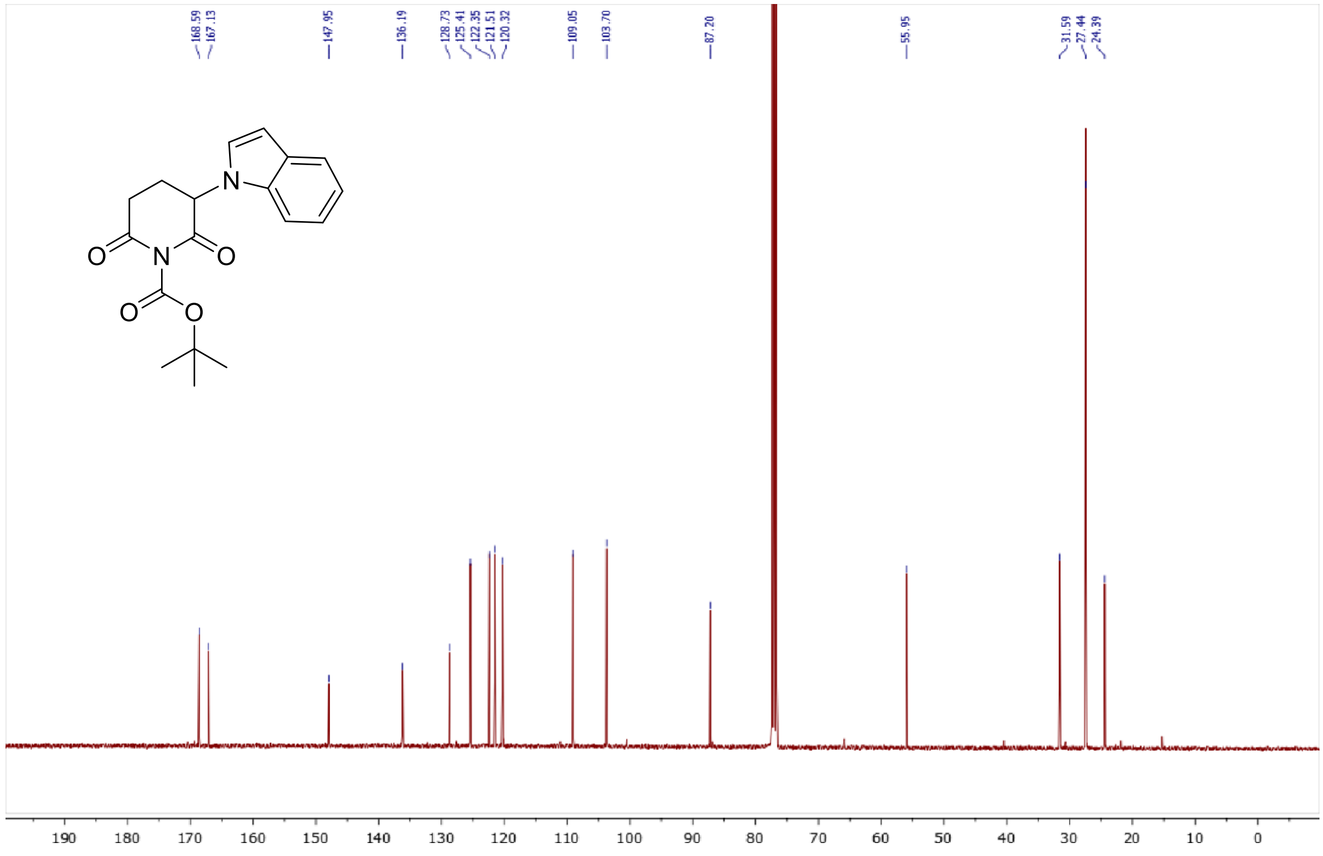

Copies of ^{1}H (400.13 MHz, CDCl$_3$) and ^{13}C{^{1}H} (100.61 MHz, CDCl$_3$) spectra of **9a**

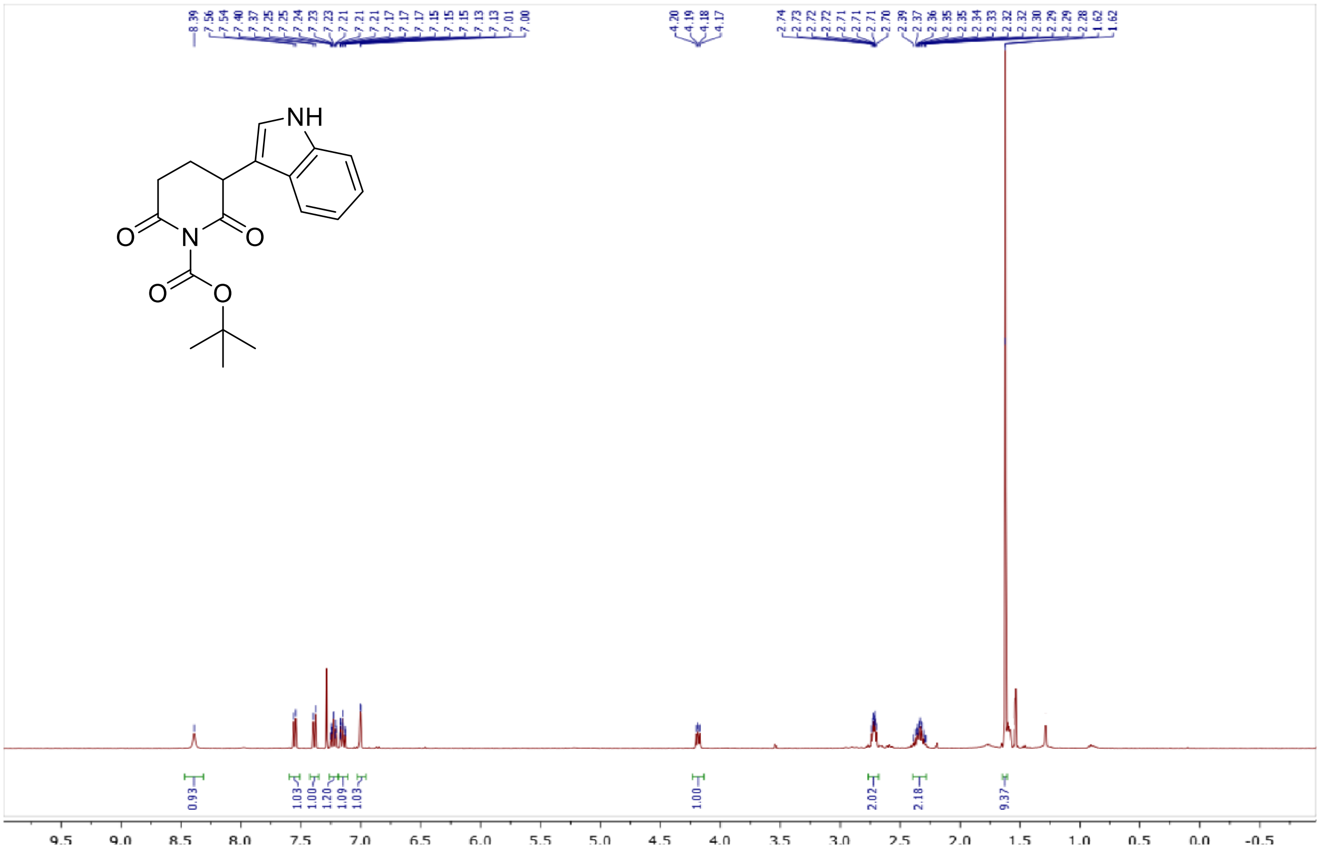

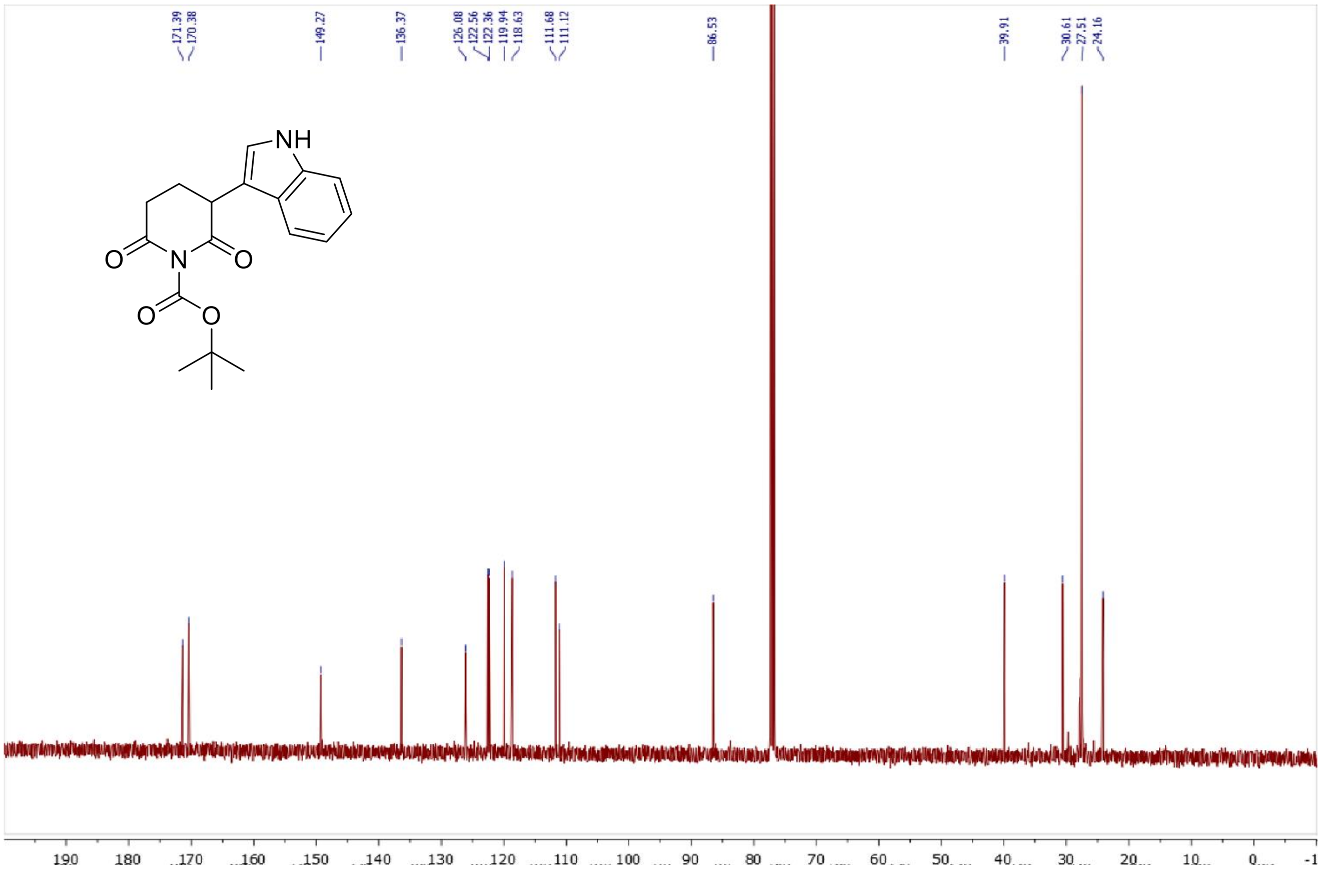

Copies of ^{1}H (400.13 MHz, $CDCl_3$) and $^{13}C\{^{1}H\}$ (100.61 MHz, $CDCl_3$) spectra of **6b**

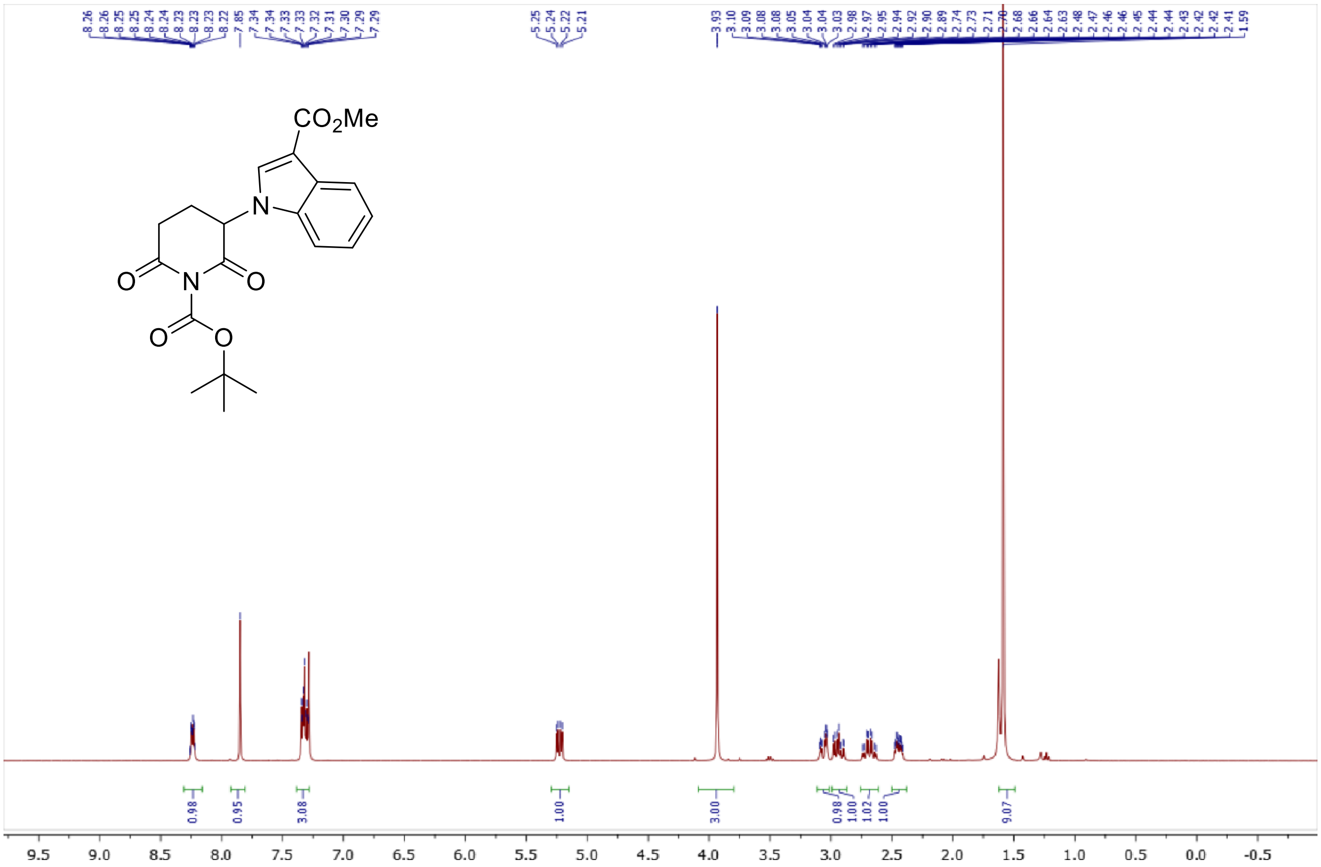

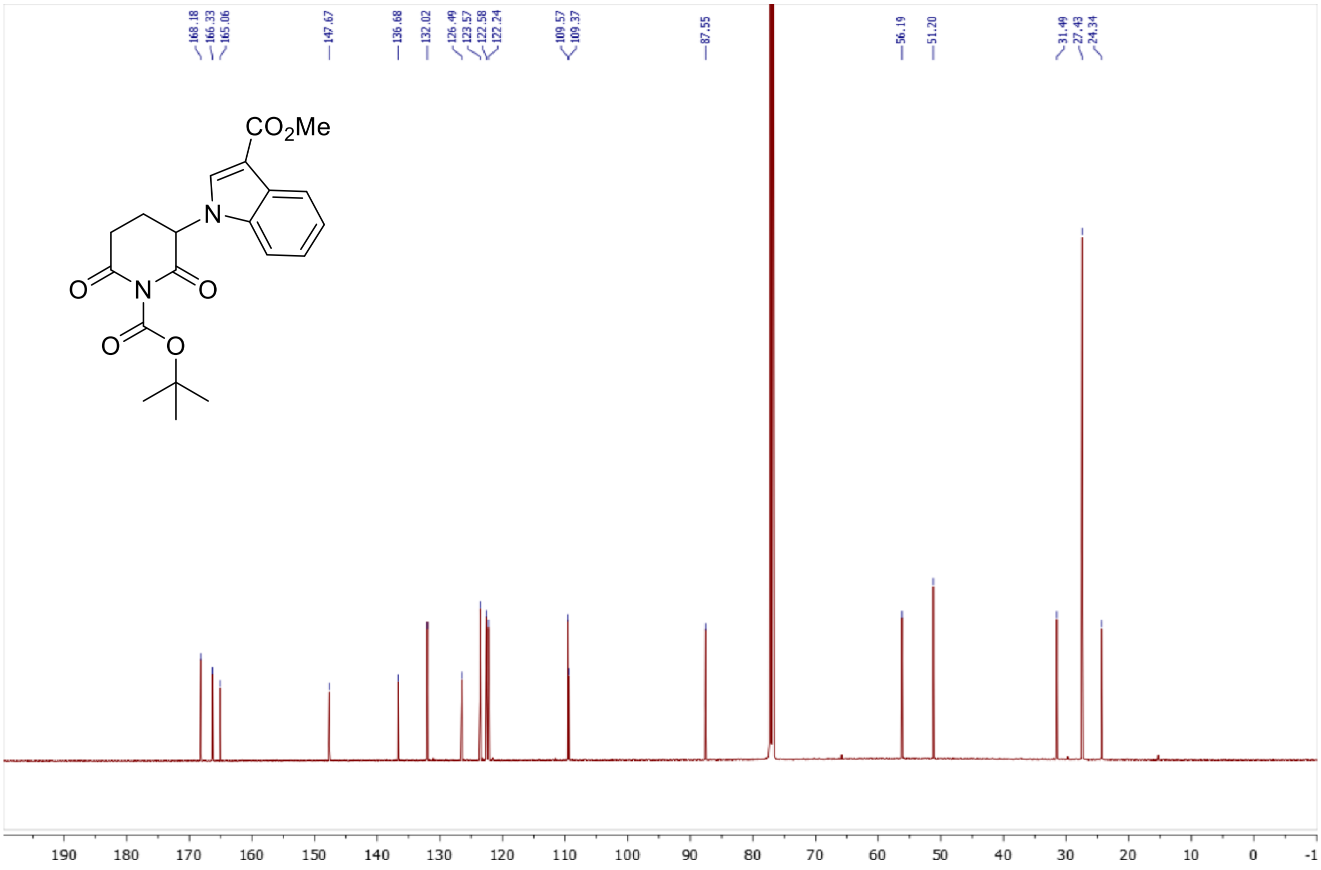

Copies of ^{1}H (400.13 MHz, $CDCl_3$) and $^{13}C\{^{1}H\}$ (100.61 MHz, $CDCl_3$) spectra of **9c**

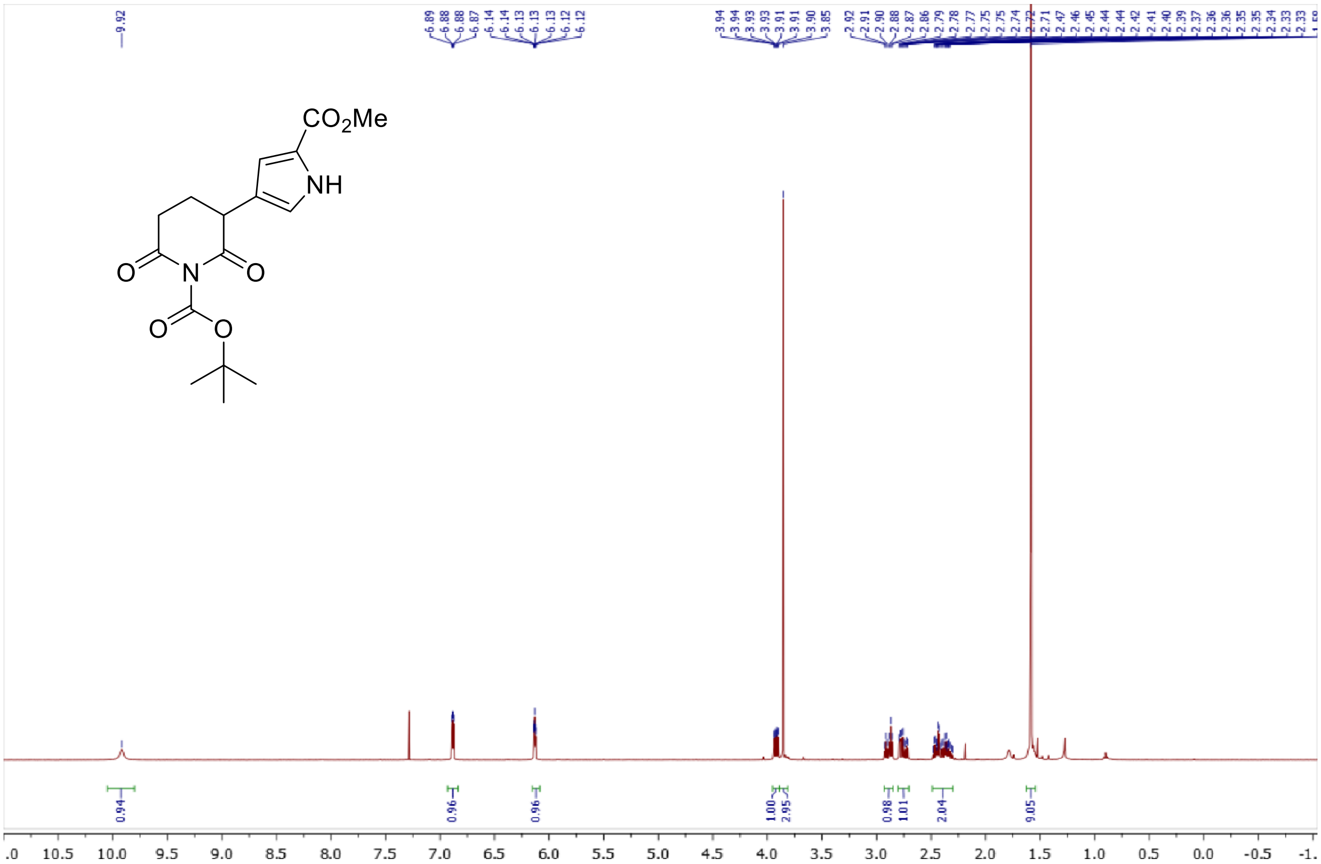

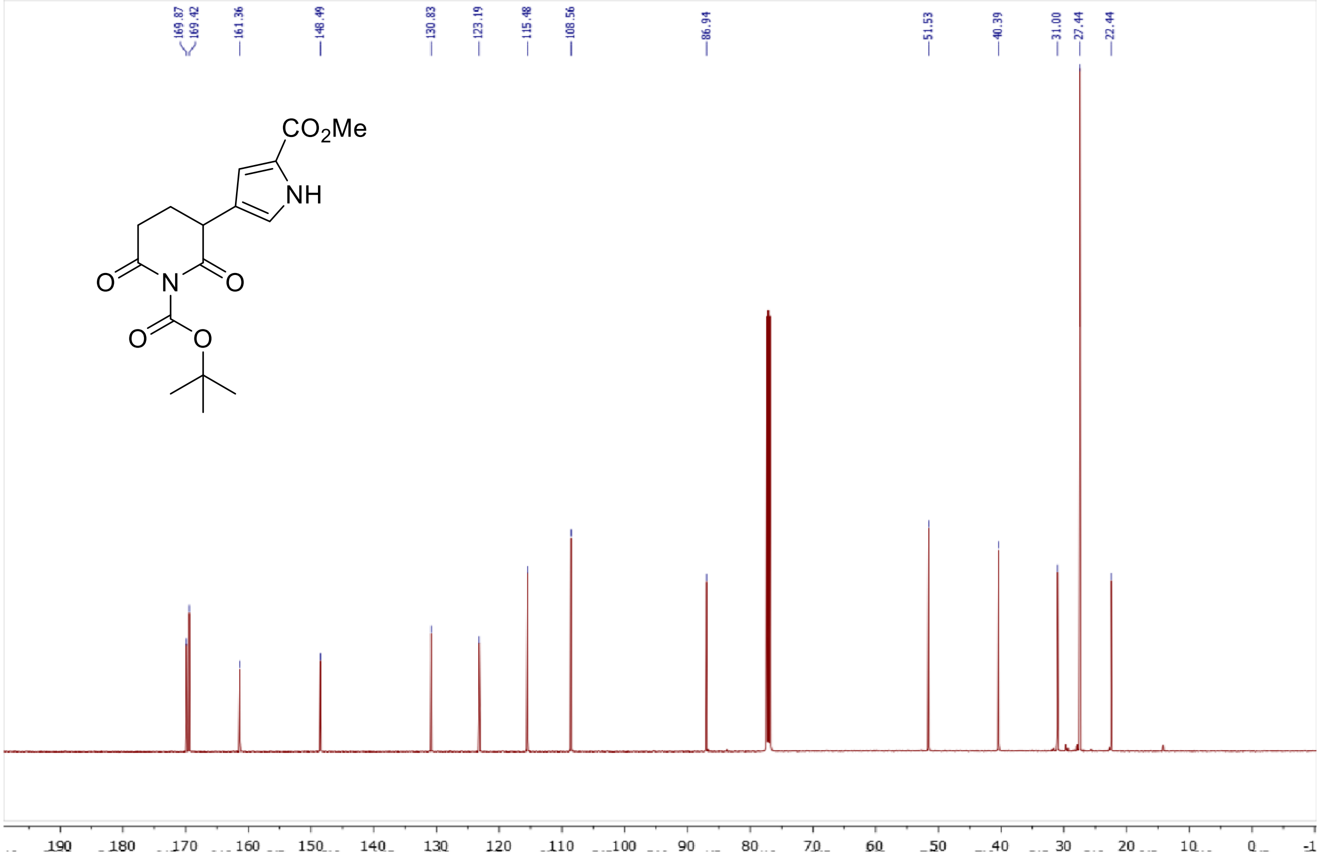

Copies of ^{1}H (400.13 MHz, $CDCl_3$) and $^{13}C\{^{1}H\}$ (100.61 MHz, $CDCl_3$) spectra of **6d**

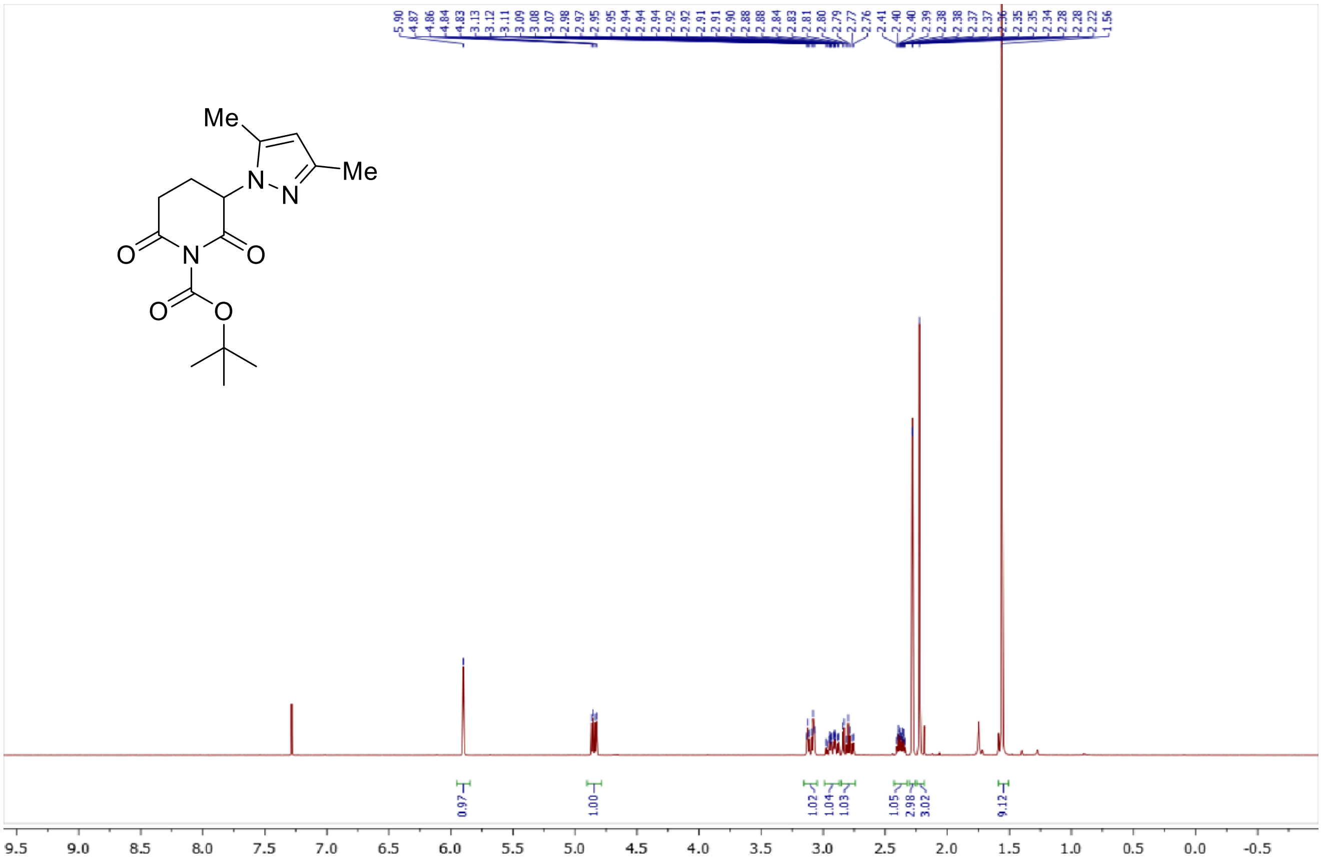

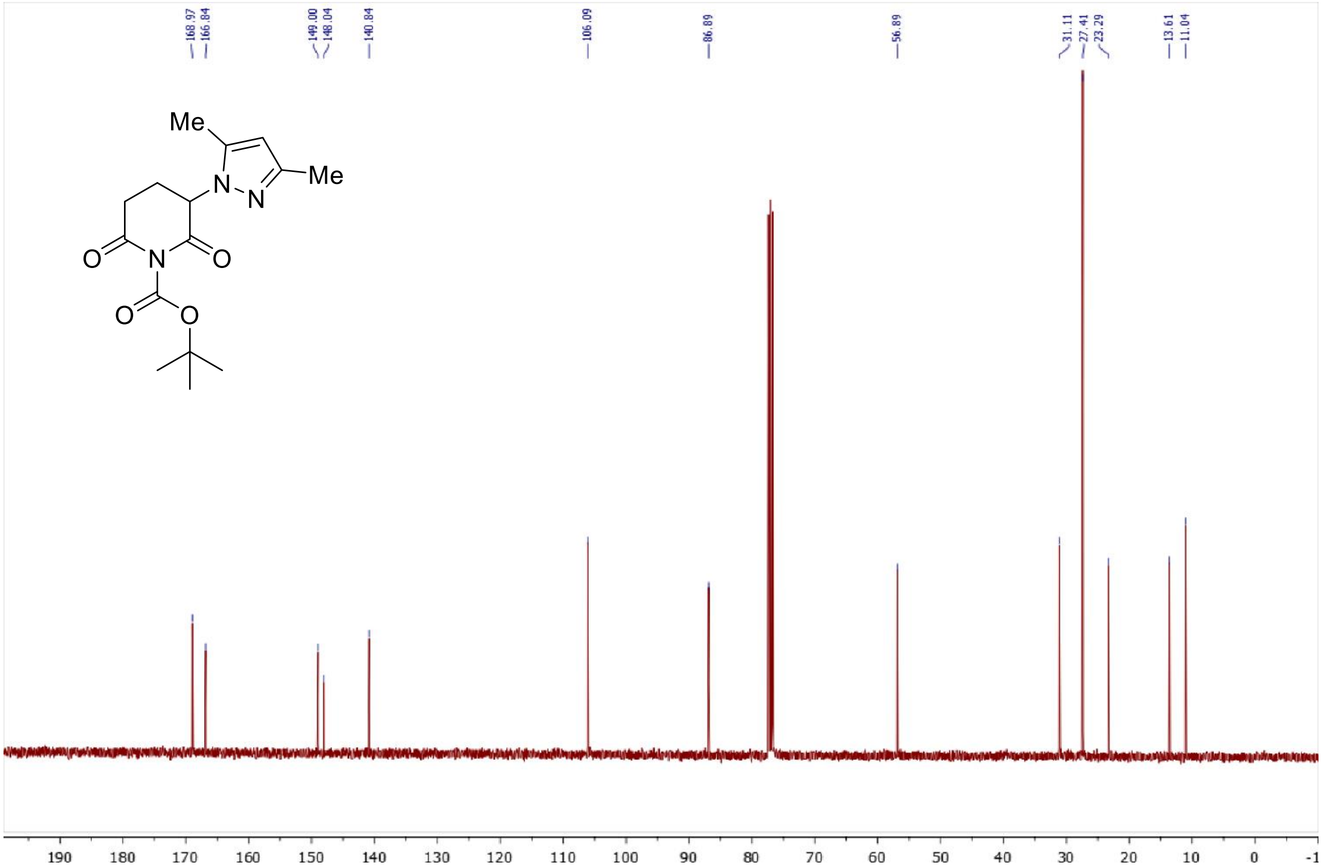

Copies of ^{1}H (400.13 MHz, $CDCl_3$) and $^{13}C\{^{1}H\}$ (100.61 MHz, $CDCl_3$) spectra of **6e**

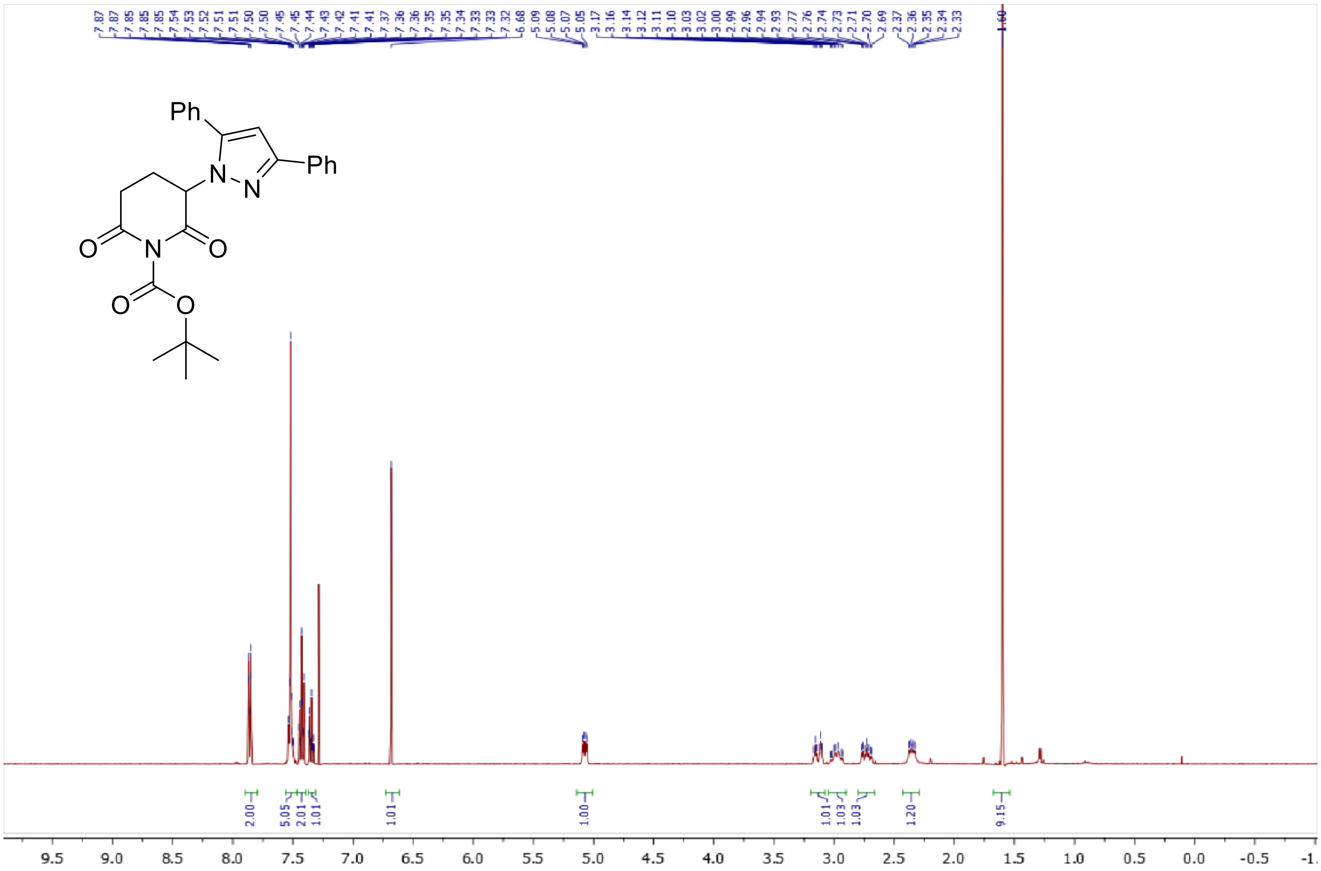

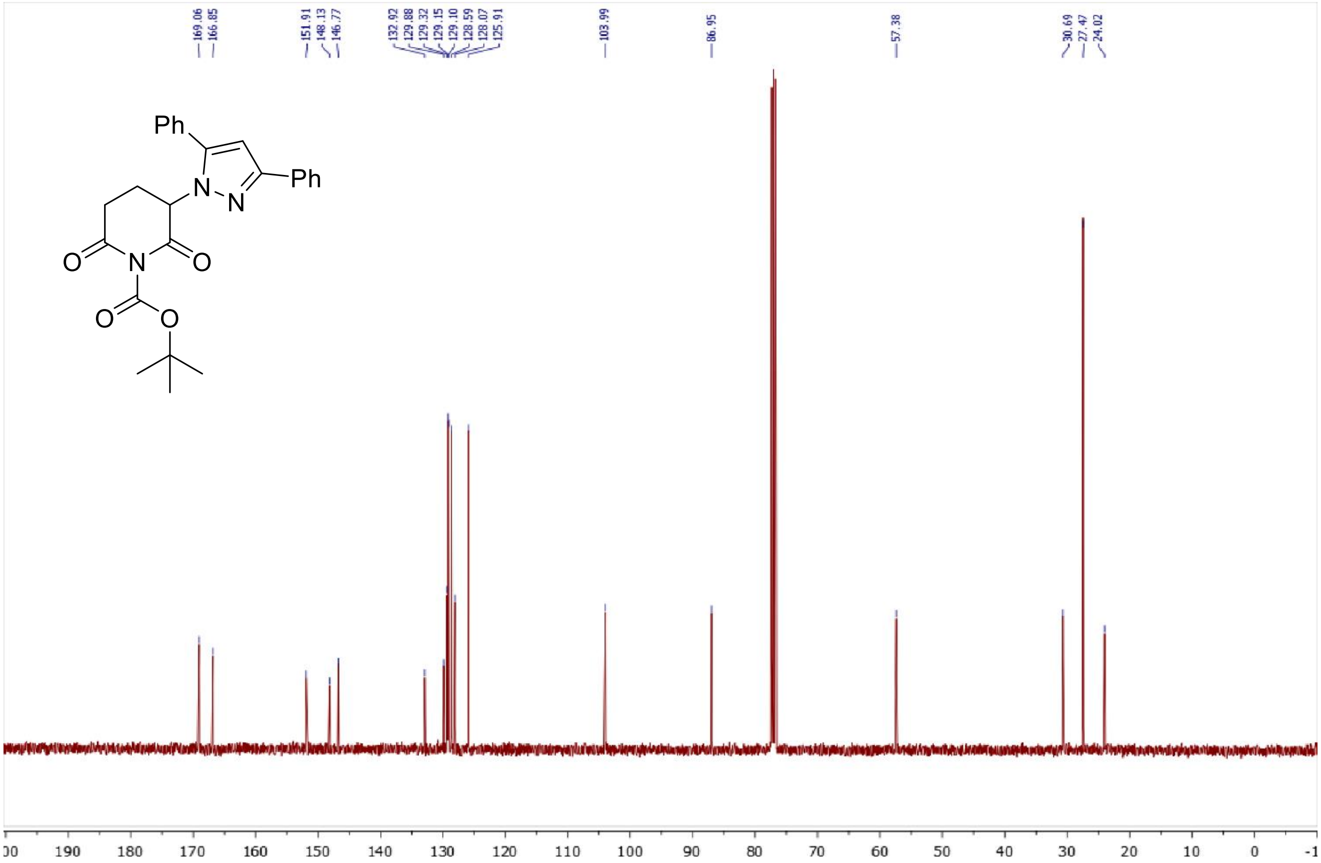

Copies of ^{1}H (400.13 MHz, $CDCl_3$) and $^{13}C\{^{1}H\}$ (100.61 MHz, $CDCl_3$) spectra of **6f**

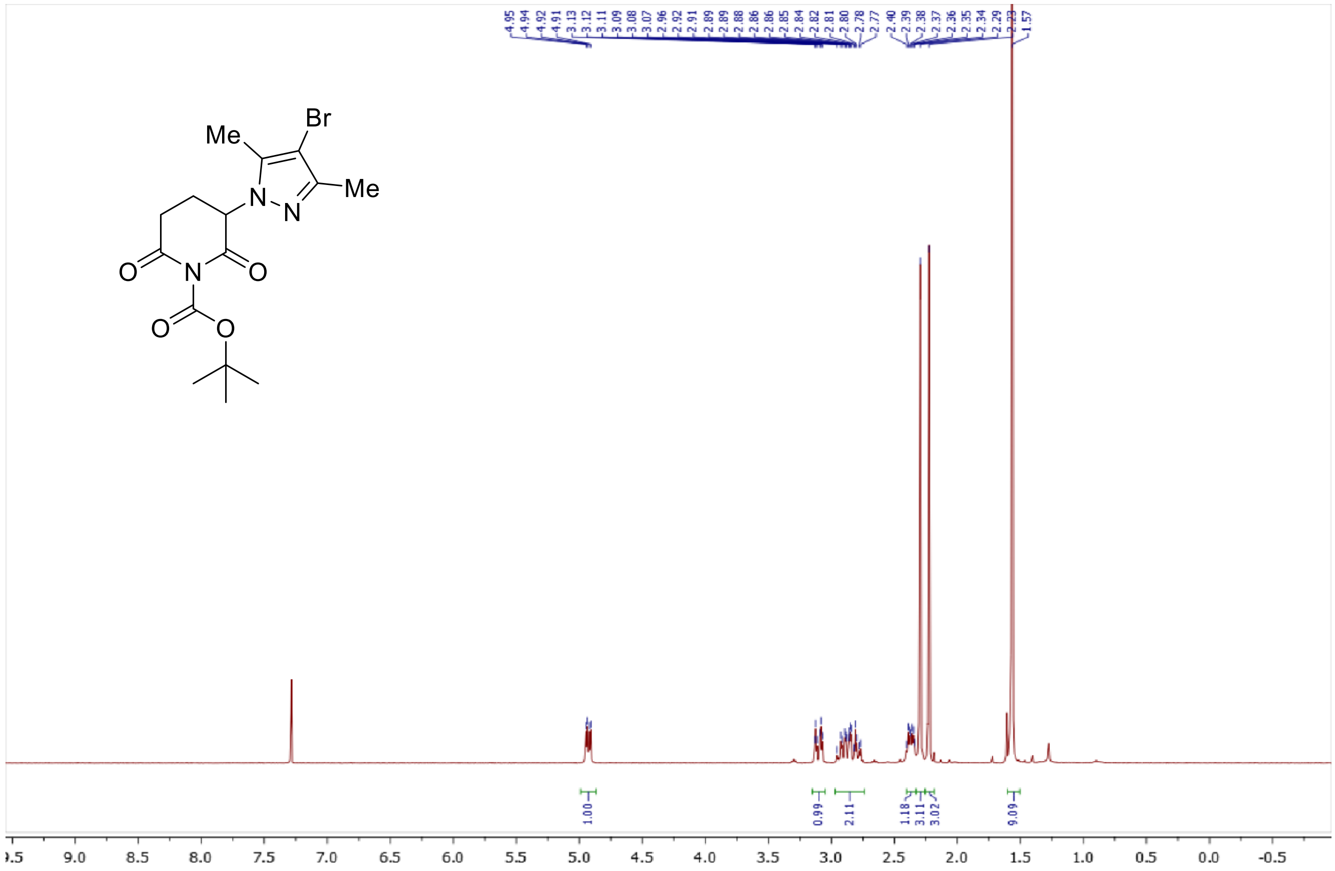

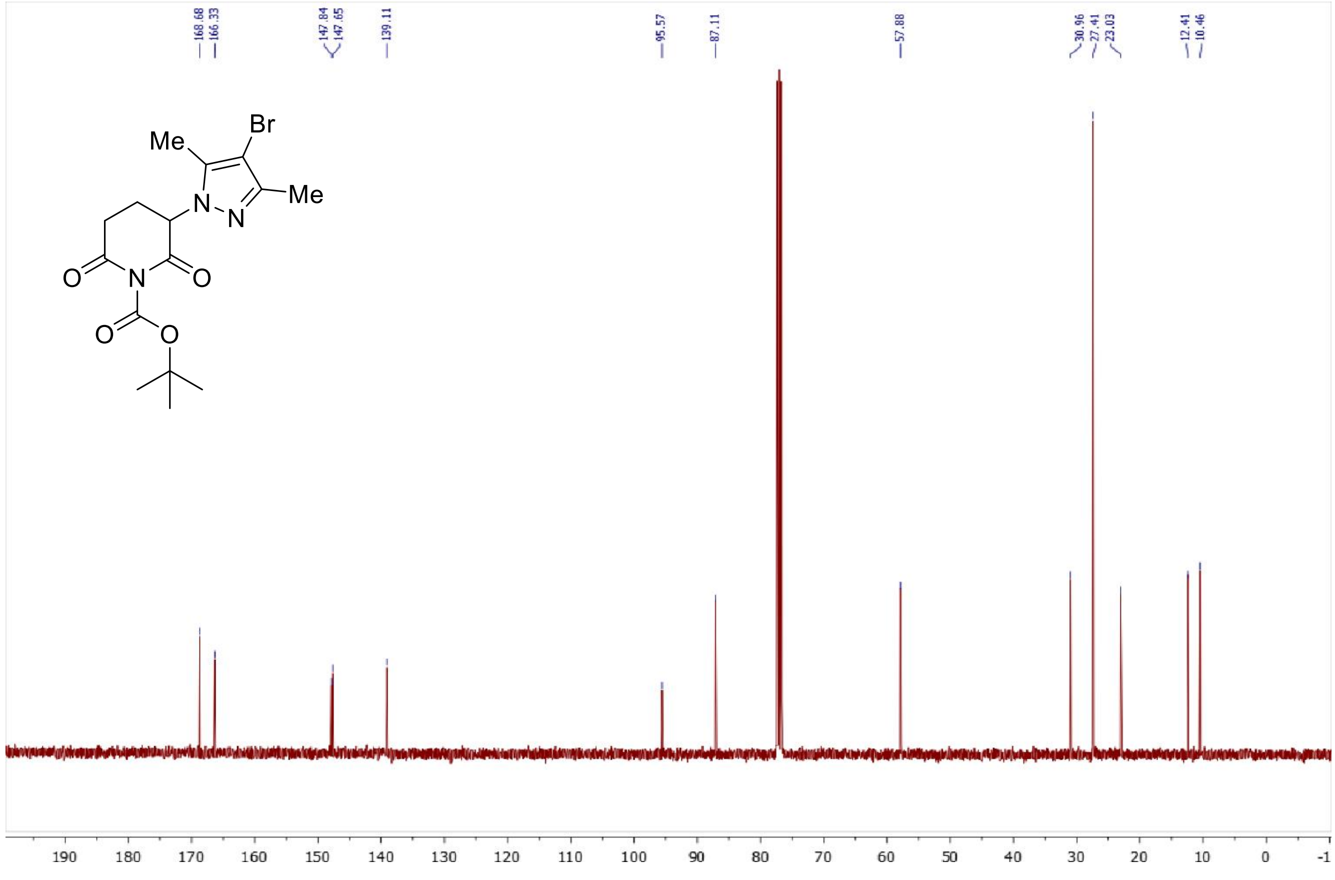

Copies of ^{1}H (400.13 MHz, $CDCl_3$) and $^{13}C\{^{1}H\}$ (100.61 MHz, $CDCl_3$) spectra of **6g**

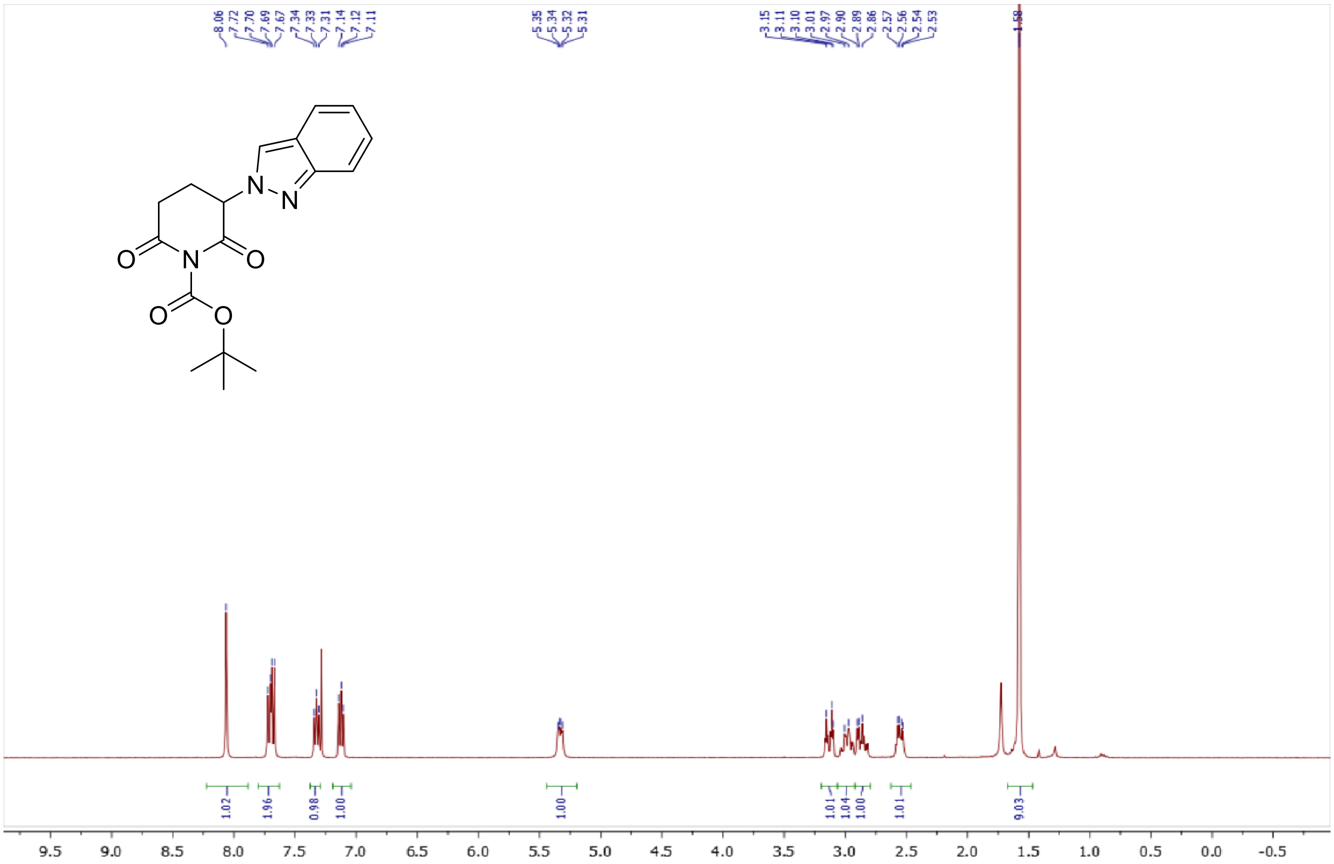

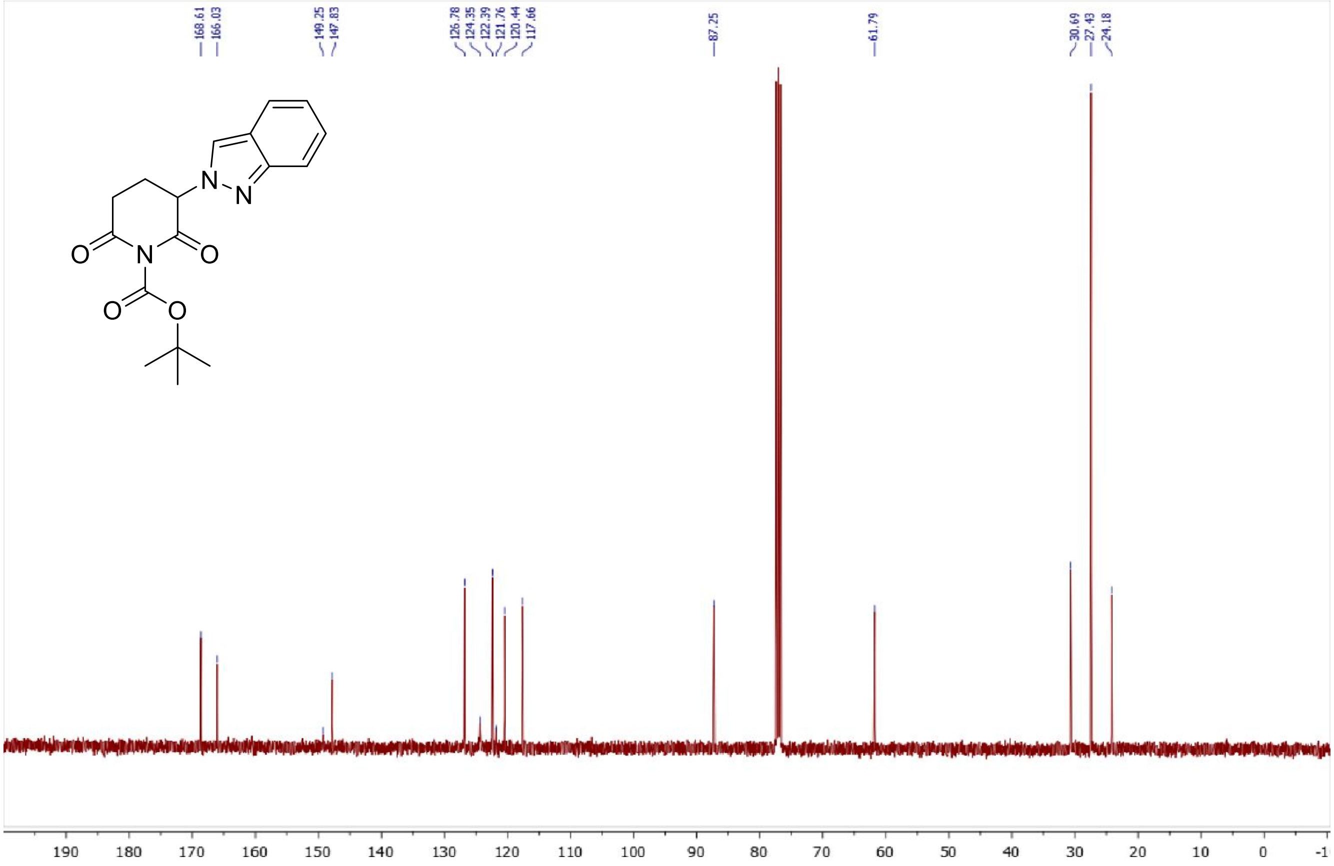

Copy of NOESY spectrum of **6g**

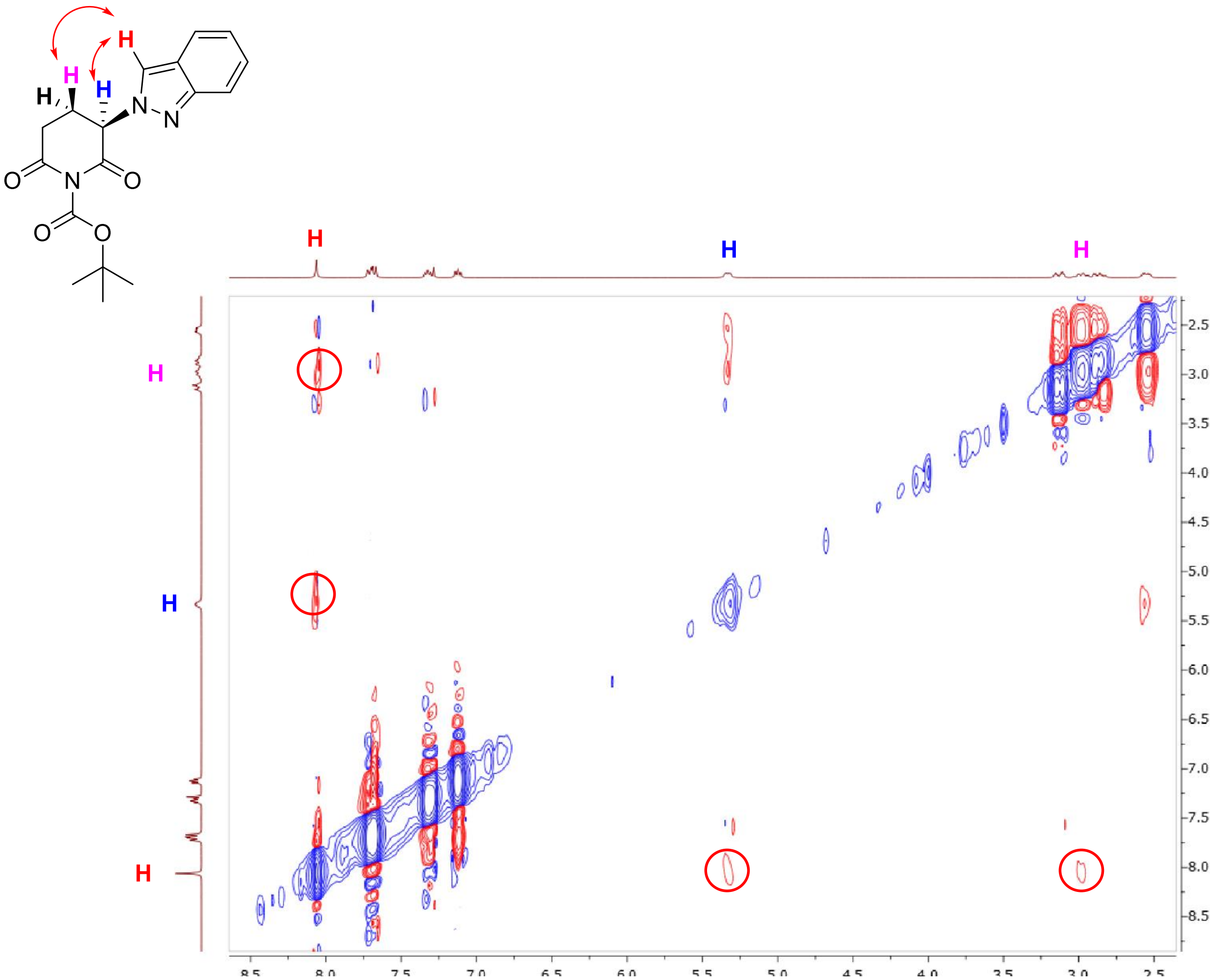

Copies of ^{1}H (400.13 MHz, CDCl$_3$) and ^{13}C{^{1}H} (100.61 MHz, CDCl$_3$) spectra of **6h**

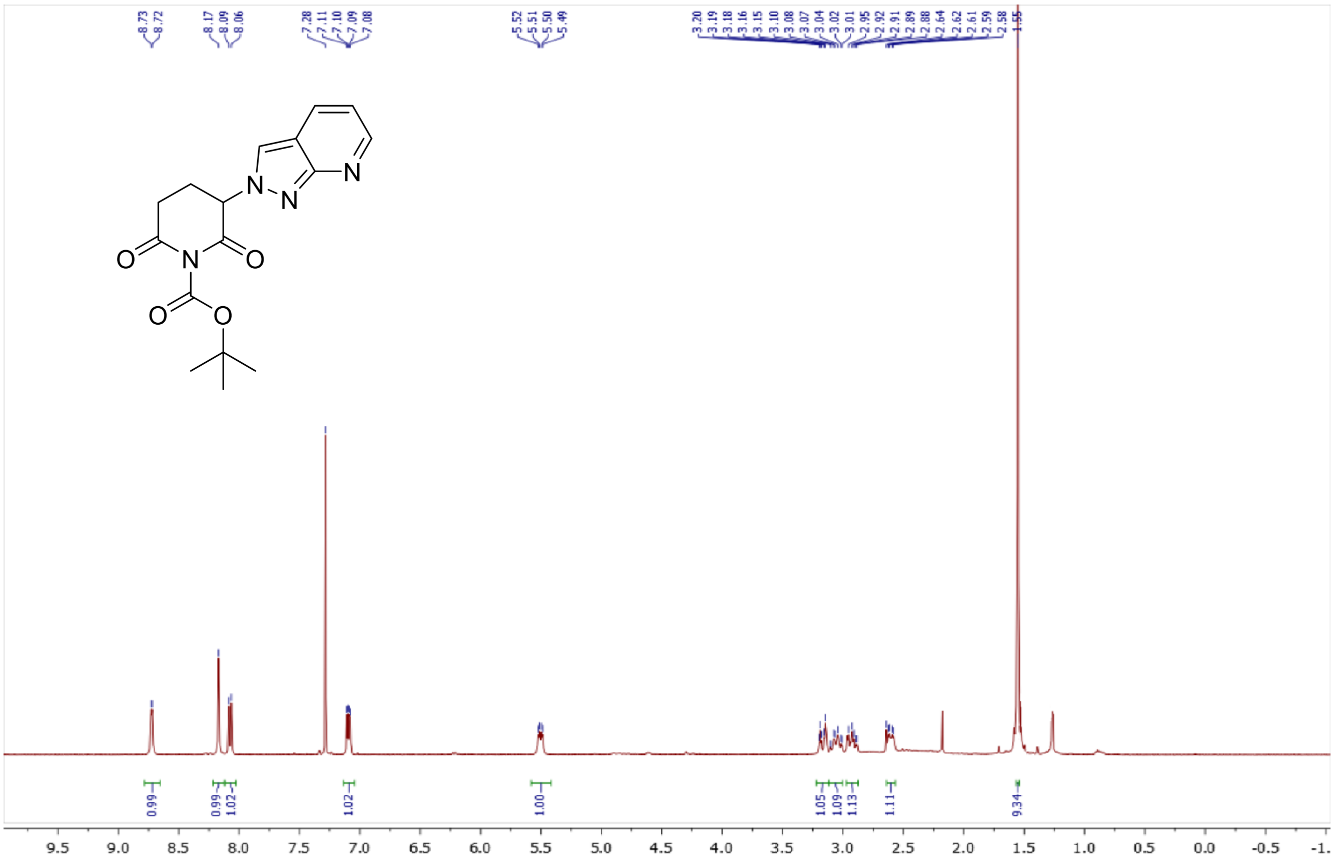

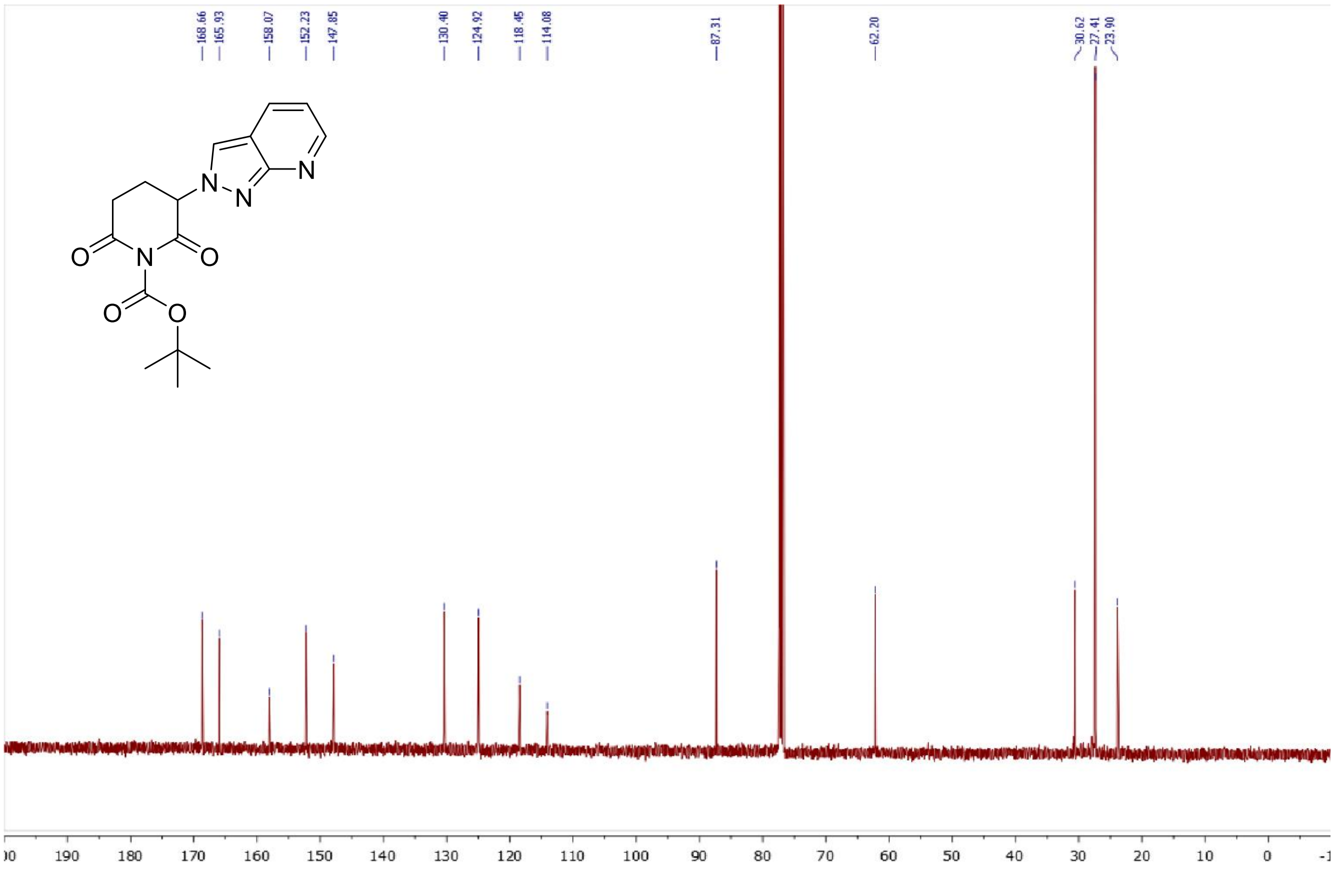

Copy of NOESY spectrum of **6h**

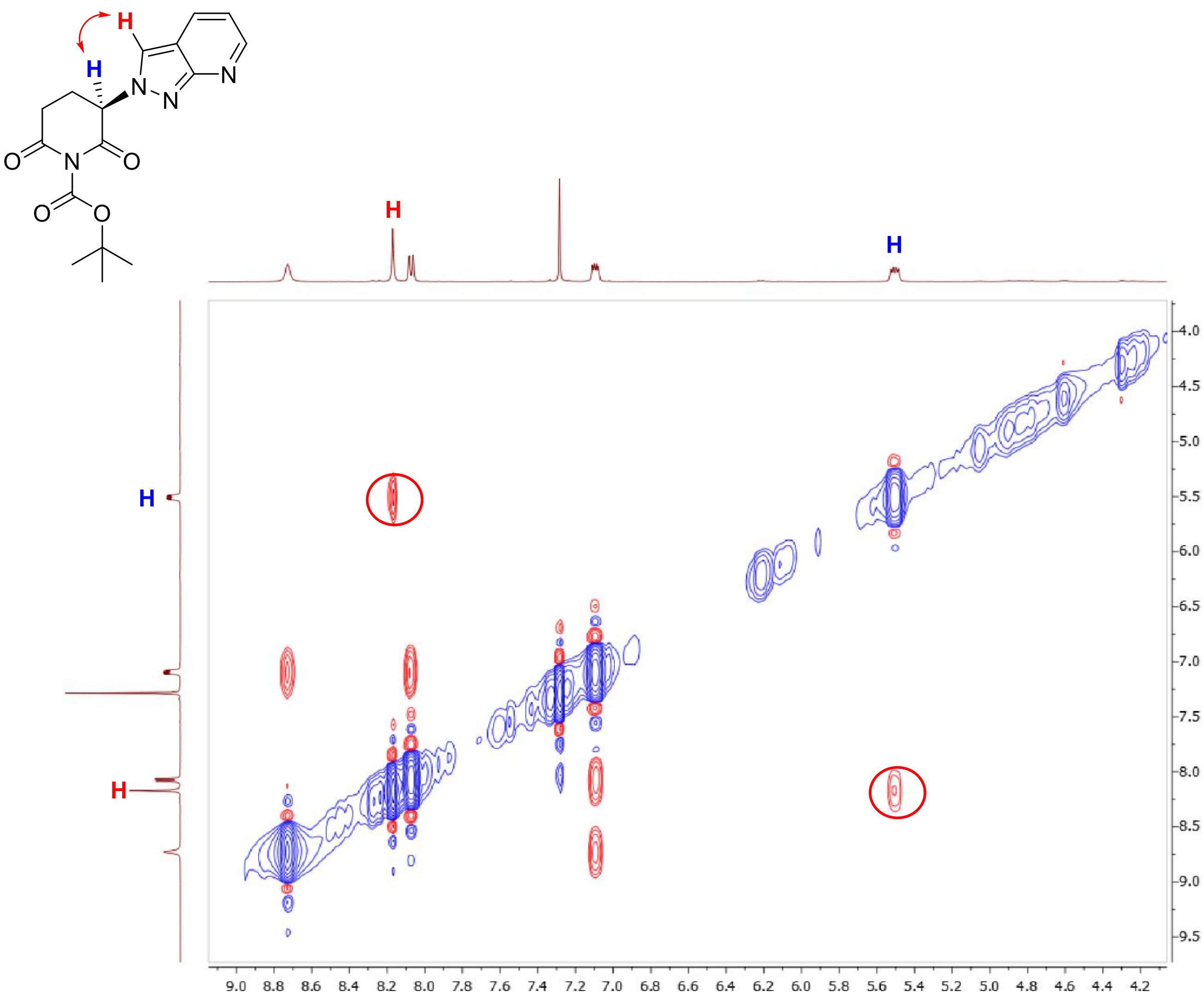

Copies of ^{1}H (400.13 MHz, $CDCl_3$) and $^{13}C\{^{1}H\}$ (100.61 MHz, $CDCl_3$) spectra of **9i**

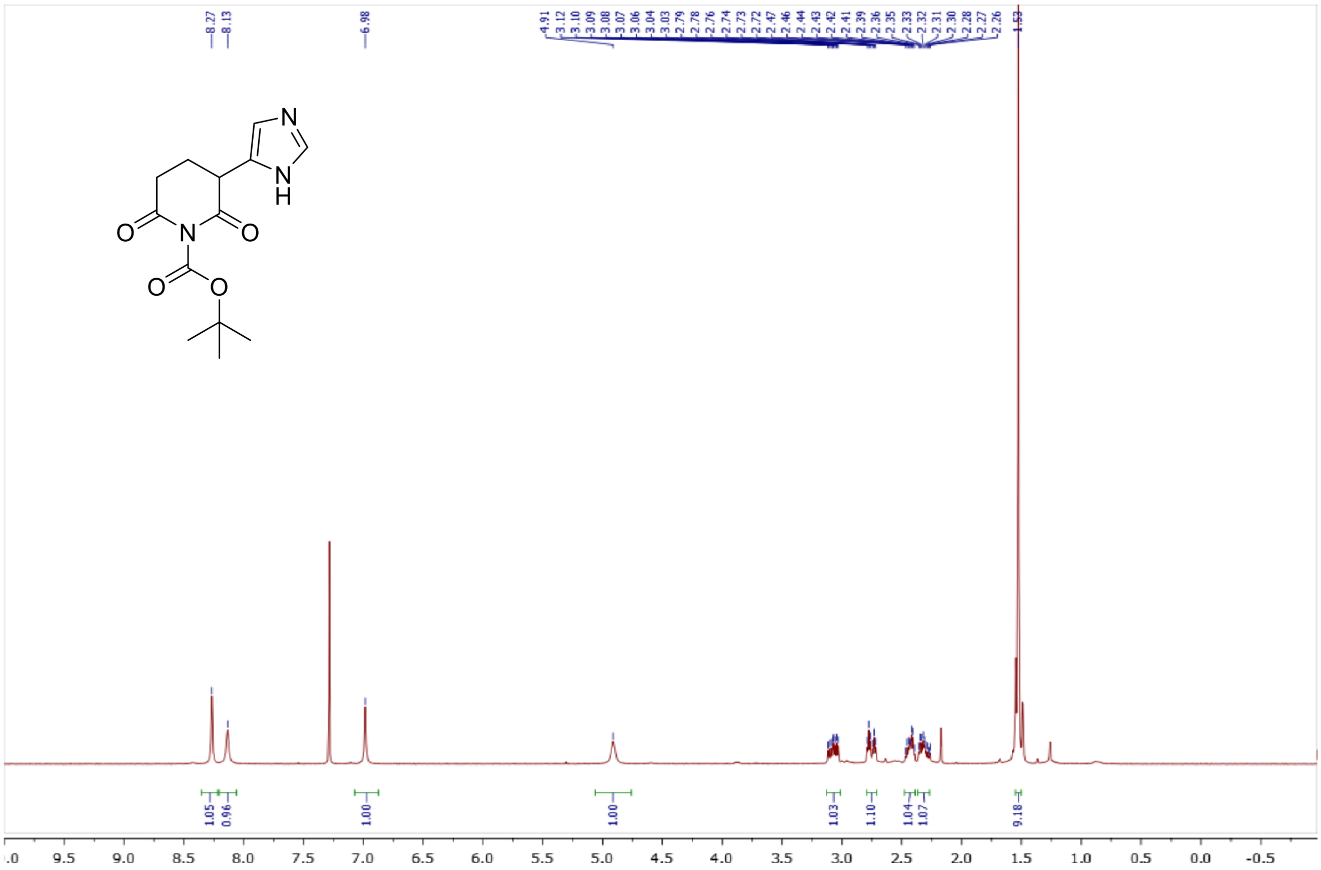

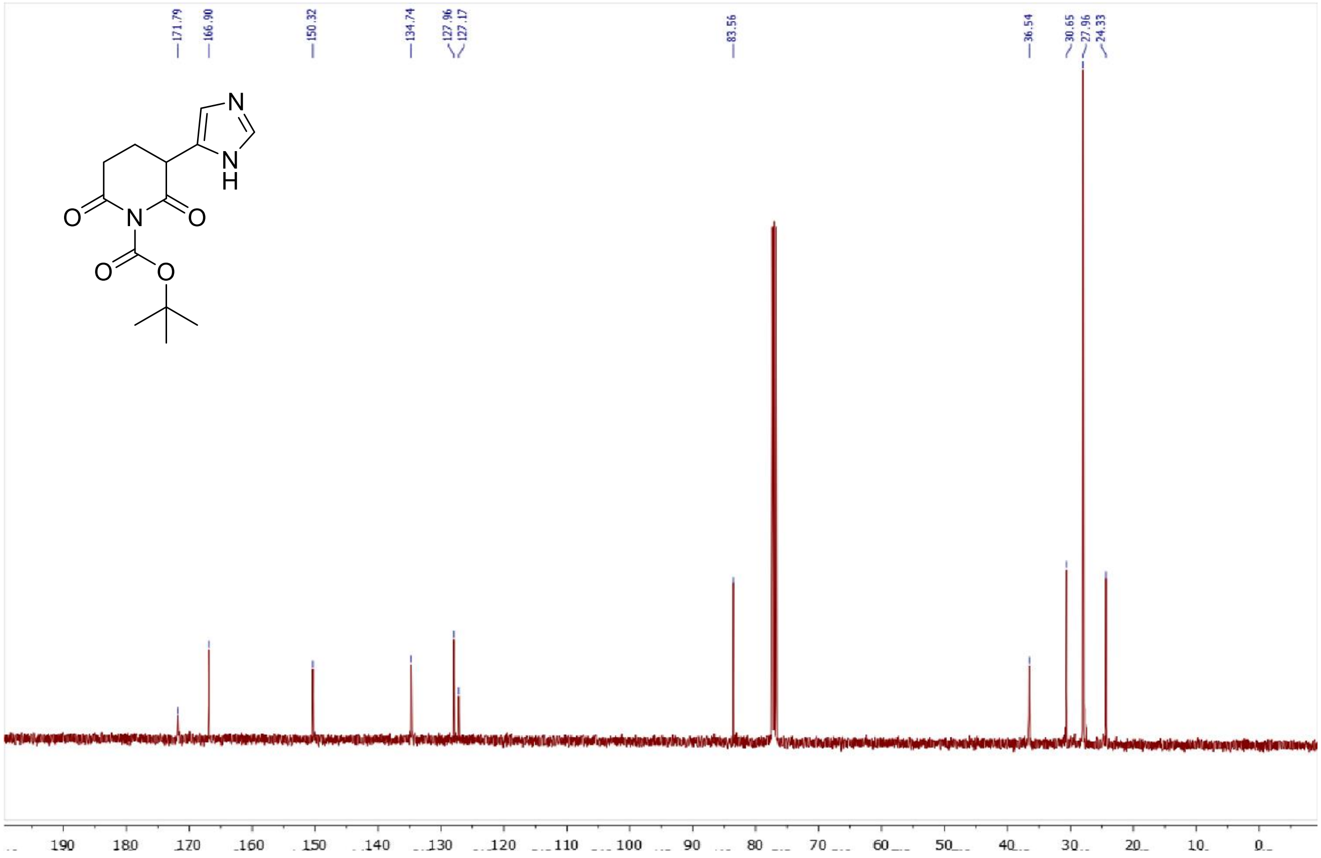

Copies of ^{1}H (400.13 MHz, $CDCl_3$) and $^{13}C\{^{1}H\}$ (100.61 MHz, $CDCl_3$) spectra of **6j**

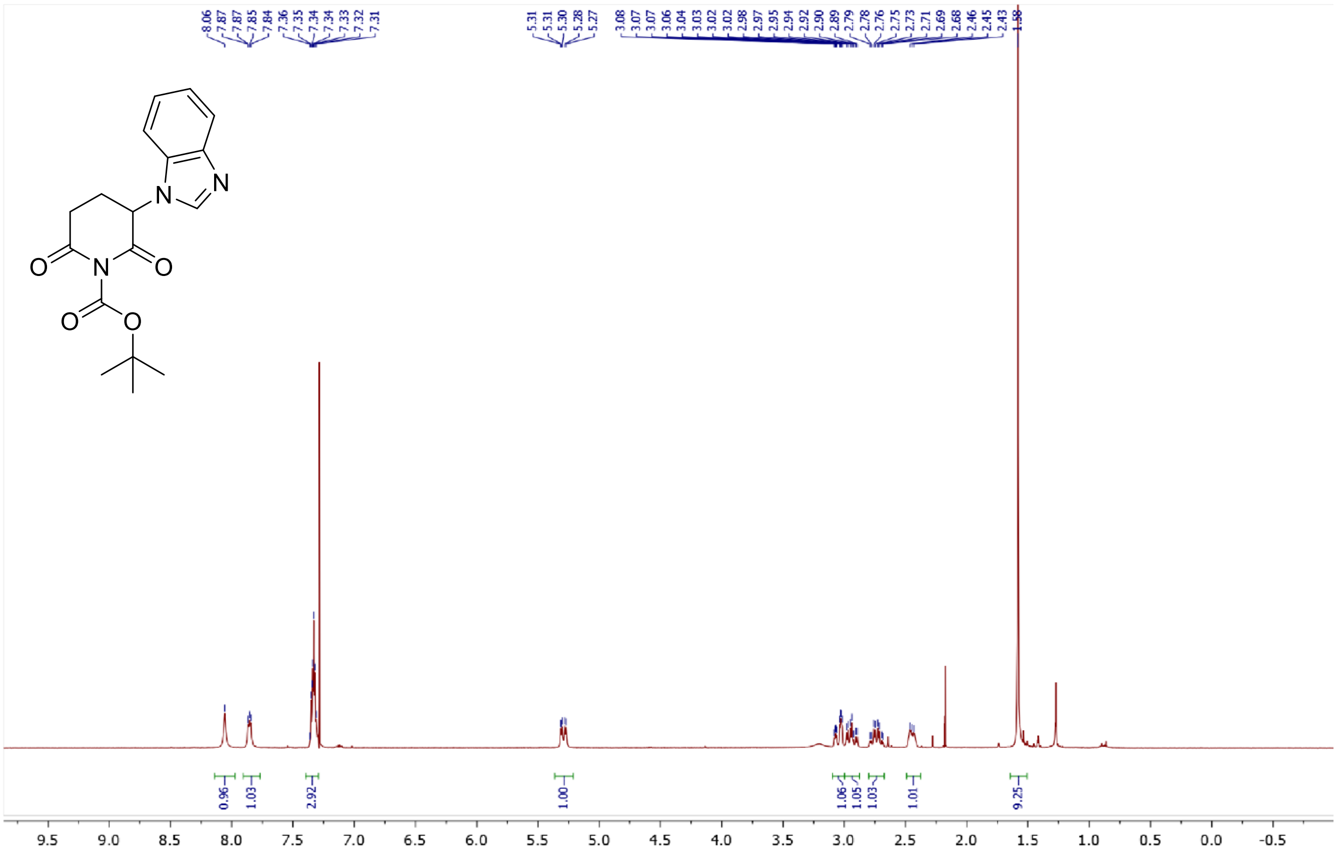

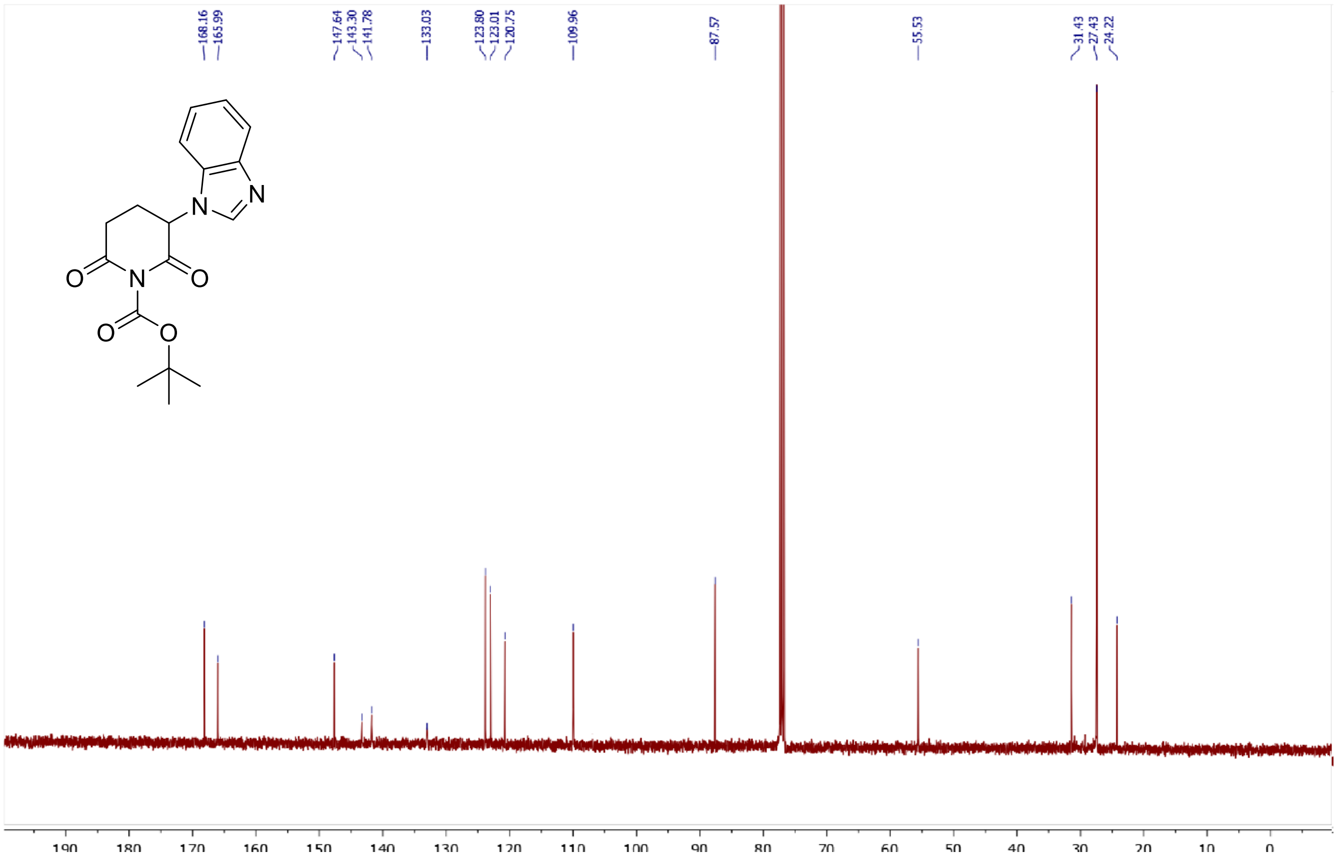

Copies of ^{1}H (400.13 MHz, $CDCl_3$) and $^{13}C\{^{1}H\}$ (100.61 MHz, $CDCl_3$) spectra of **6l**

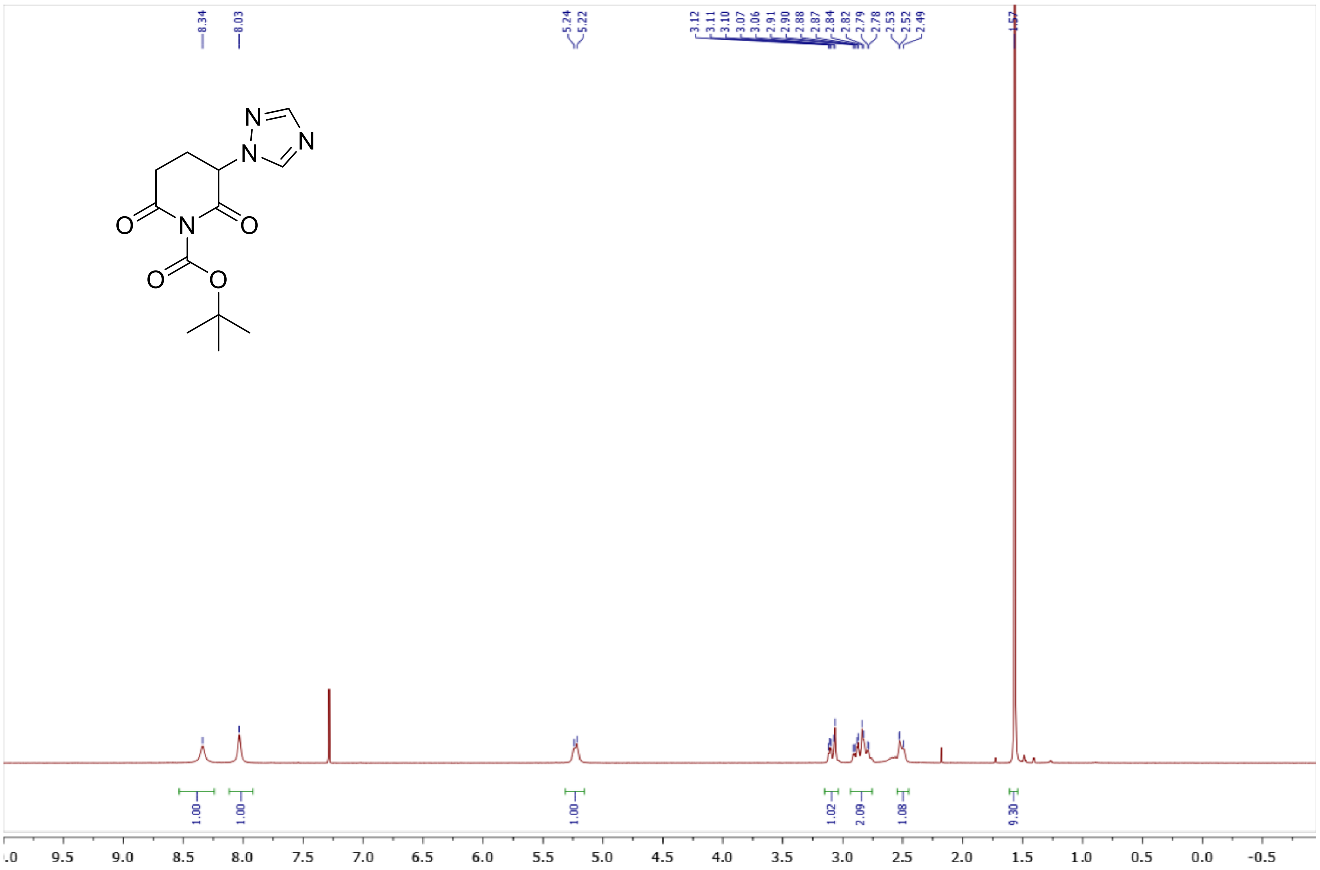

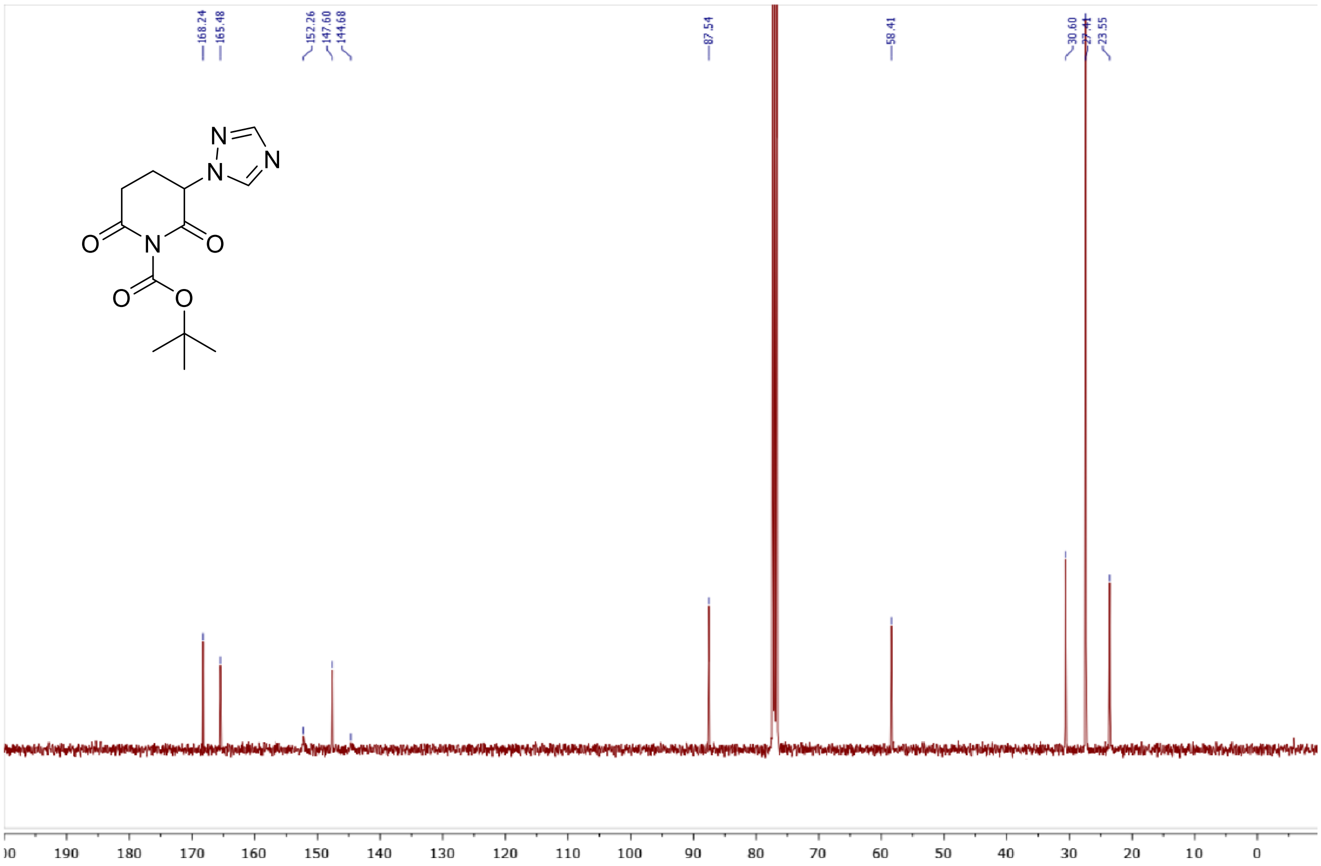

Copies of ^{1}H (400.13 MHz, $CDCl_3$) and $^{13}C\{^{1}H\}$ (100.61 MHz, $CDCl_3$) spectra of **6m**

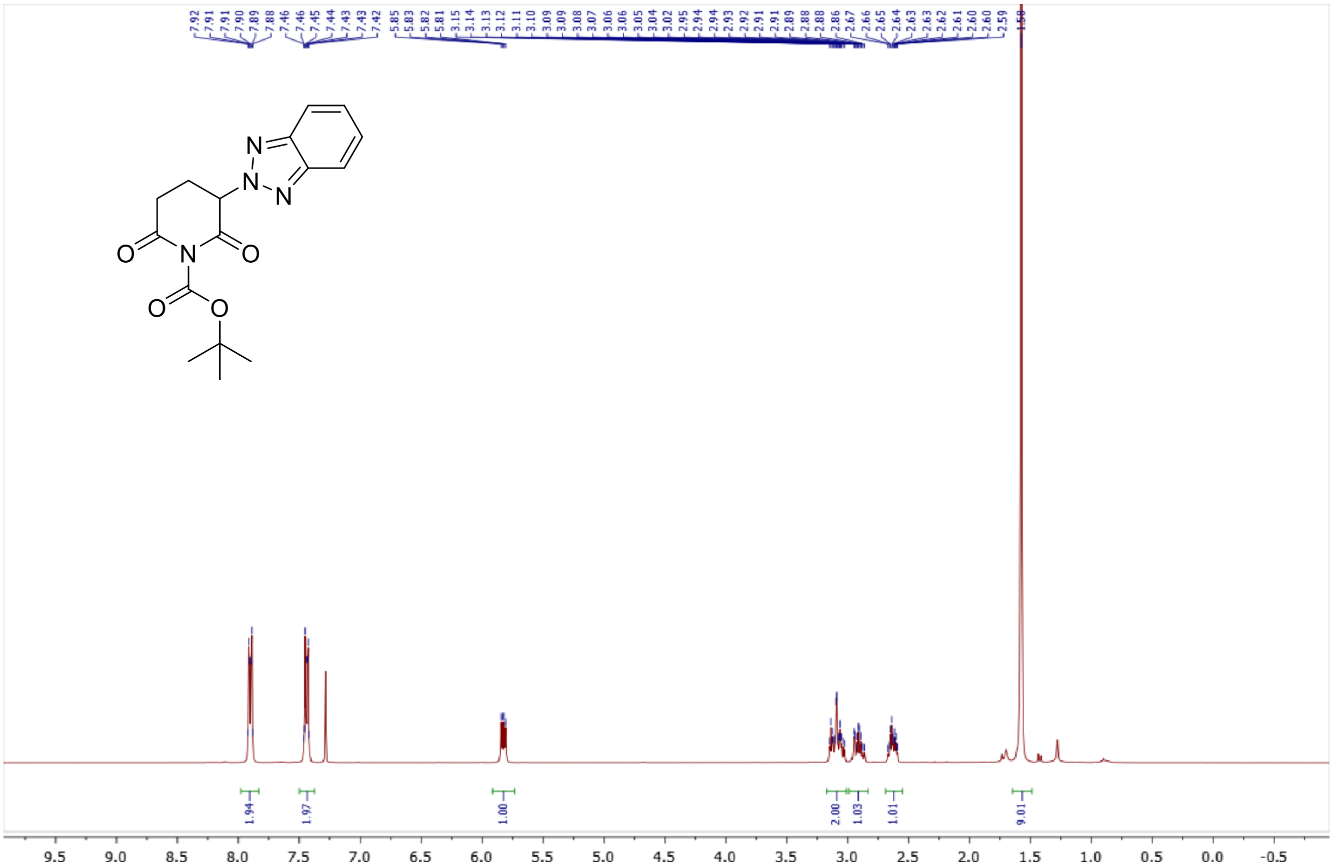

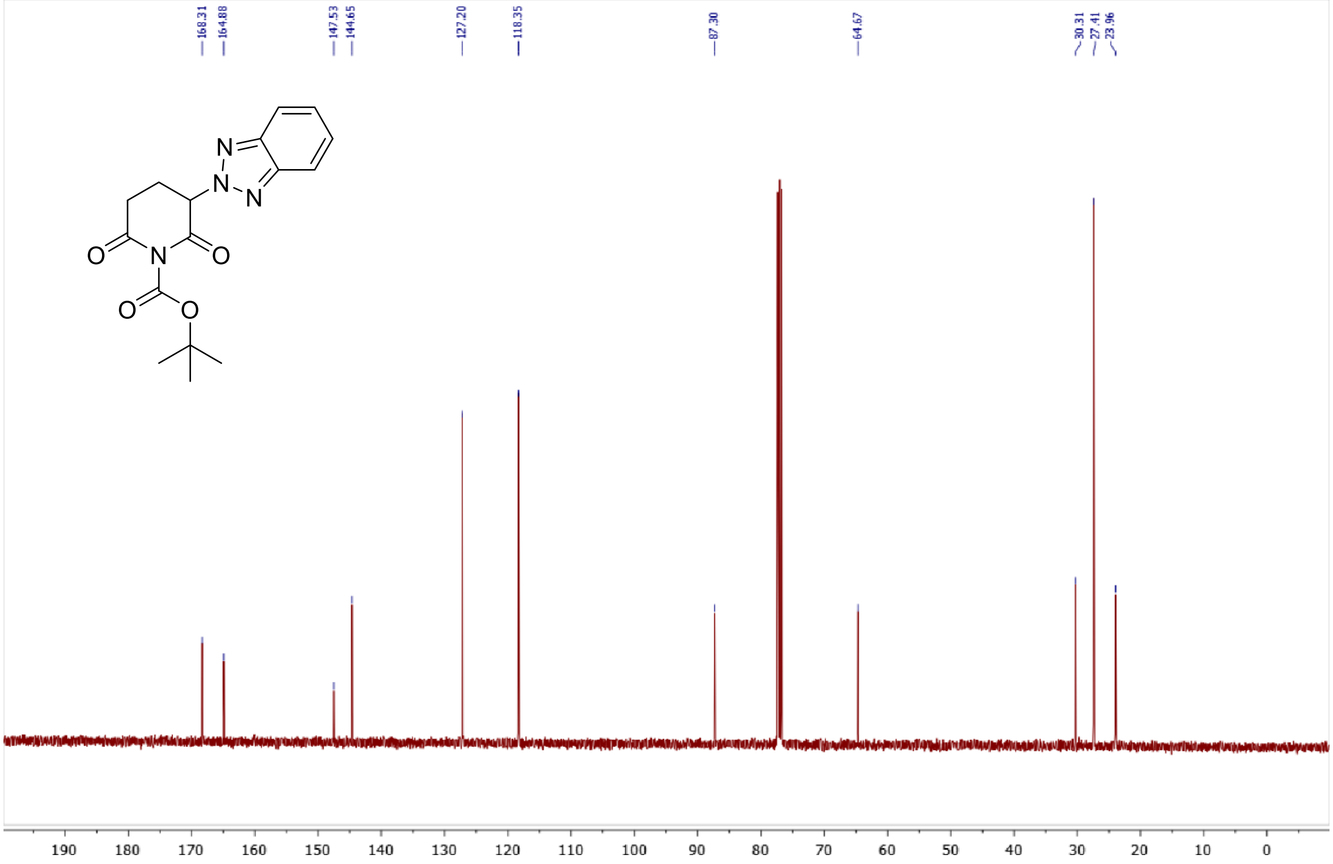

Copies of ^{1}H (400.13 MHz, $CDCl_3$) and $^{13}C\{^{1}H\}$ (100.61 MHz, $CDCl_3$) spectra of **6n**

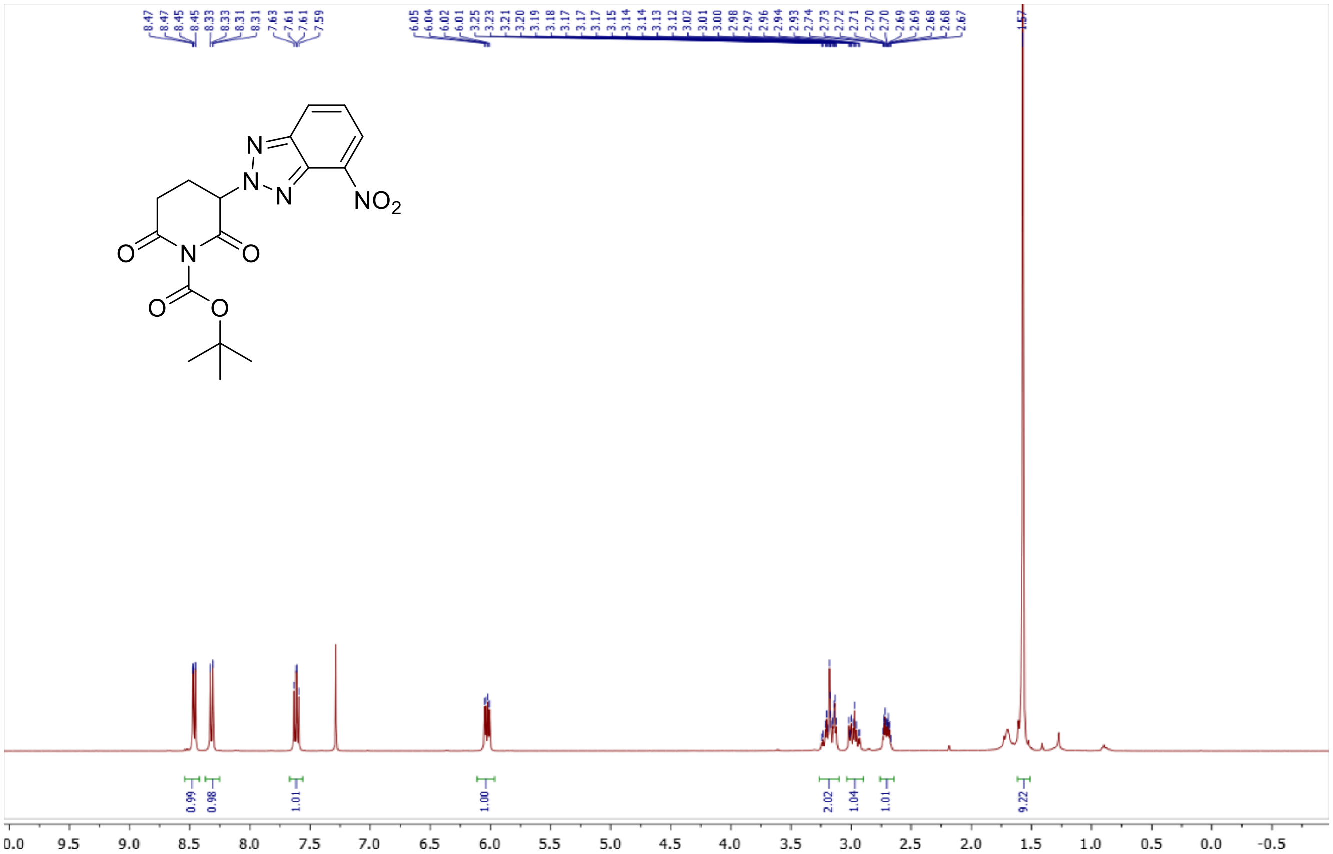

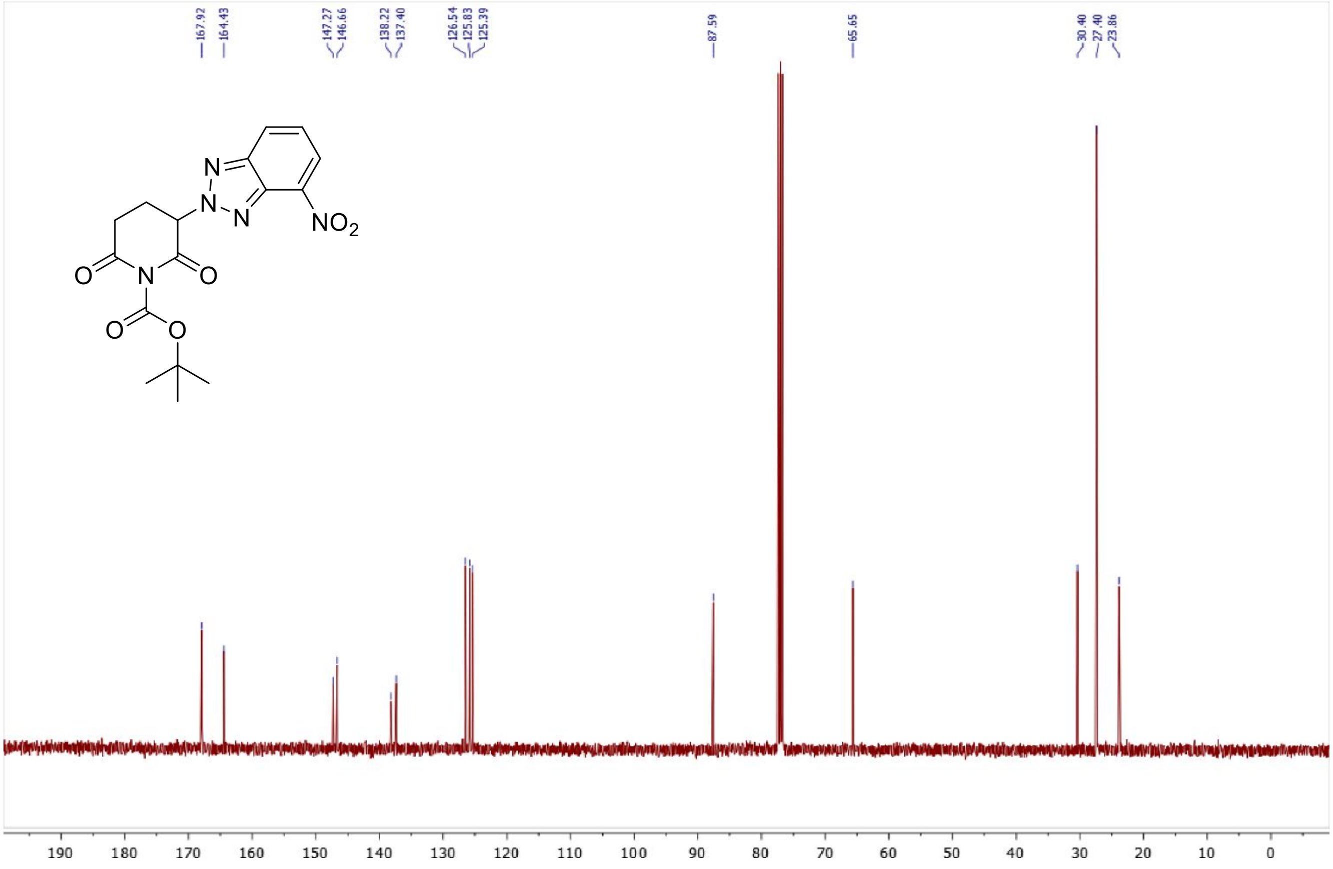

Copies of ^{1}H (400.13 MHz, $CDCl_3$) and $^{13}C\{^{1}H\}$ (100.61 MHz, $CDCl_3$) spectra of **6o**

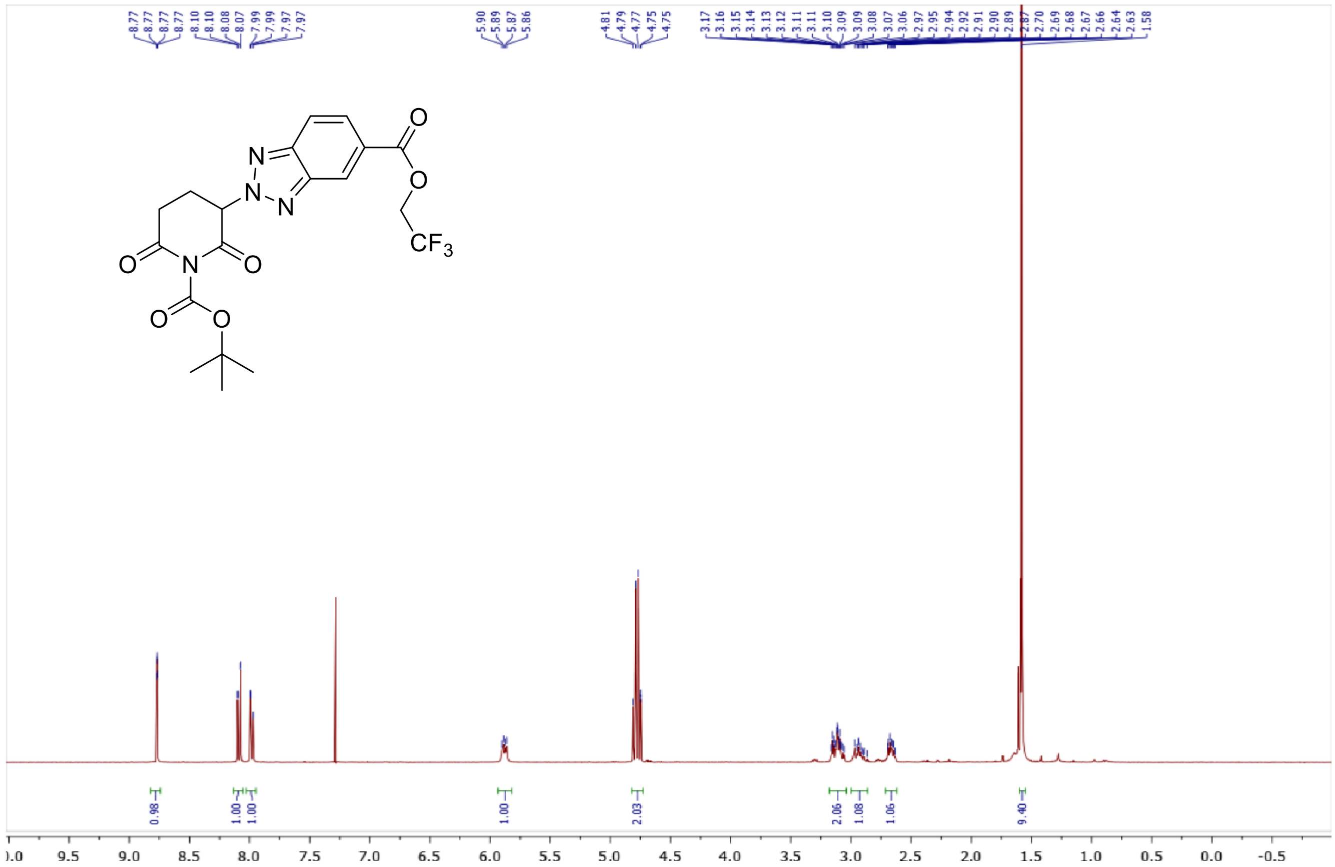

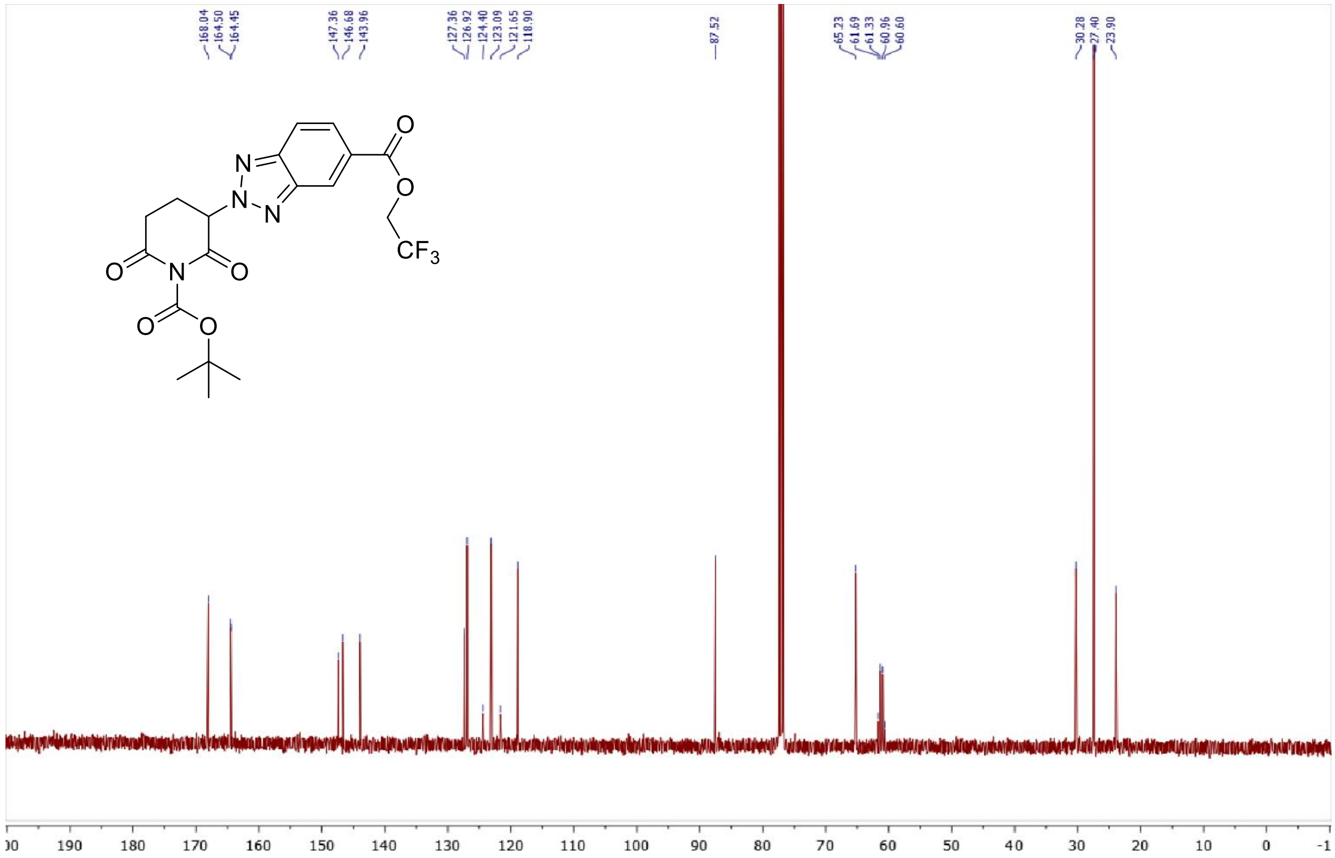

Copy of $^{19}F\{^{1}H\}$ (376.50 MHz, $CDCl_3$) spectrum of **6o**

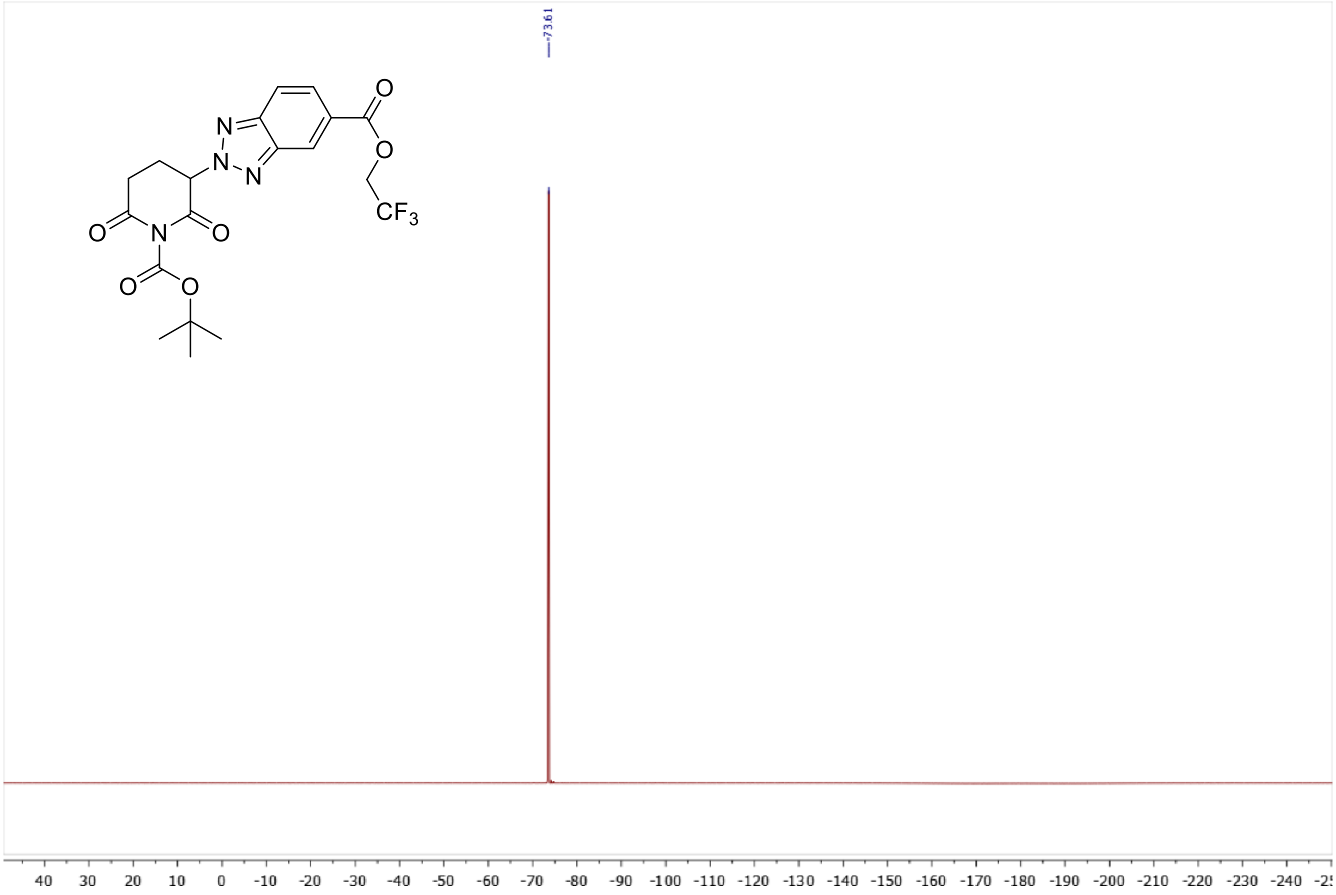

Copies of ^{1}H (400.13 MHz, $CDCl_3$) and $^{13}C\{^{1}H\}$ (100.61 MHz, $CDCl_3$) spectra of **6p**

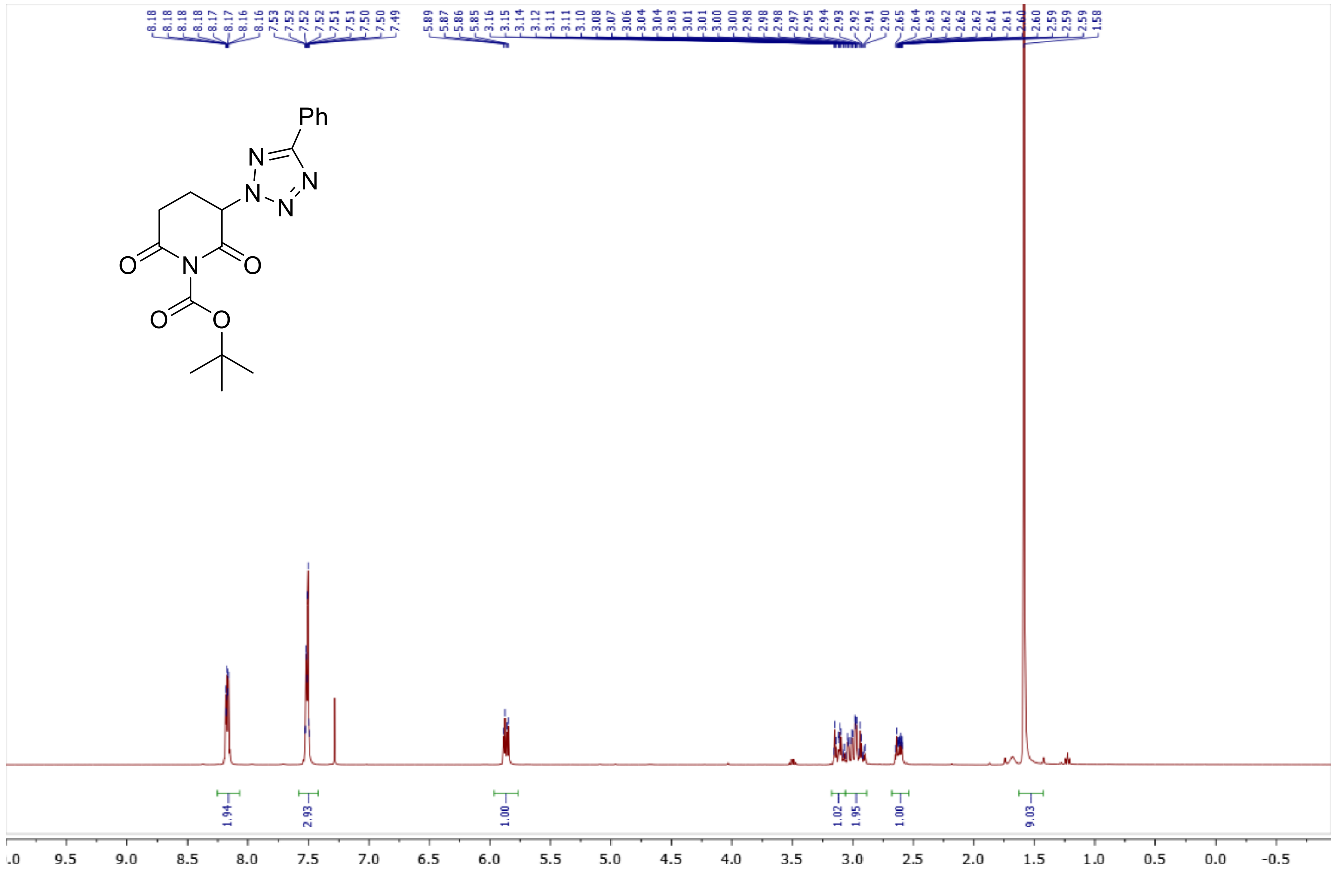

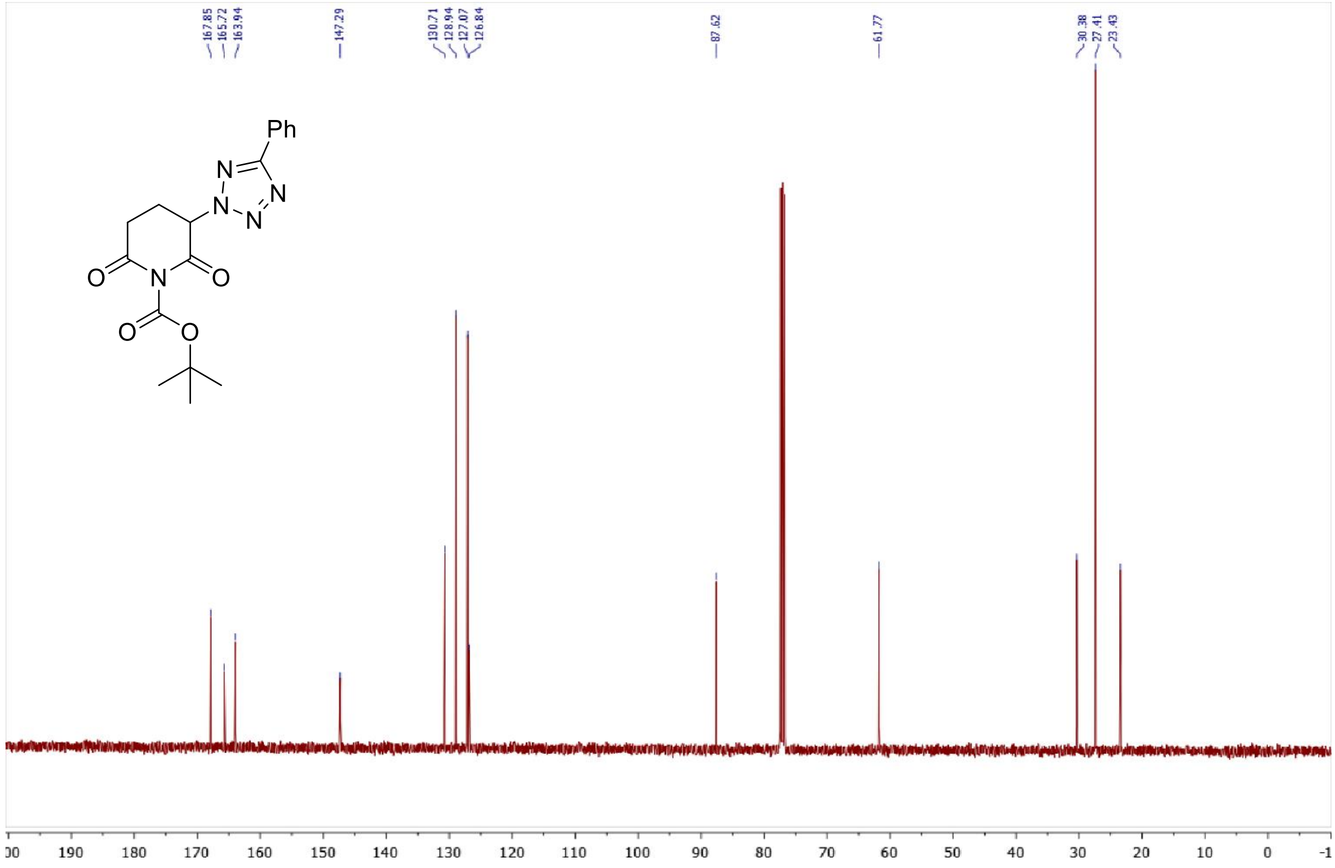

Copies of ^{1}H (400.13 MHz, $CDCl_3$) and $^{13}C\{^1H\}$ (100.61 MHz, $CDCl_3$) spectra of **6q**

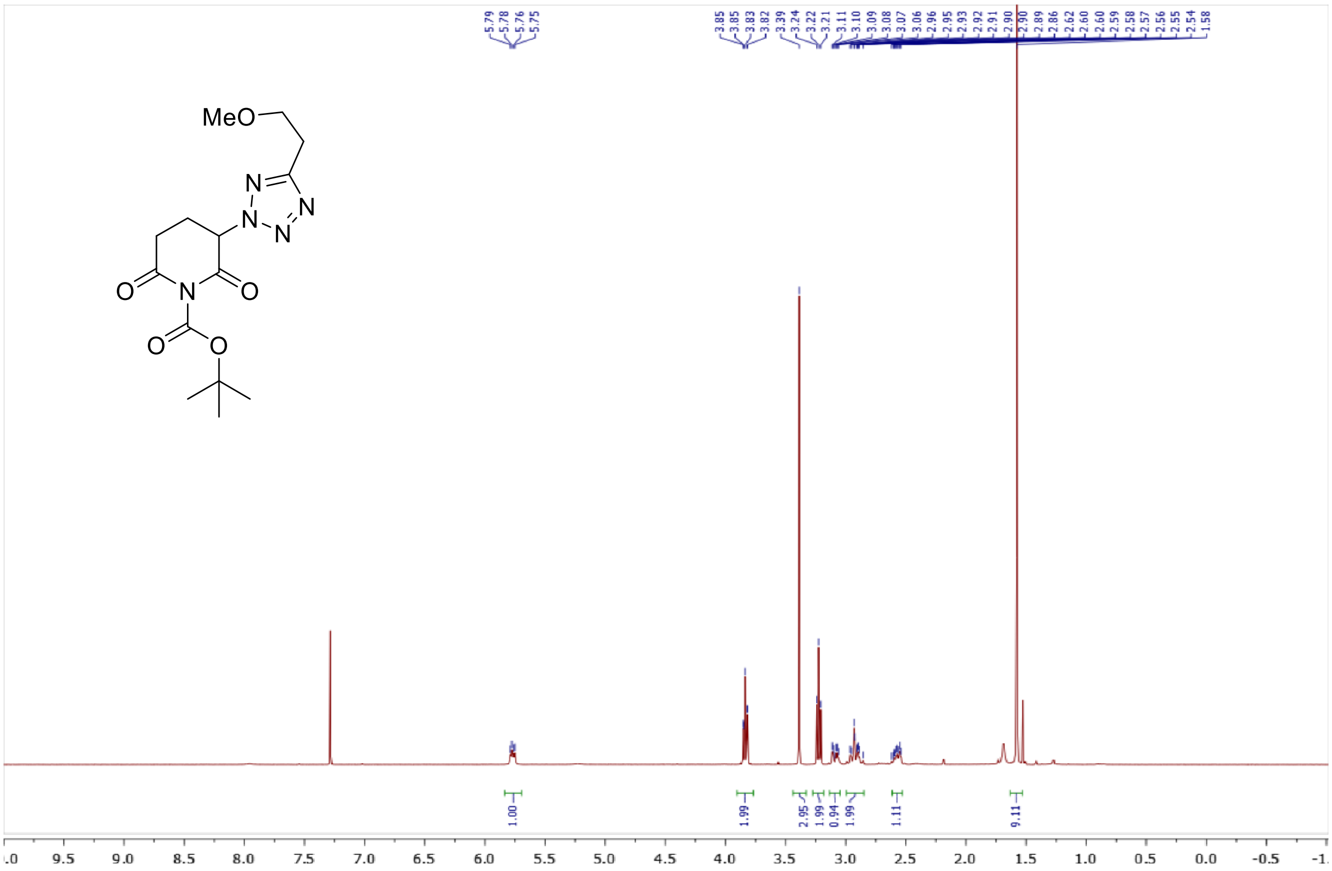

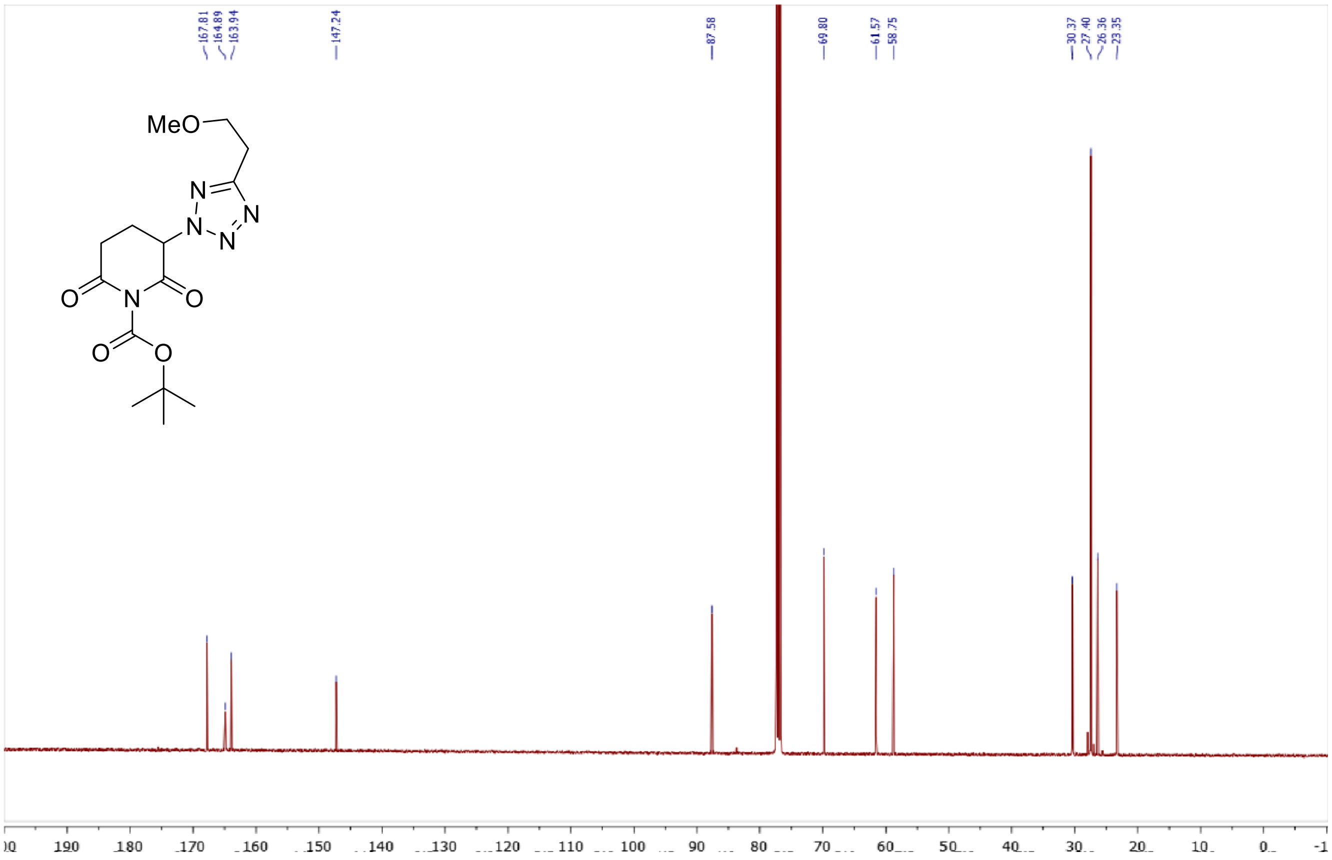

Copies of ^{1}H (400.13 MHz, $CDCl_3$) and $^{13}C\{^1H\}$ (100.61 MHz, $CDCl_3$) spectra of **6r**

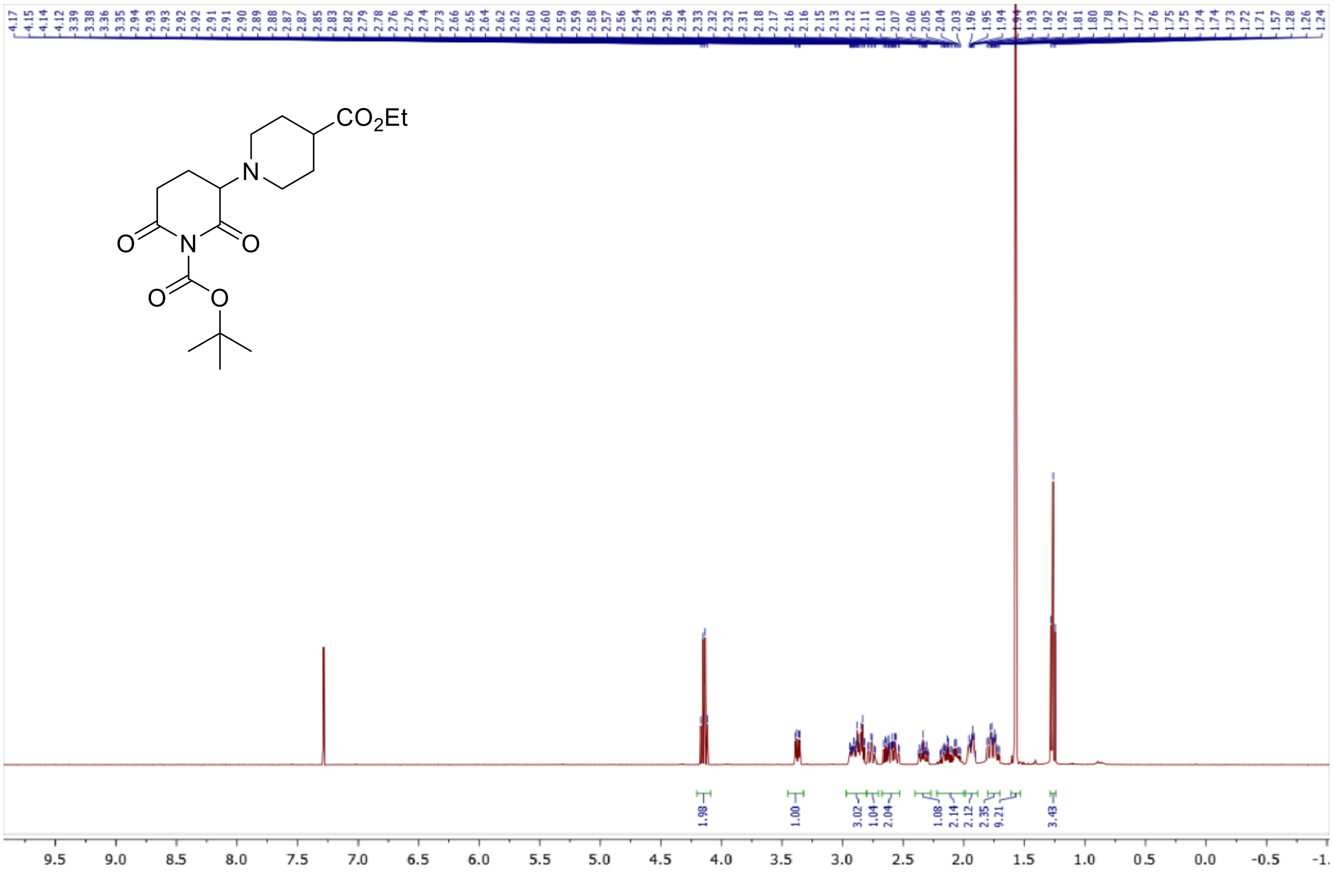

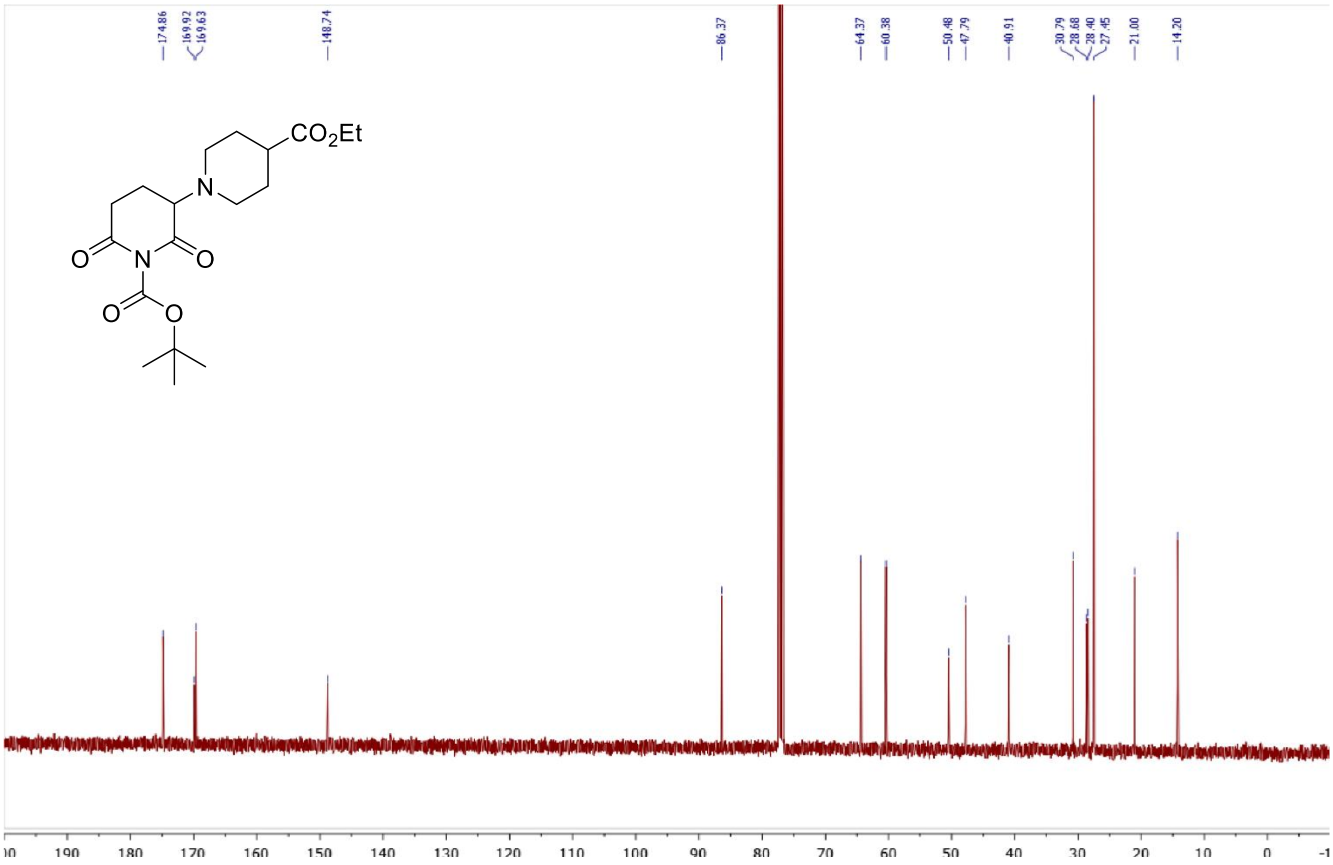

Copies of ^{1}H (400.13 MHz, $CDCl_3$) and $^{13}C\{^{1}H\}$ (100.61 MHz, $CDCl_3$) spectra of **6s**

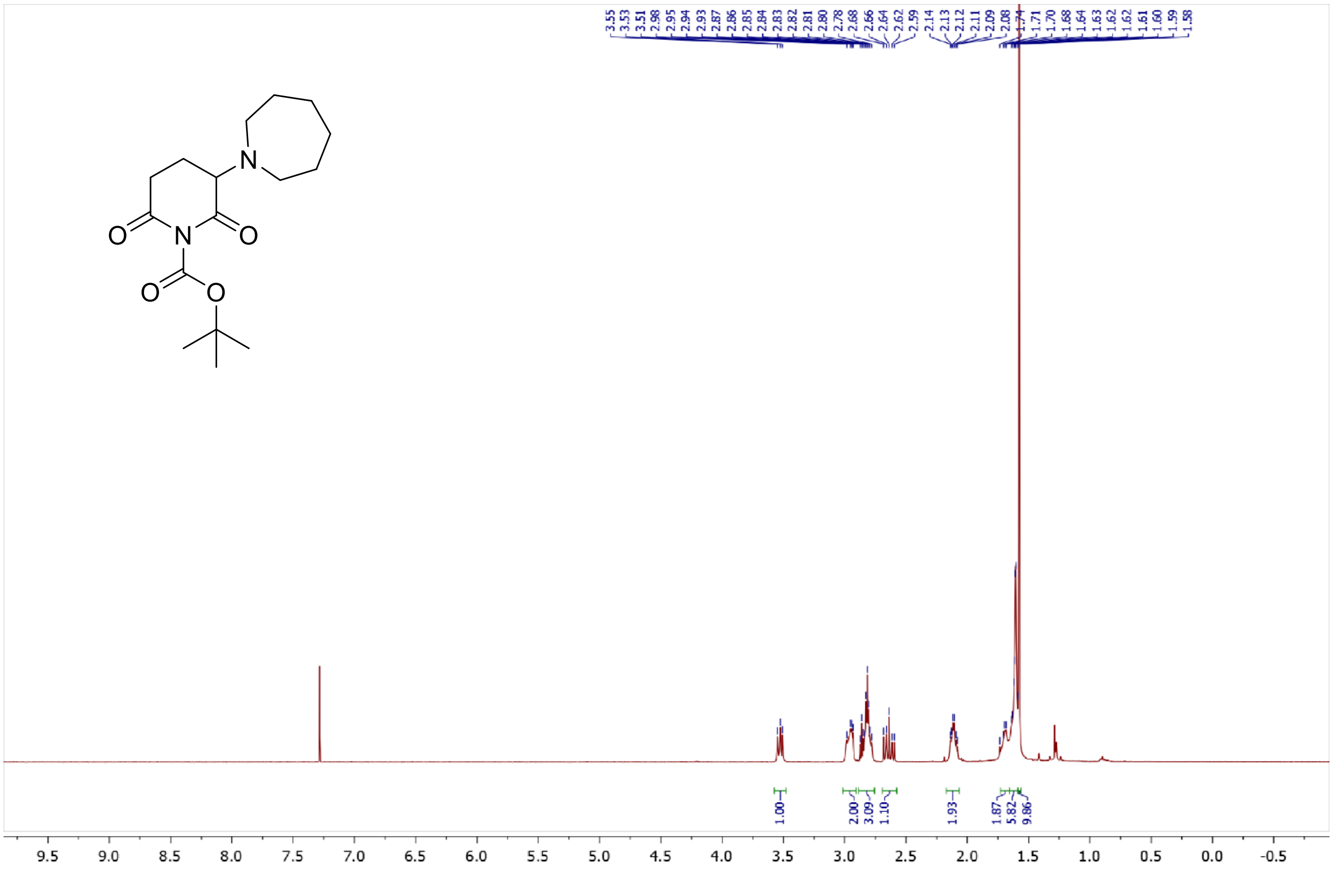

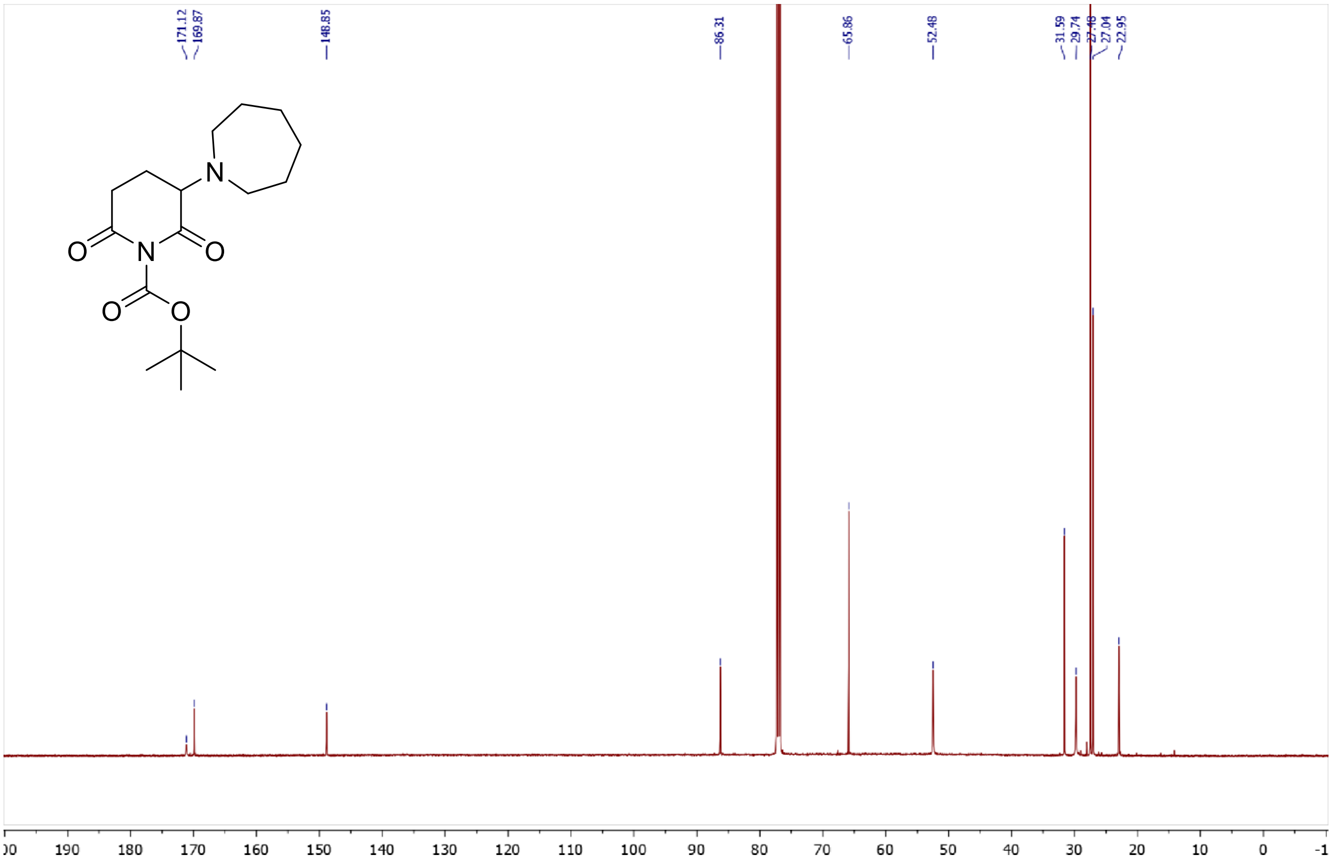

Copies of ^{1}H (400.13 MHz, $CDCl_3$) and $^{13}C\{^{1}H\}$ (100.61 MHz, $CDCl_3$) spectra of **6t**

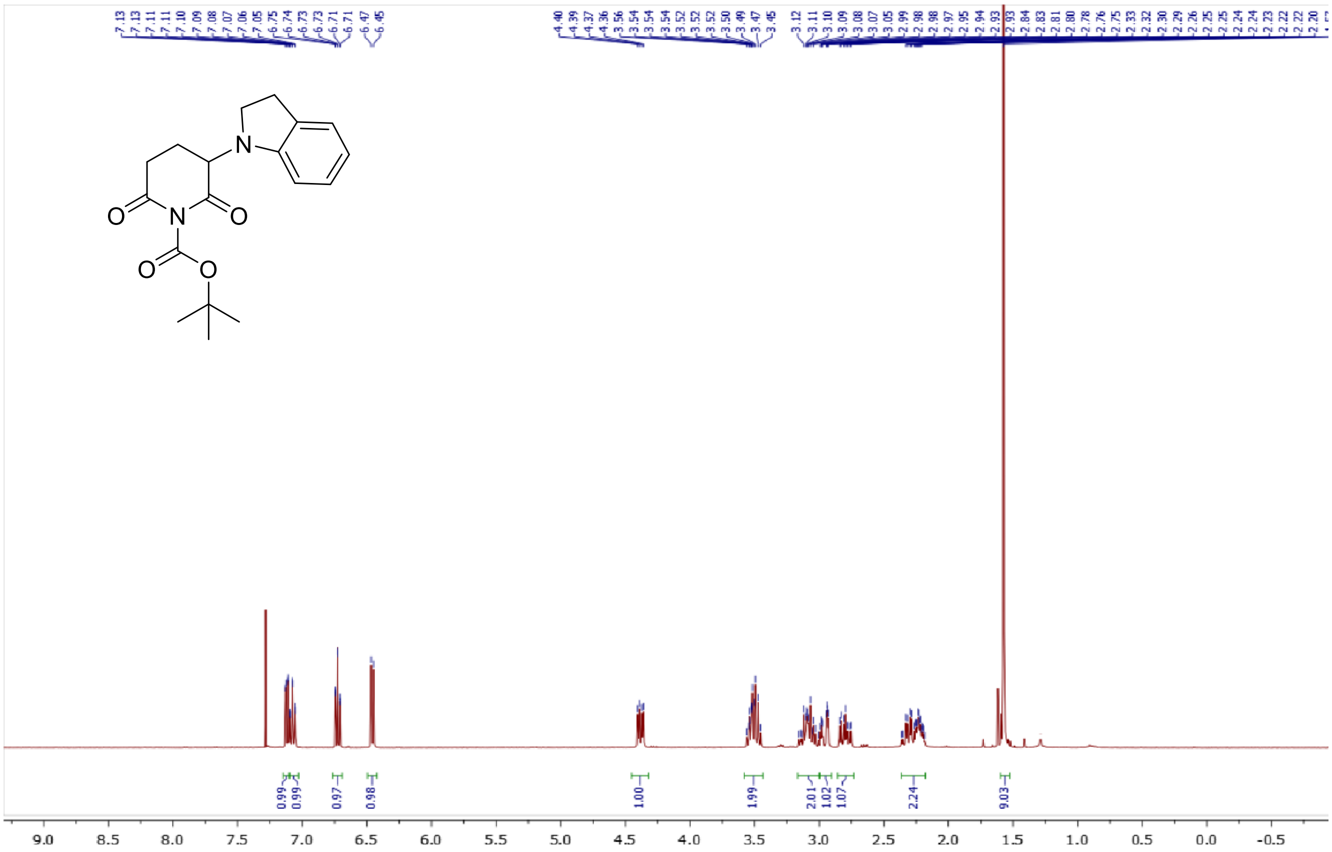

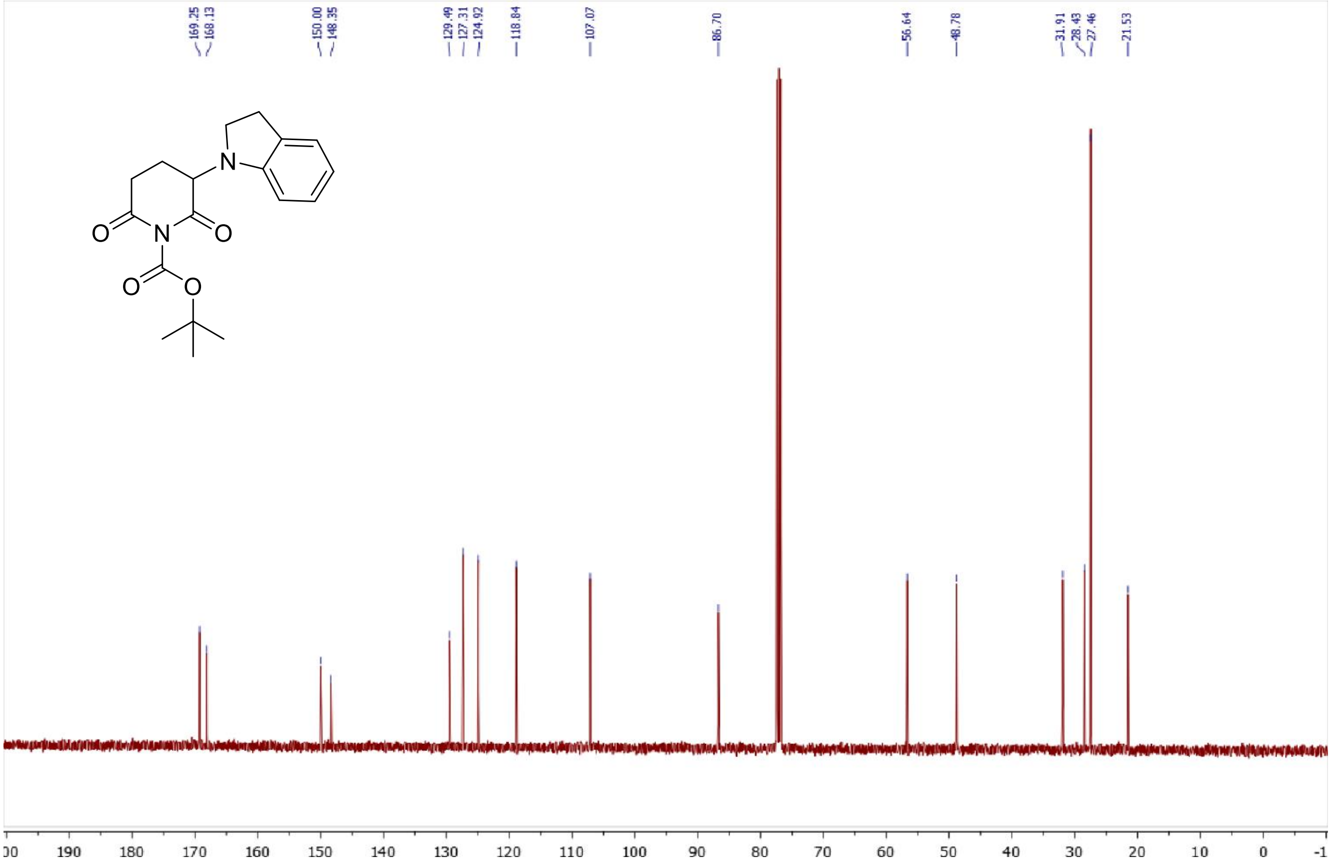

Copies of ^{1}H (400.13 MHz, $CDCl_3$) and $^{13}C\{^{1}H\}$ (100.61 MHz, $CDCl_3$) spectra of **6u**

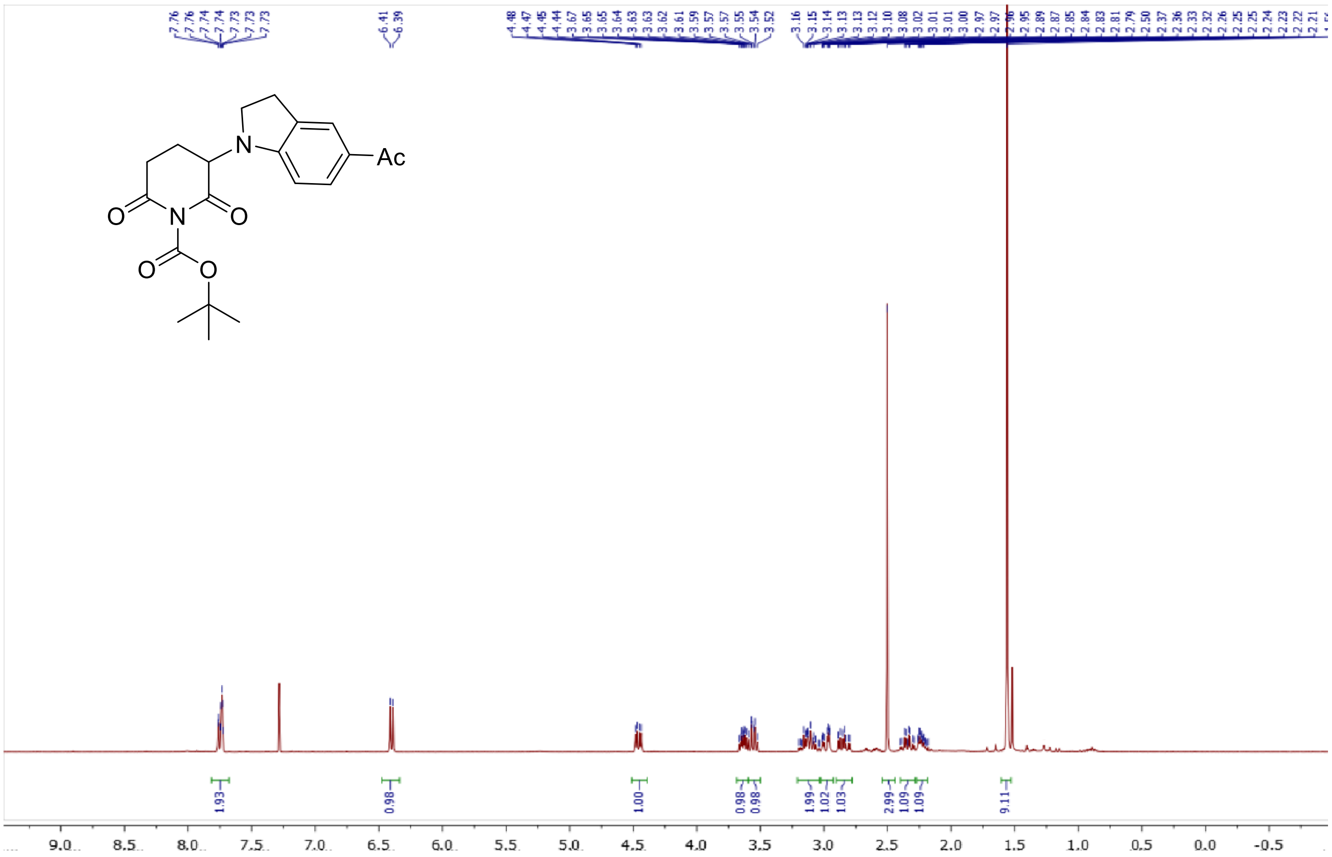

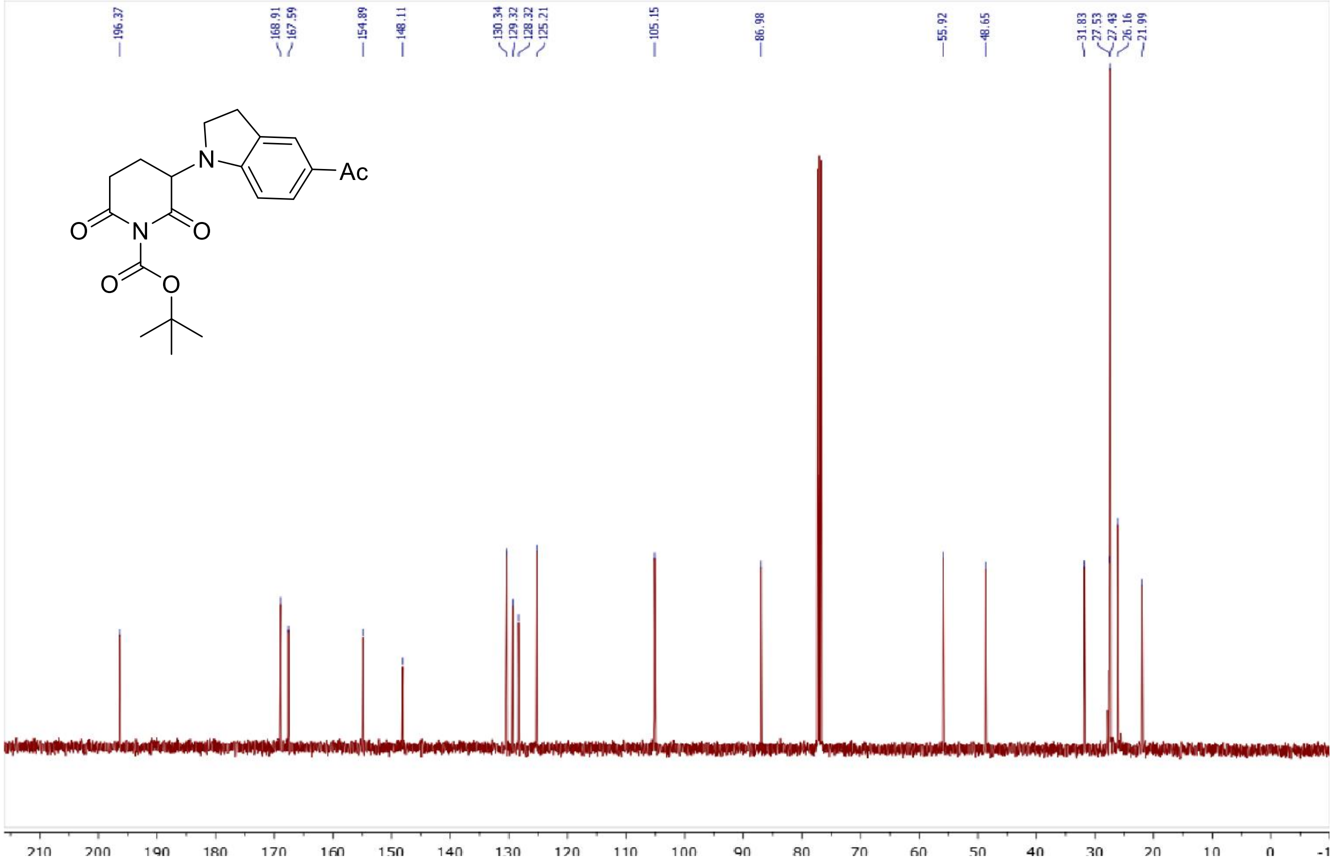

Copies of ^{1}H (400.13 MHz, $CDCl_3$) and $^{13}C\{^{1}H\}$ (100.61 MHz, $CDCl_3$) spectra of **6v**

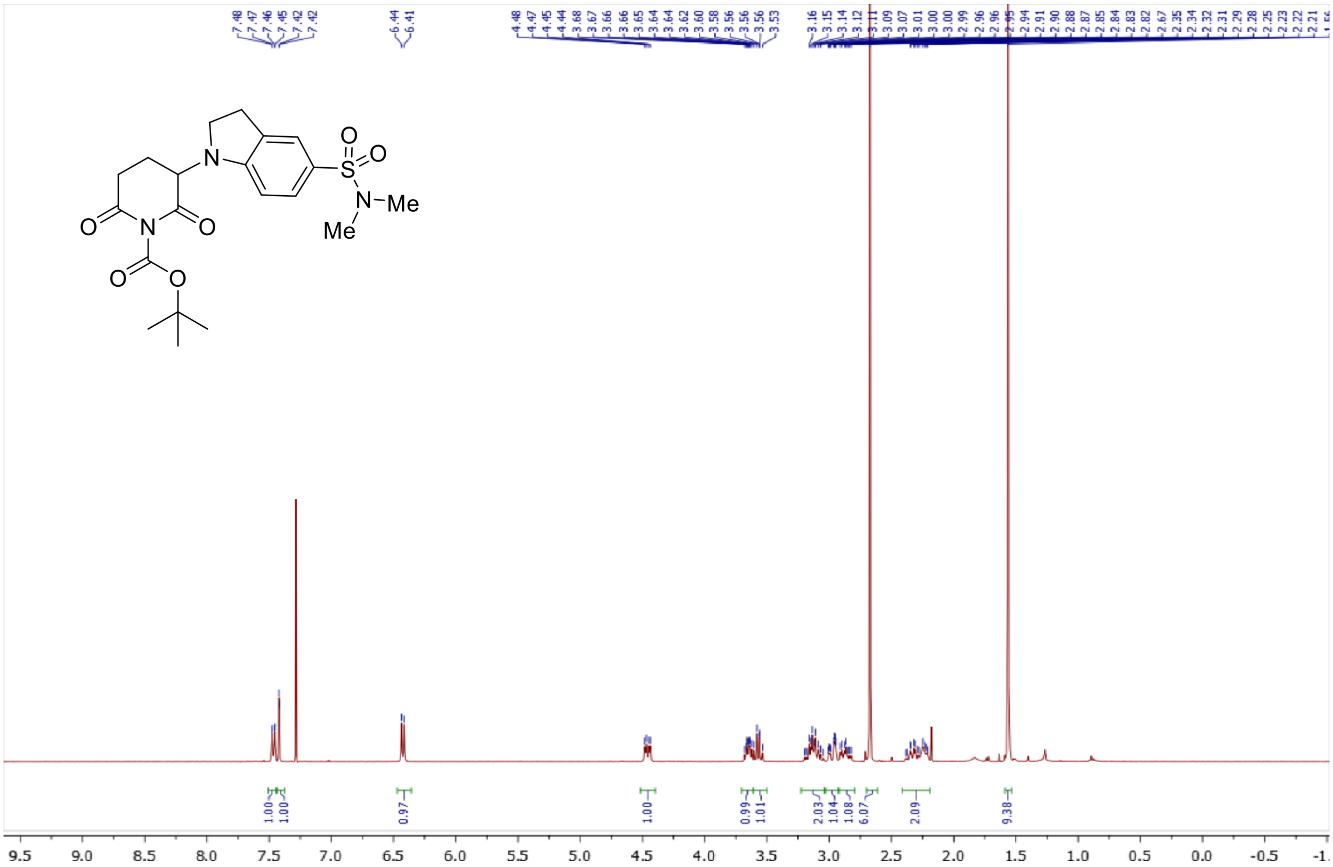

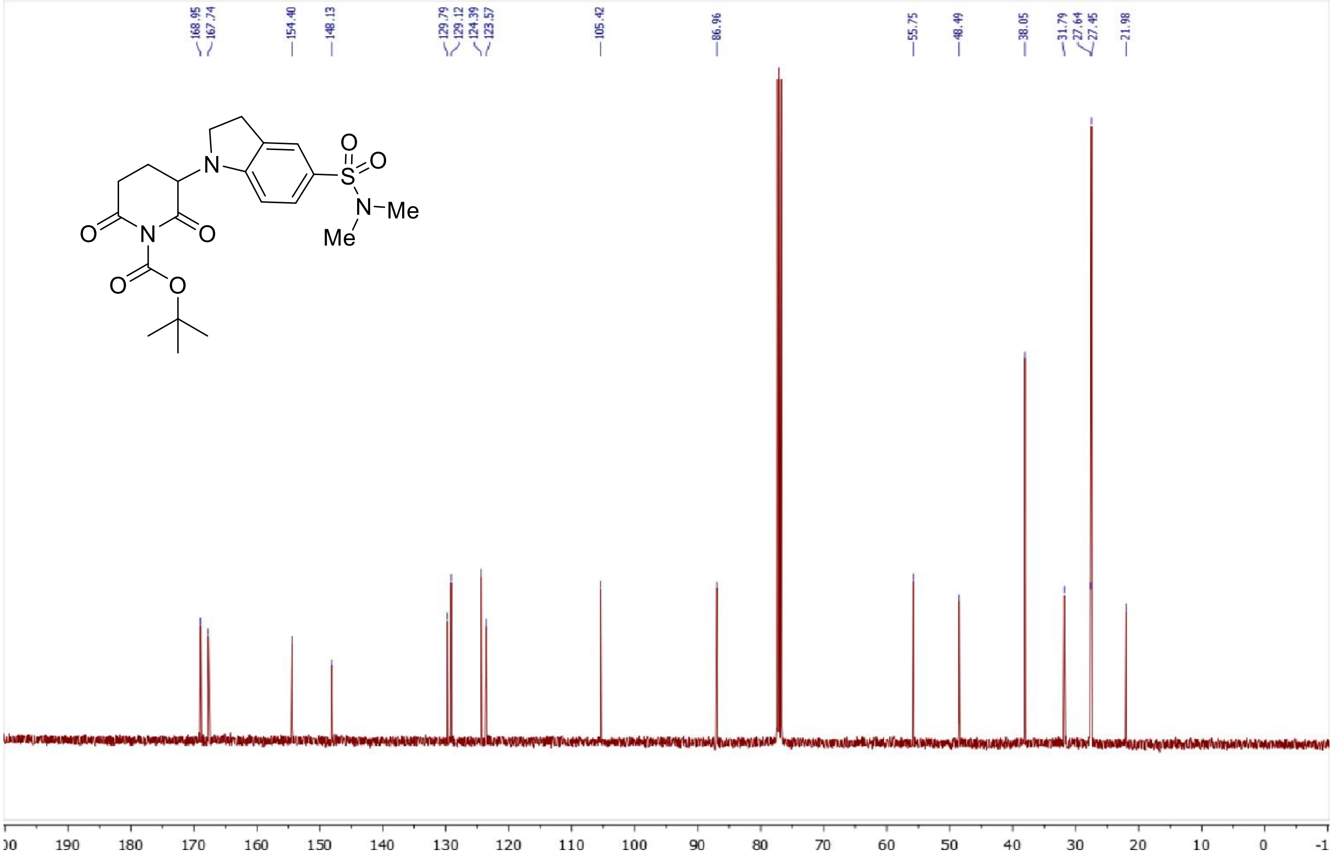

Copies of ^{1}H (400.13 MHz, $CDCl_3$) and ^{13}C{^{1}H} (100.61 MHz, $CDCl_3$) spectra of **6w**

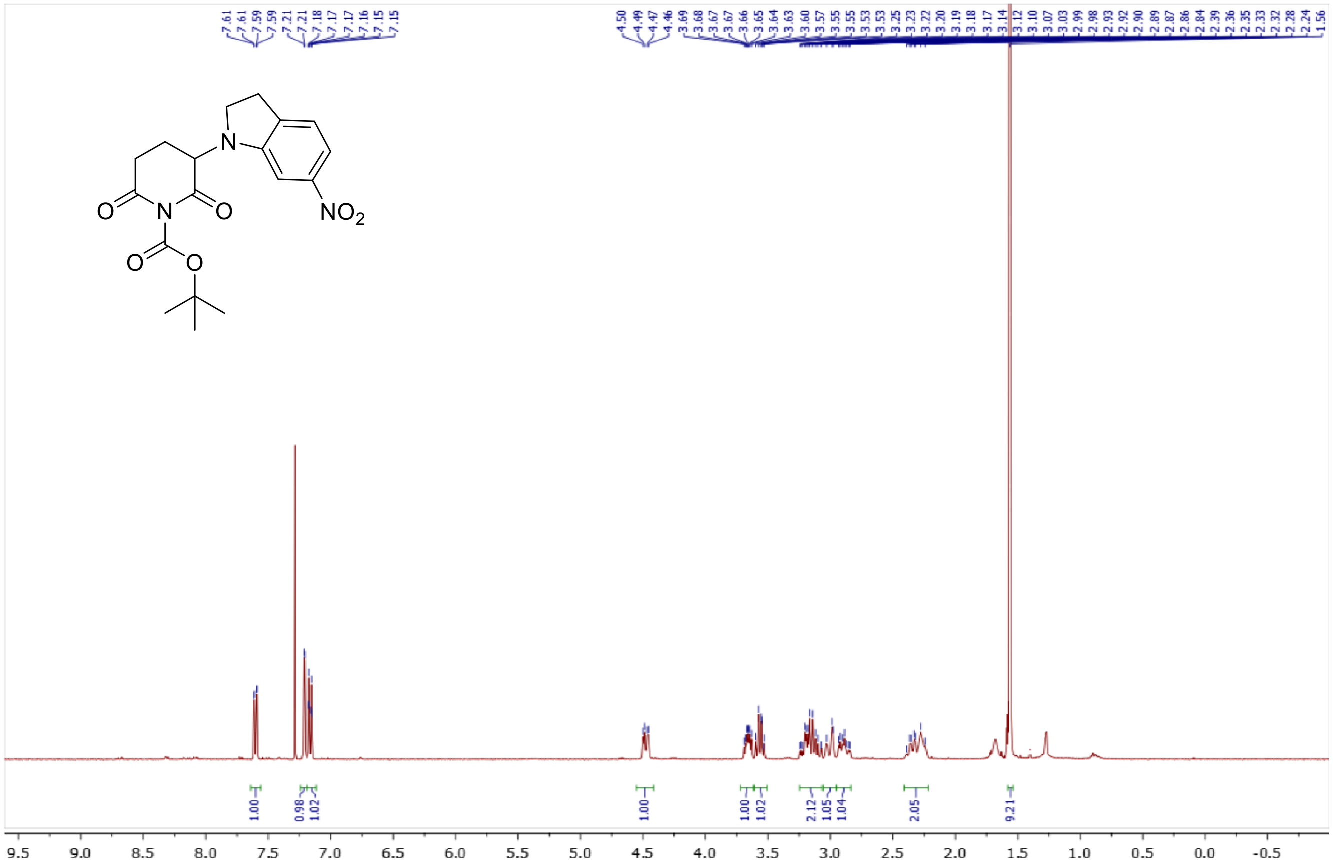

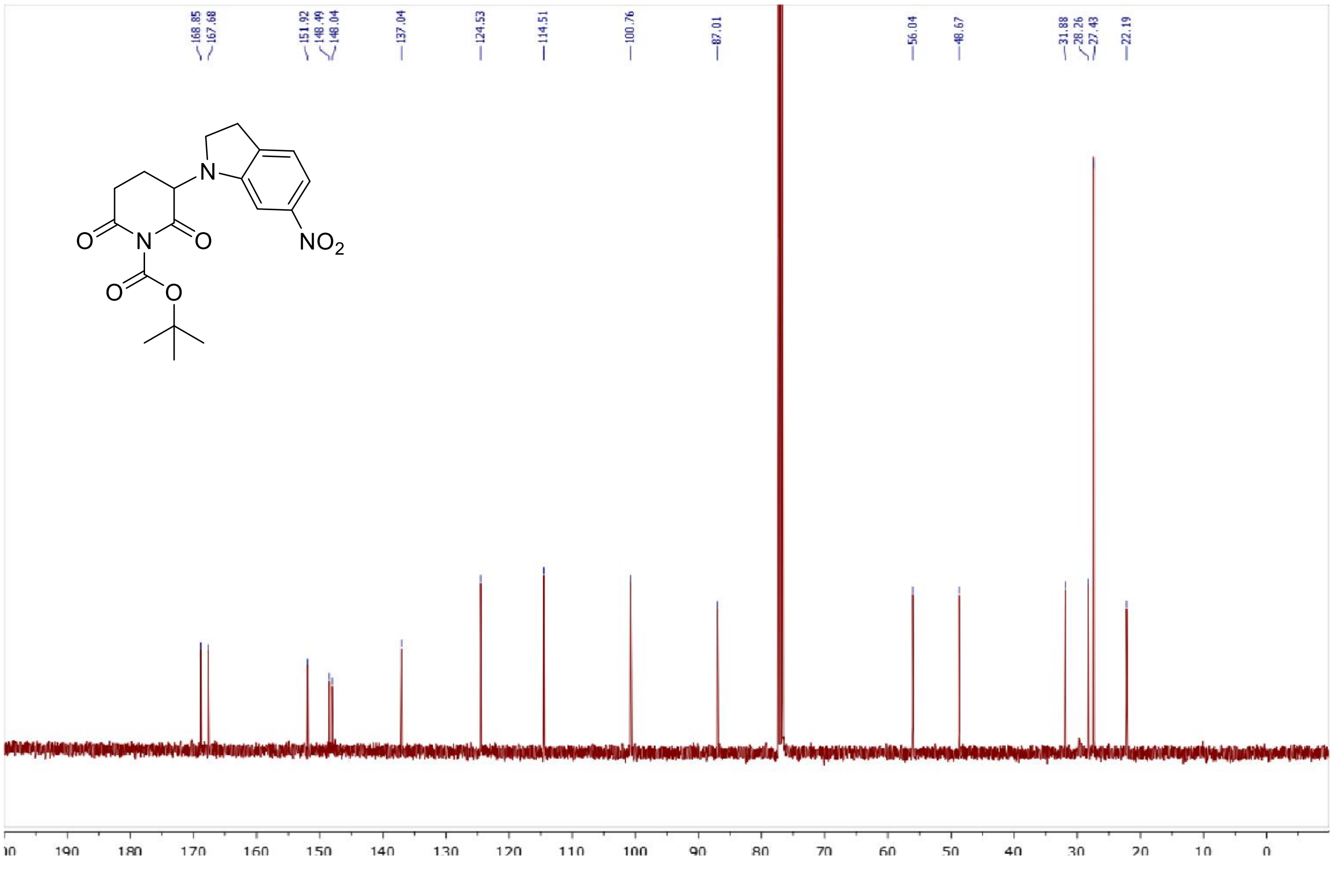

Copies of ^{1}H (400.13 MHz, CDCl$_3$) and ^{13}C{^{1}H} (100.61 MHz, CDCl$_3$) spectra of **6x**

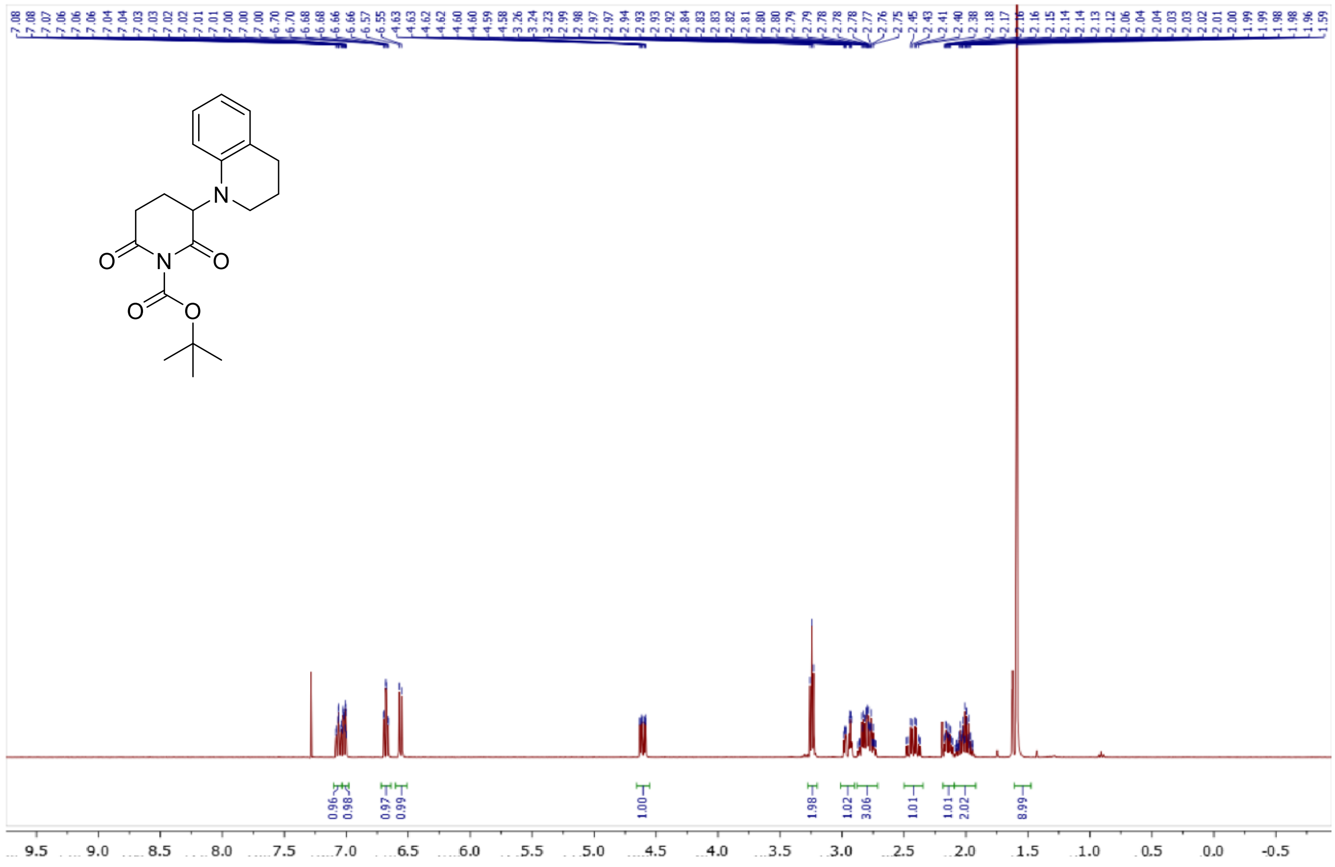

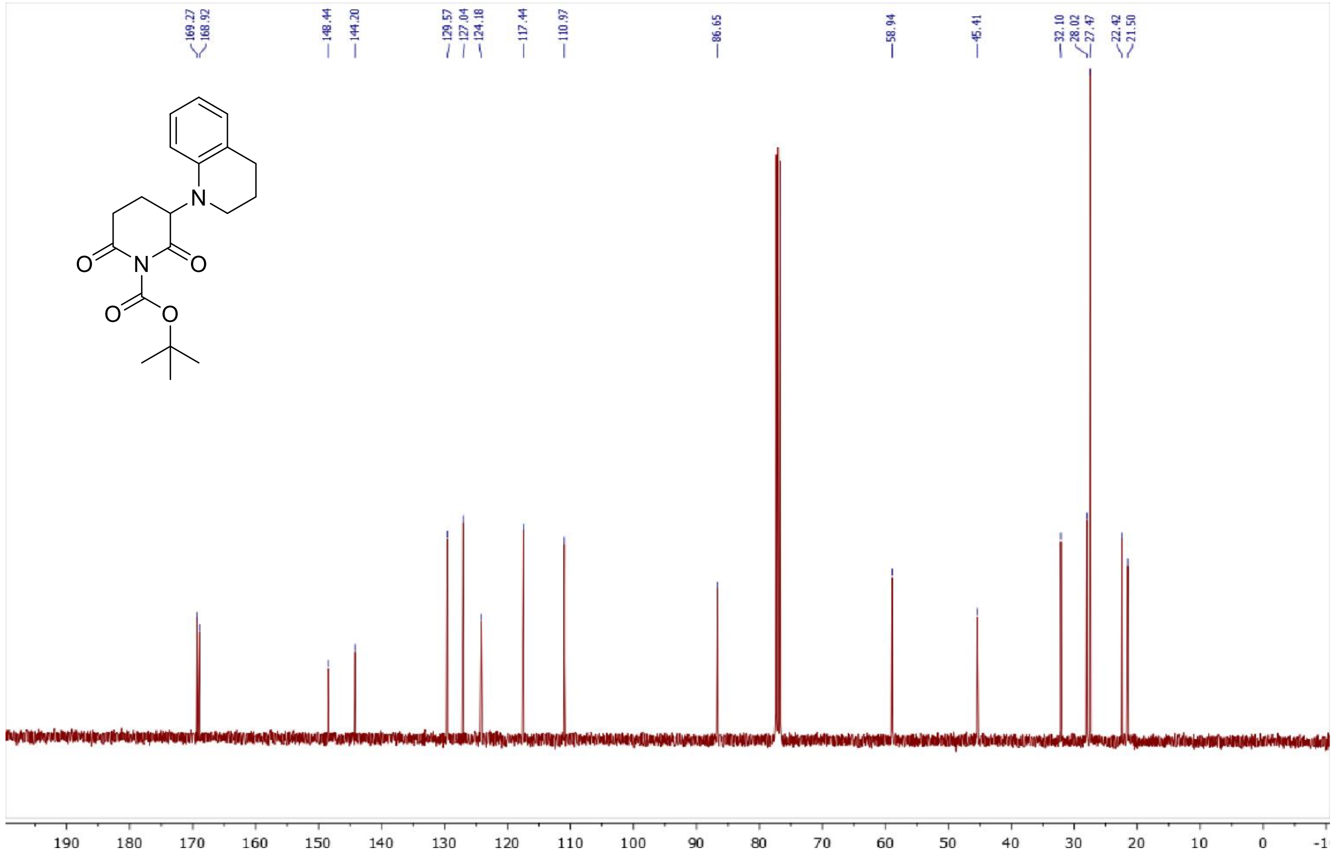

Copies of ^{1}H (400.13 MHz, $CDCl_3$) and $^{13}C\{^{1}H\}$ (100.61 MHz, $CDCl_3$) spectra of **6y**

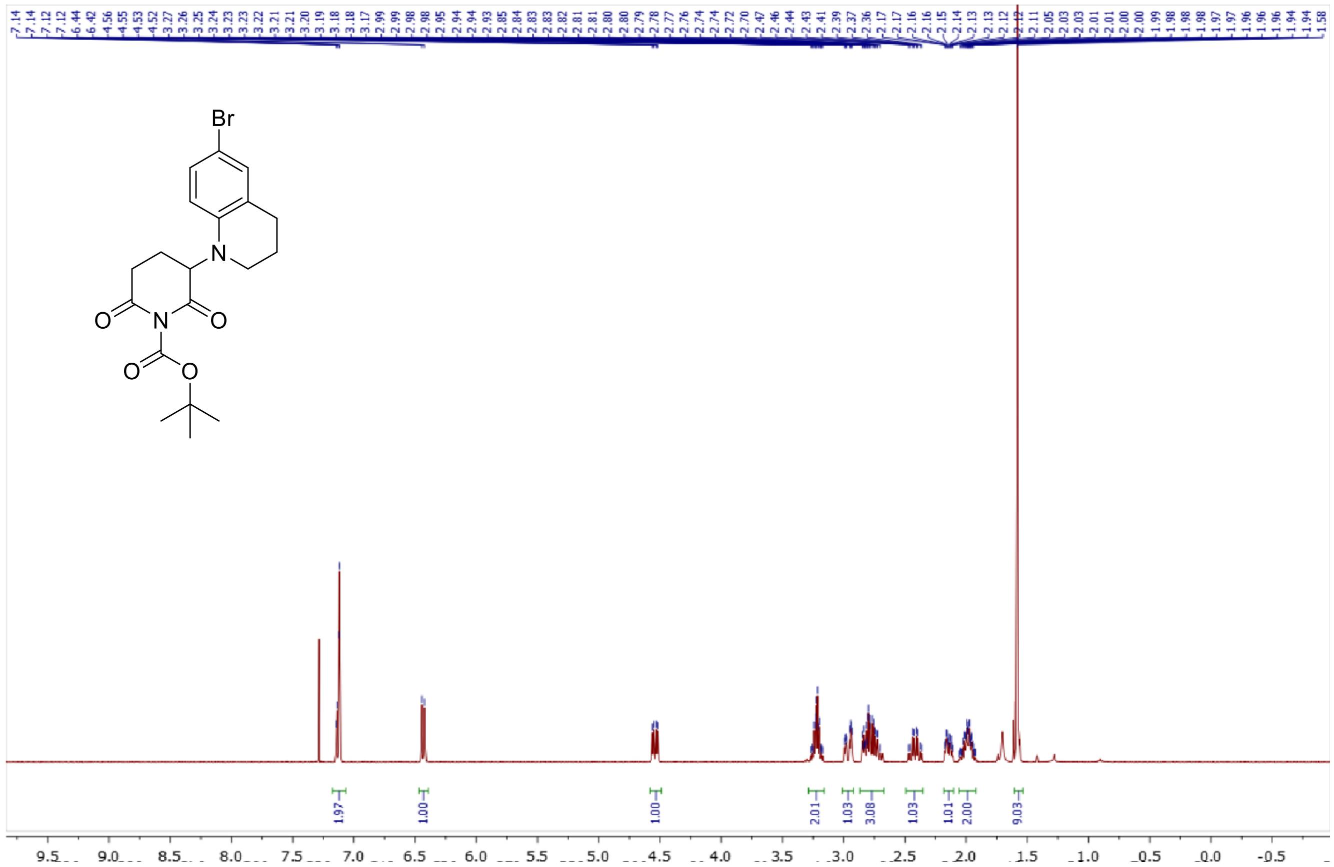

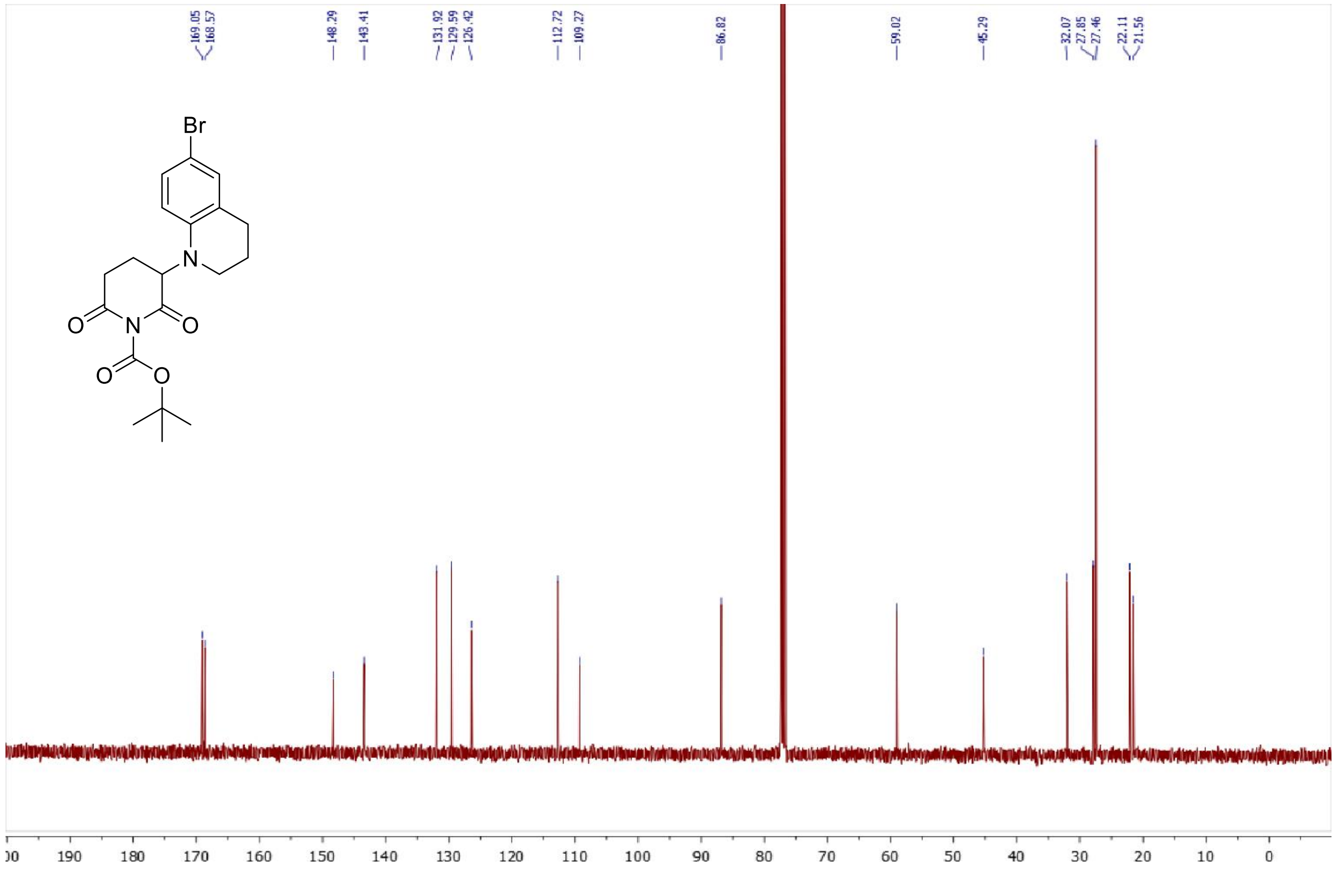

Copies of ^{1}H (400.13 MHz, $CDCl_3$) and ^{13}C{^{1}H} (100.61 MHz, $CDCl_3$) spectra of **9z**

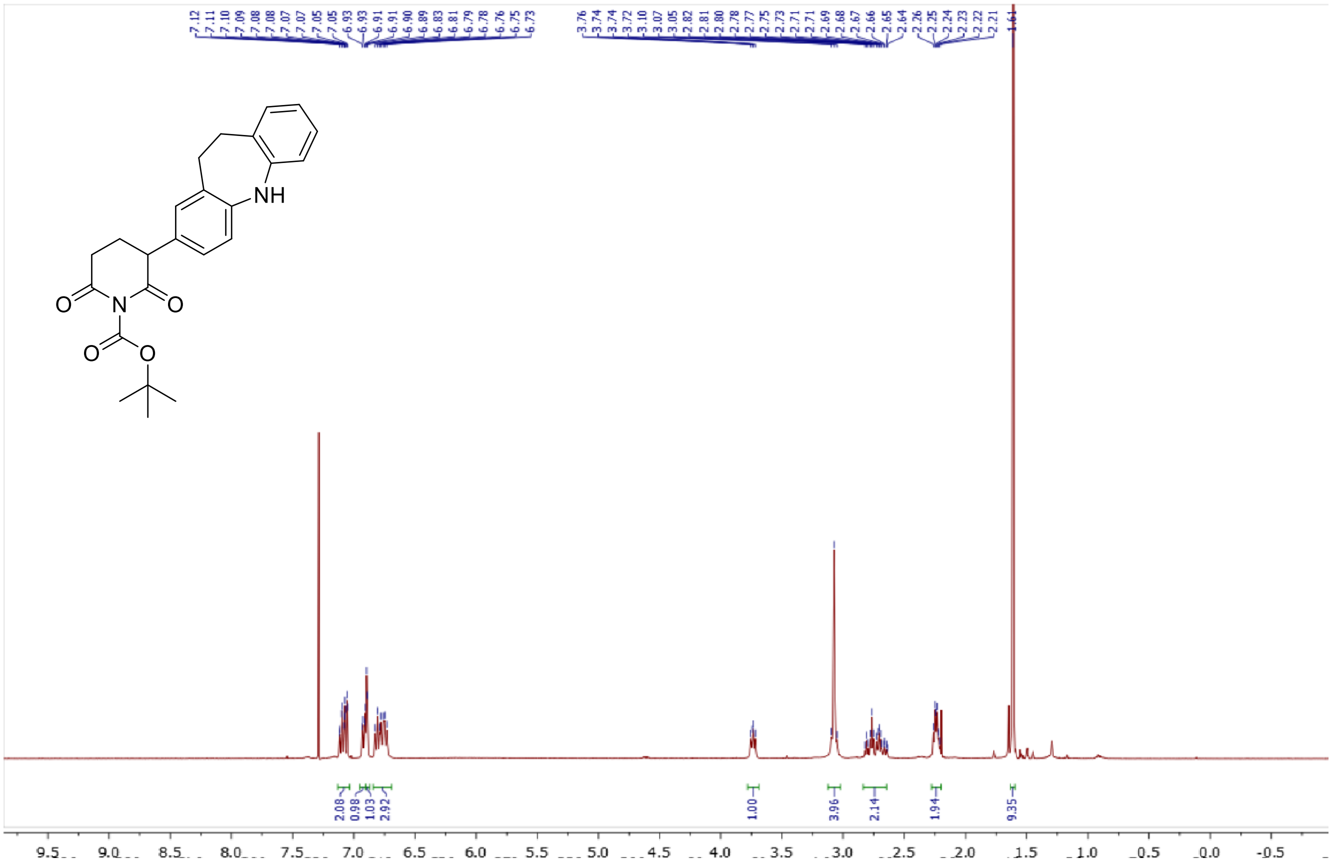

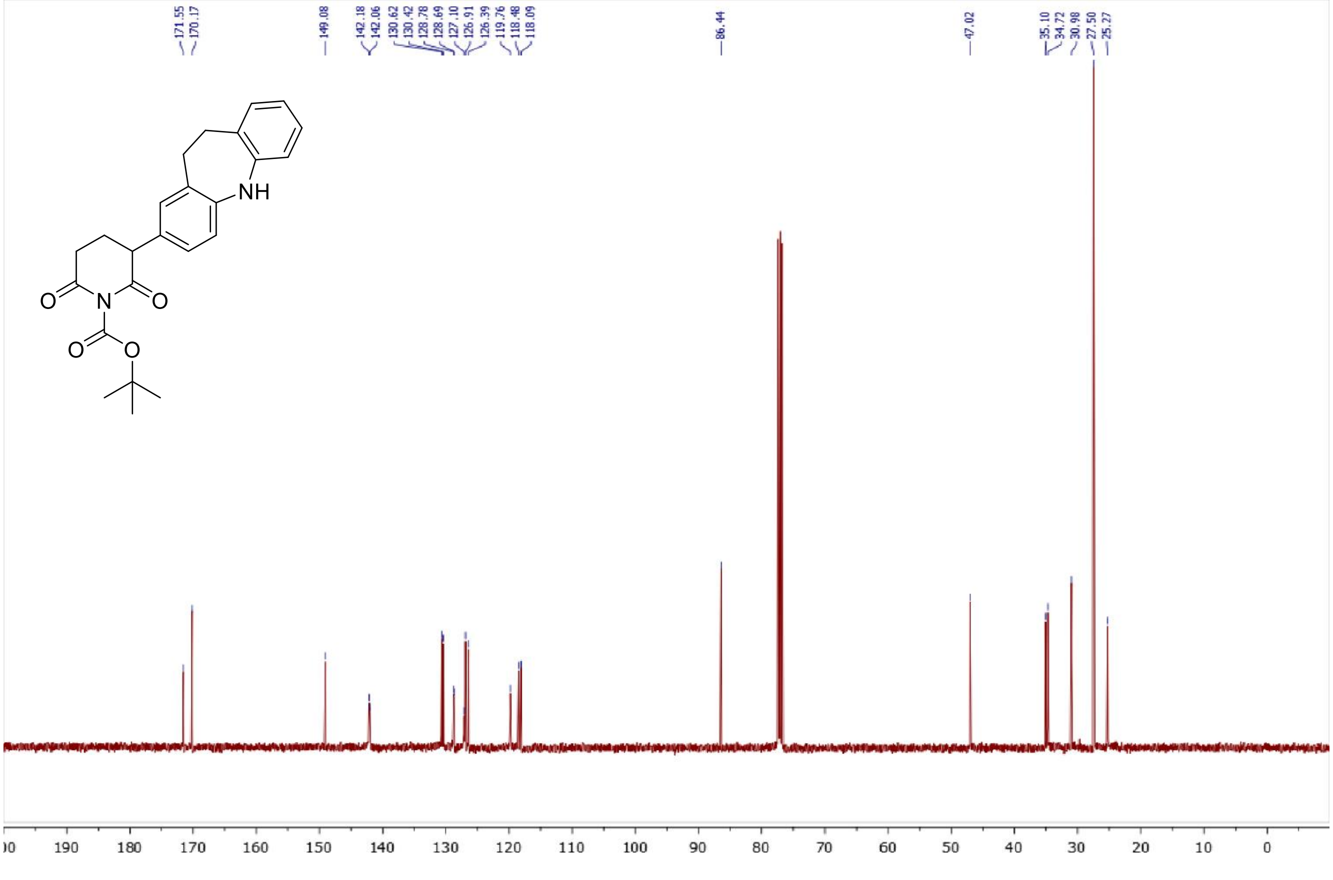

Copies of ^{1}H (400.13 MHz, DMSO-d_6) and $^{13}C\{^{1}H\}$ (101.61 MHz, DMSO-d_6) spectra of **1a**

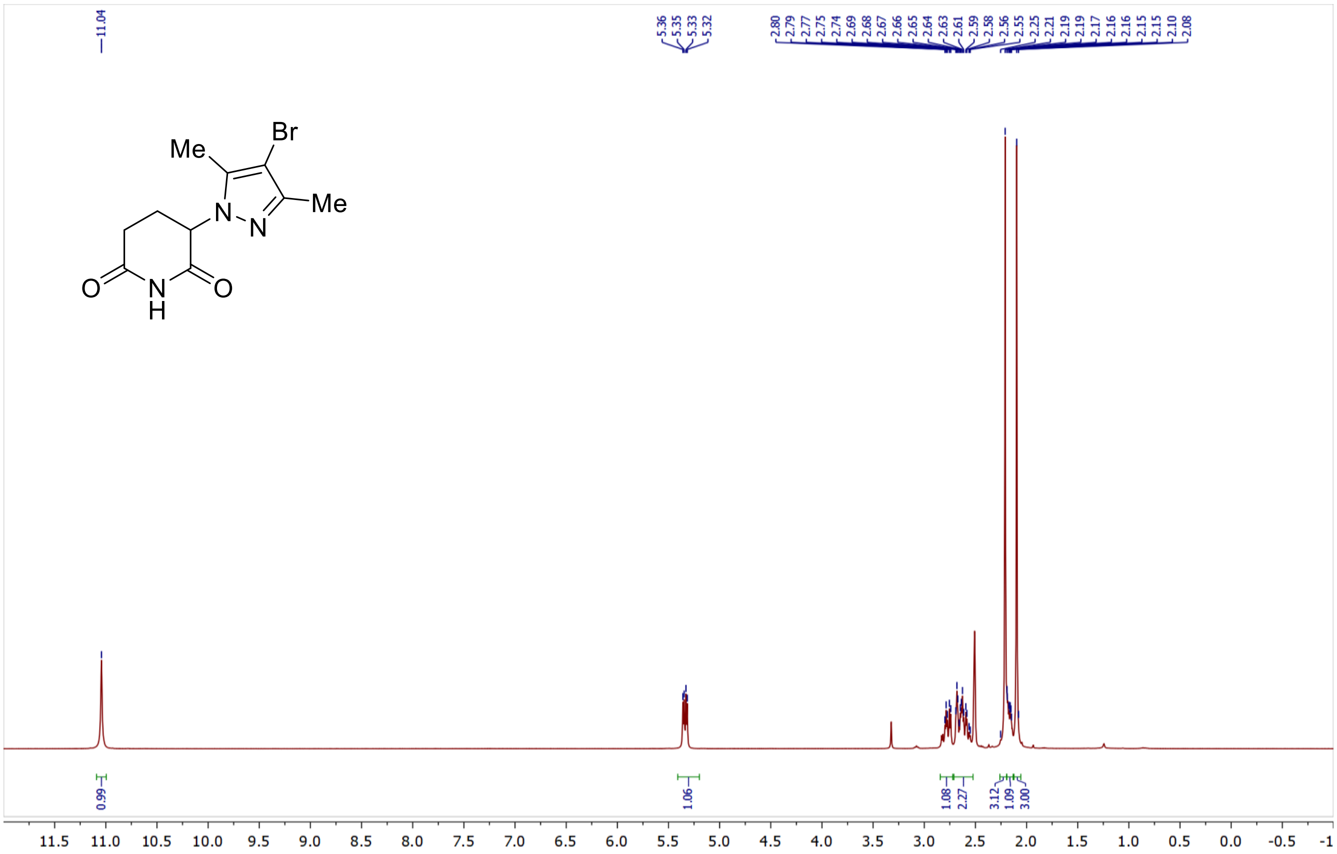

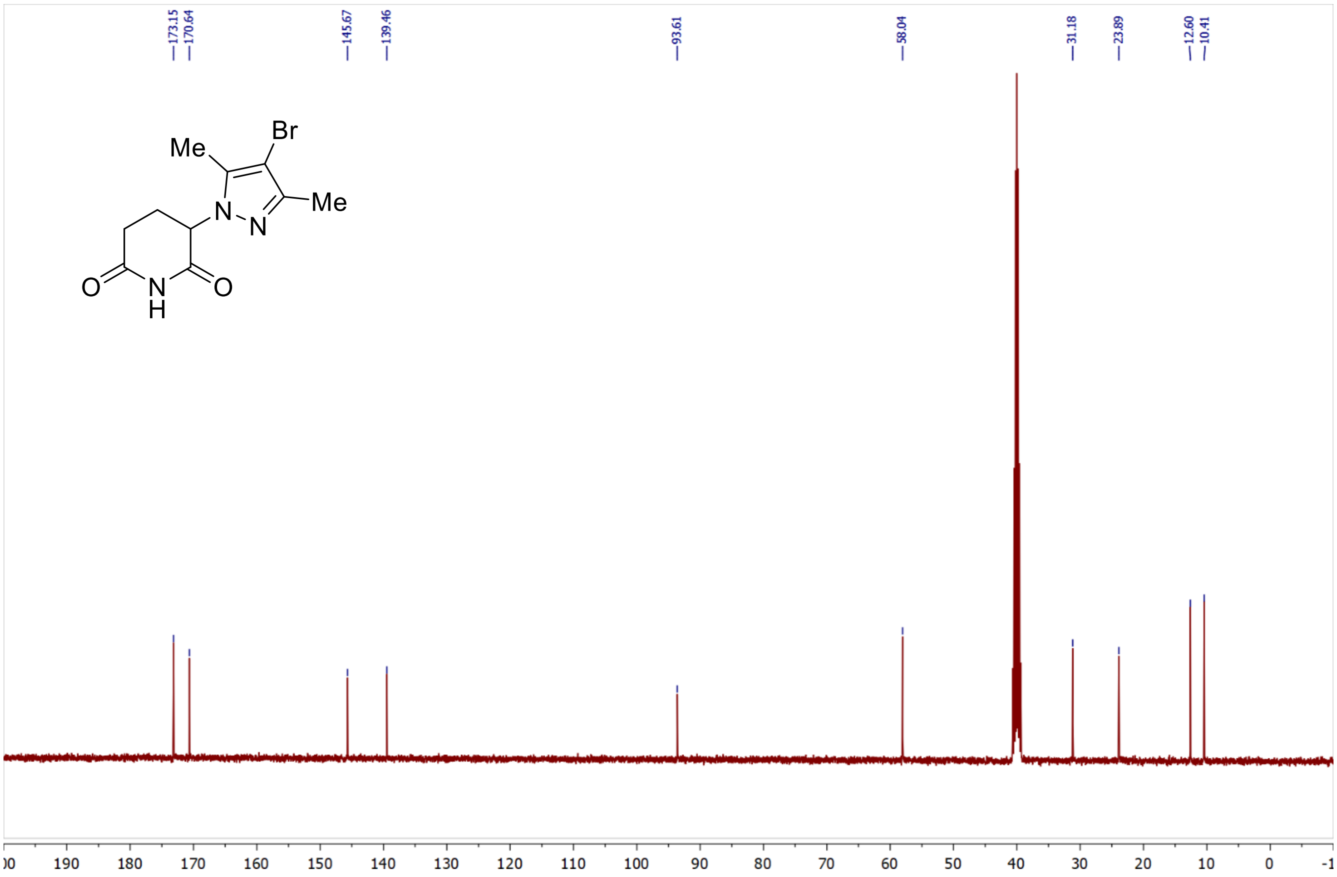

Copies of ^{1}H (400.13 MHz, DMSO-d_6) spectra of **1b**

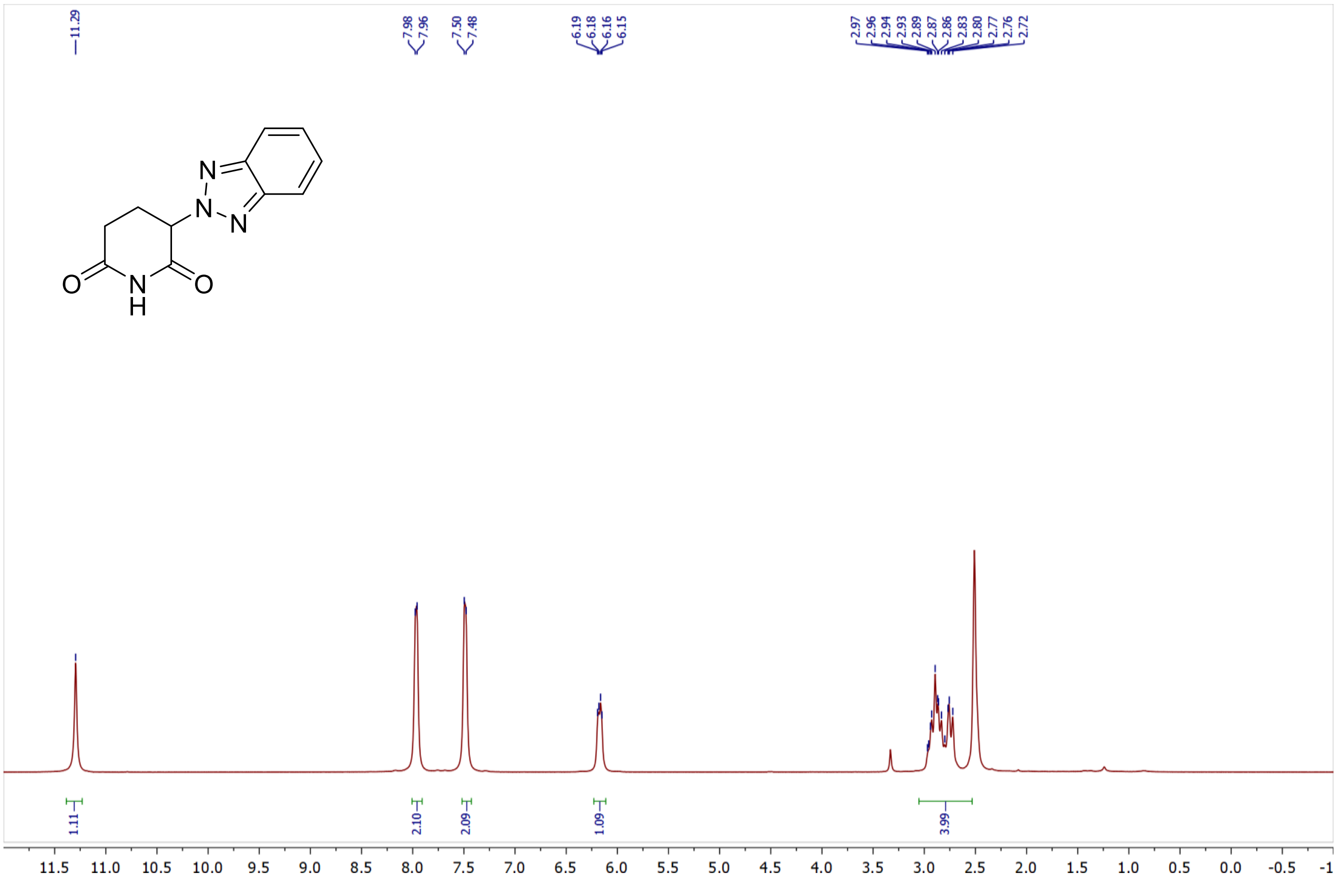

Copies of ^{1}H (400.13 MHz, DMSO-d_6) and $^{13}C\{^{1}H\}$ (101.61 MHz, DMSO-d_6) spectra of **1c**

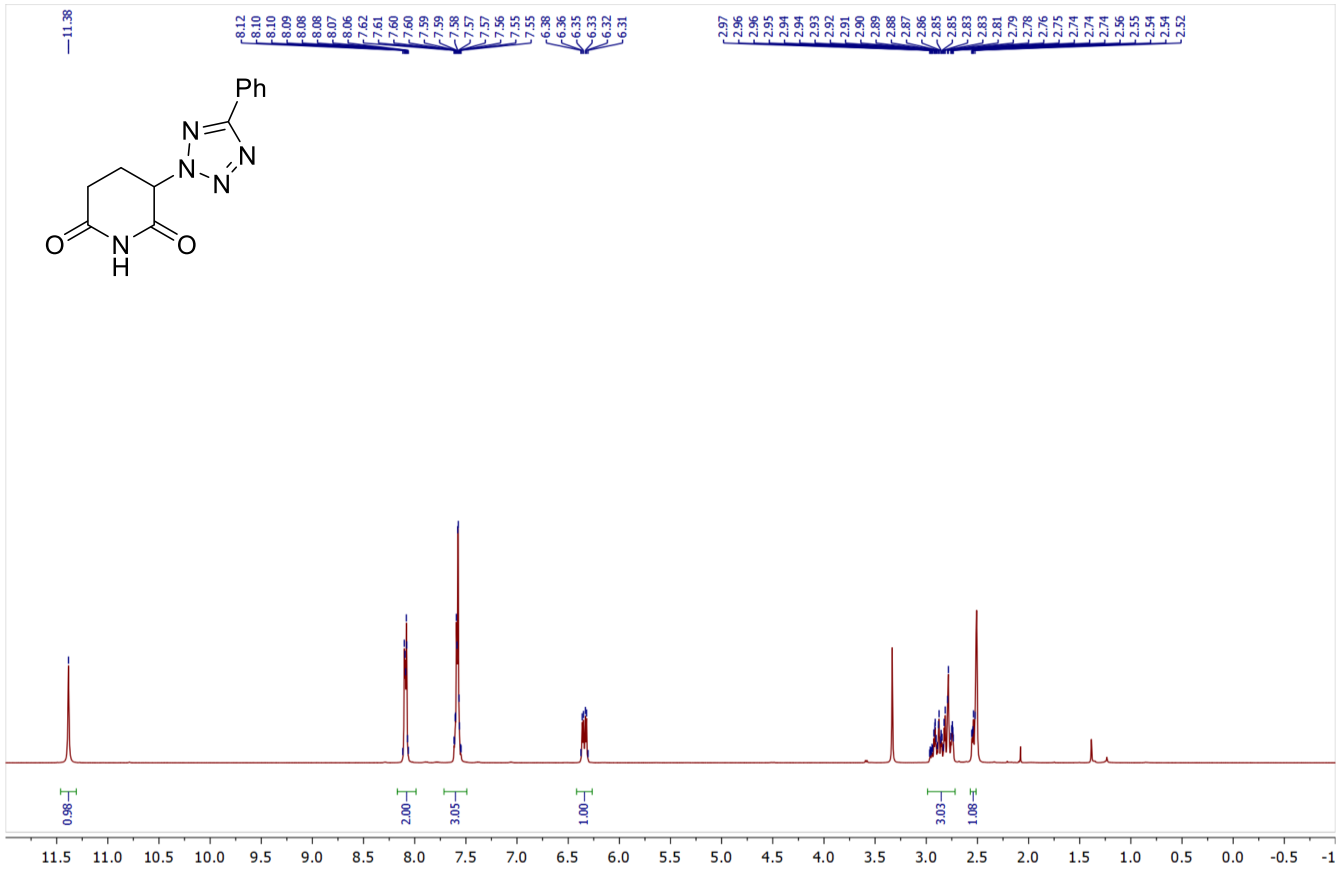

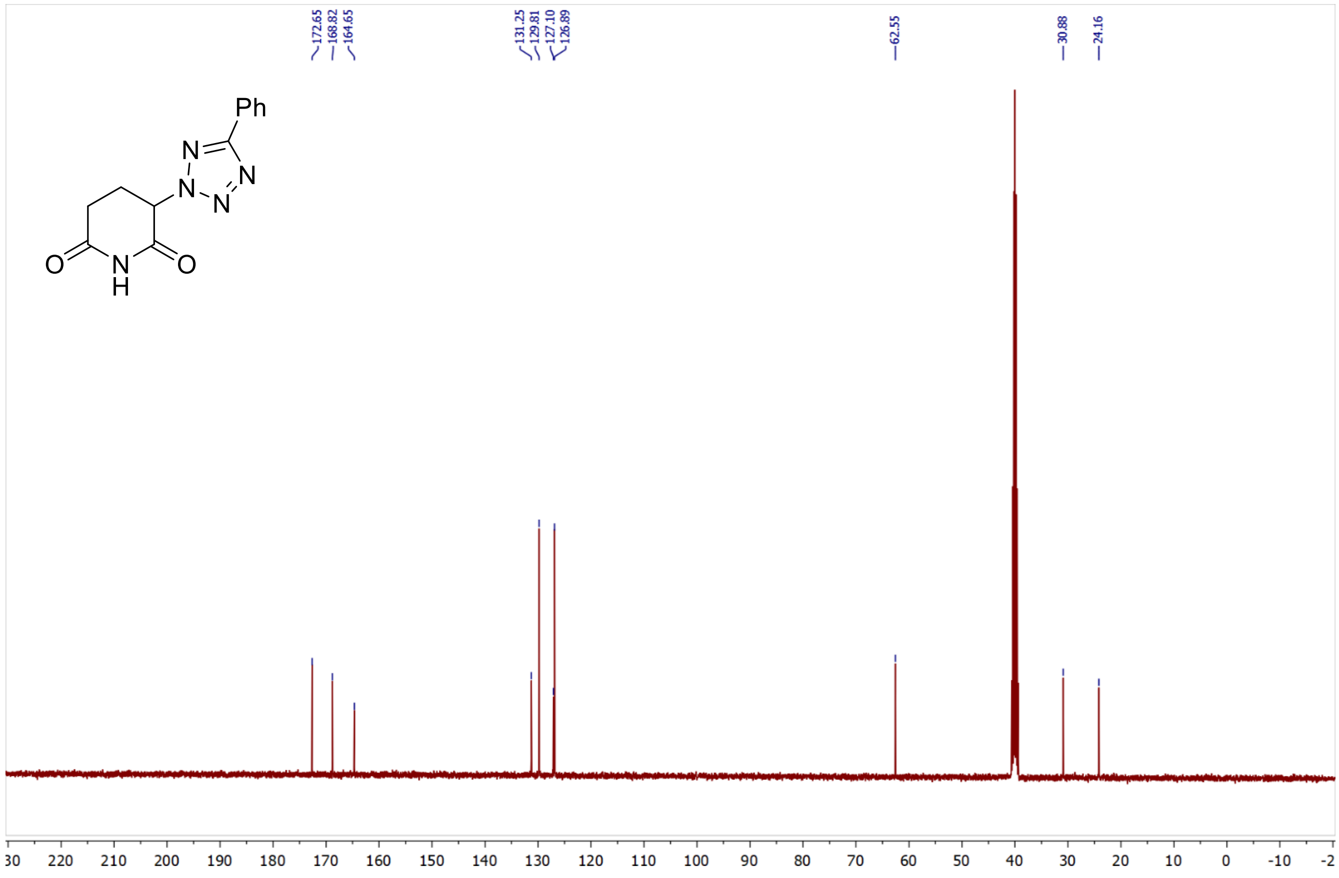

Copies of ^{1}H (400.13 MHz, Acetone-d_6) and ^{13}C{^{1}H} (101.61 MHz, DMSO-d_6) spectra of **1d**

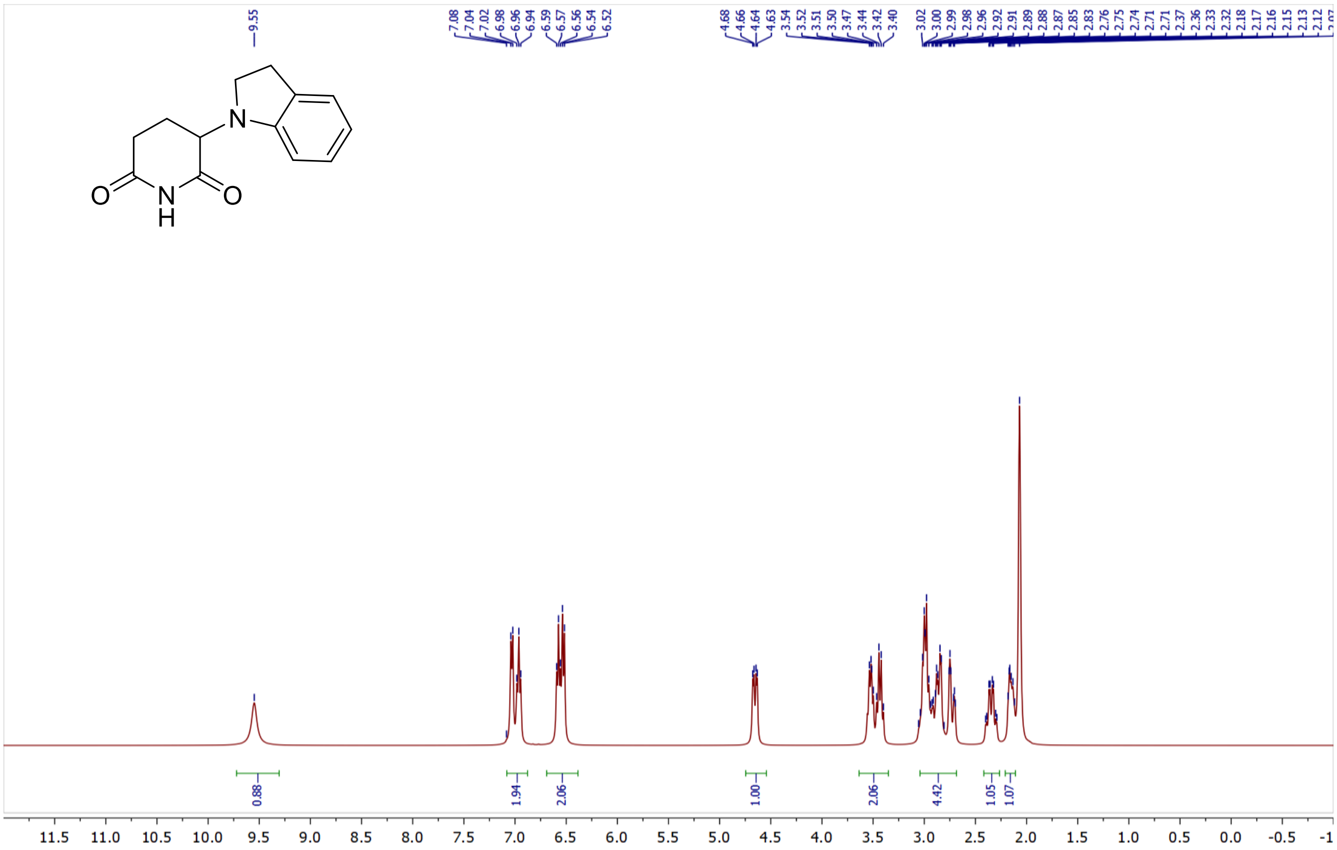

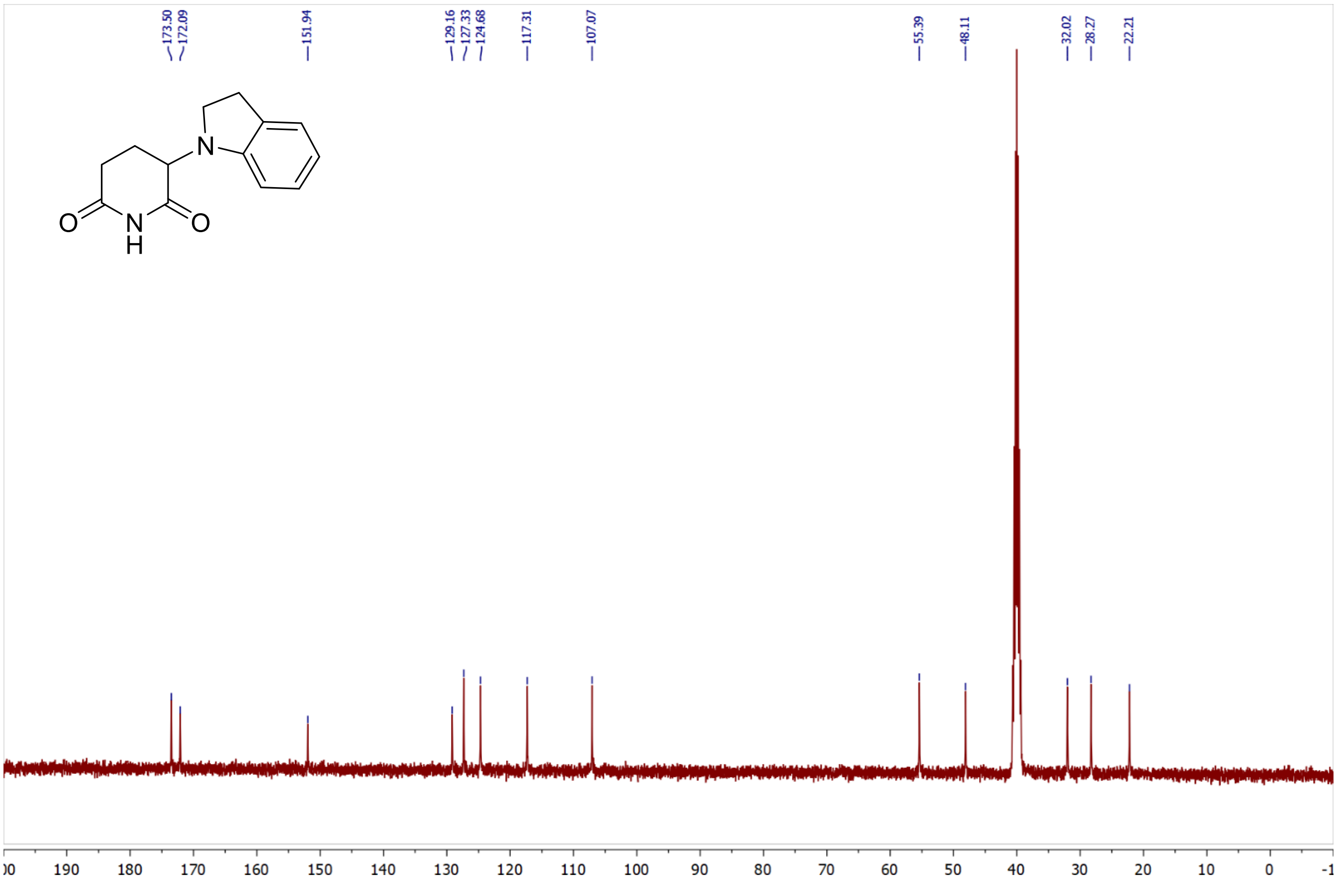

Copies of ^{1}H (400.13 MHz, DMSO-d_6) and ^{13}C{^{1}H} (101.61 MHz, DMSO-d_6) spectra of **1e**

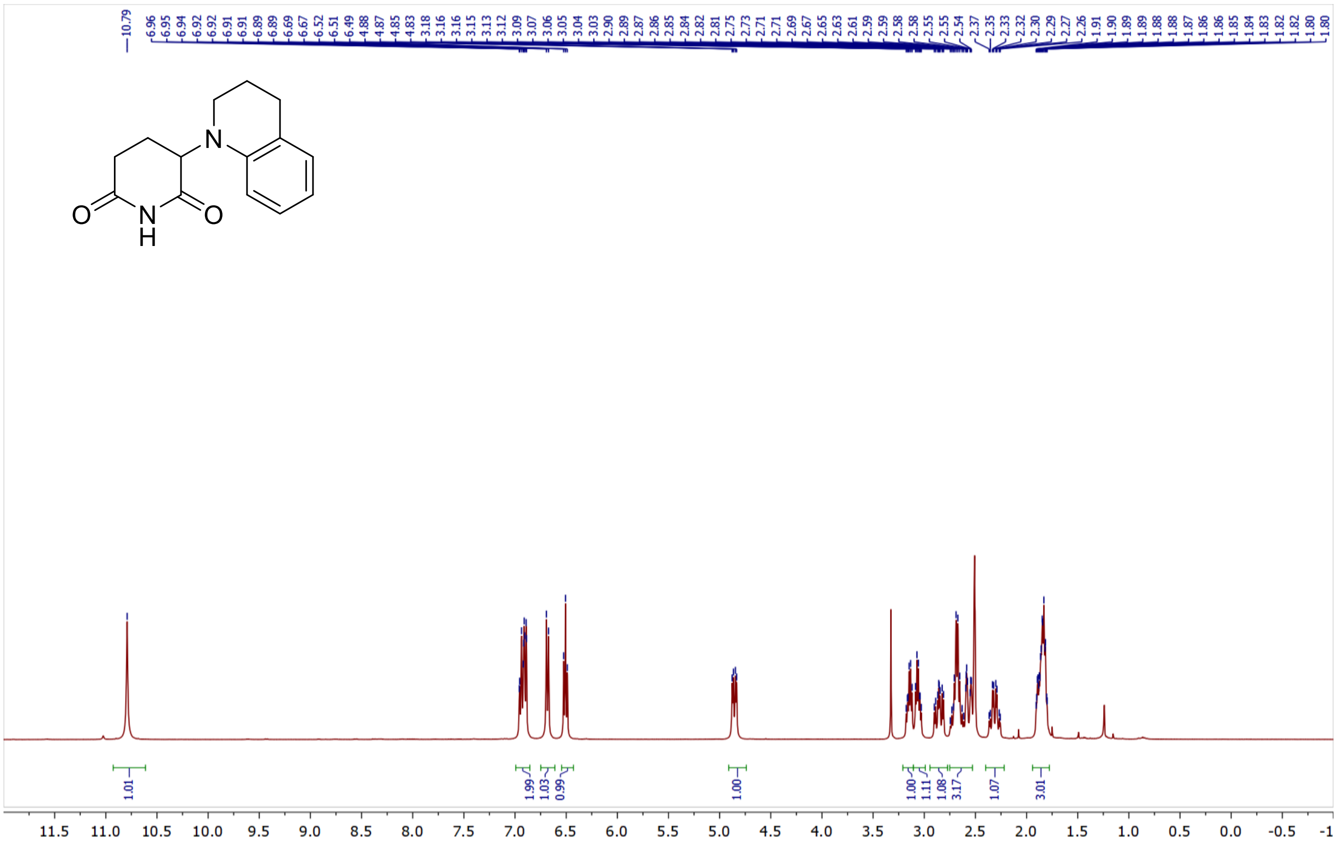

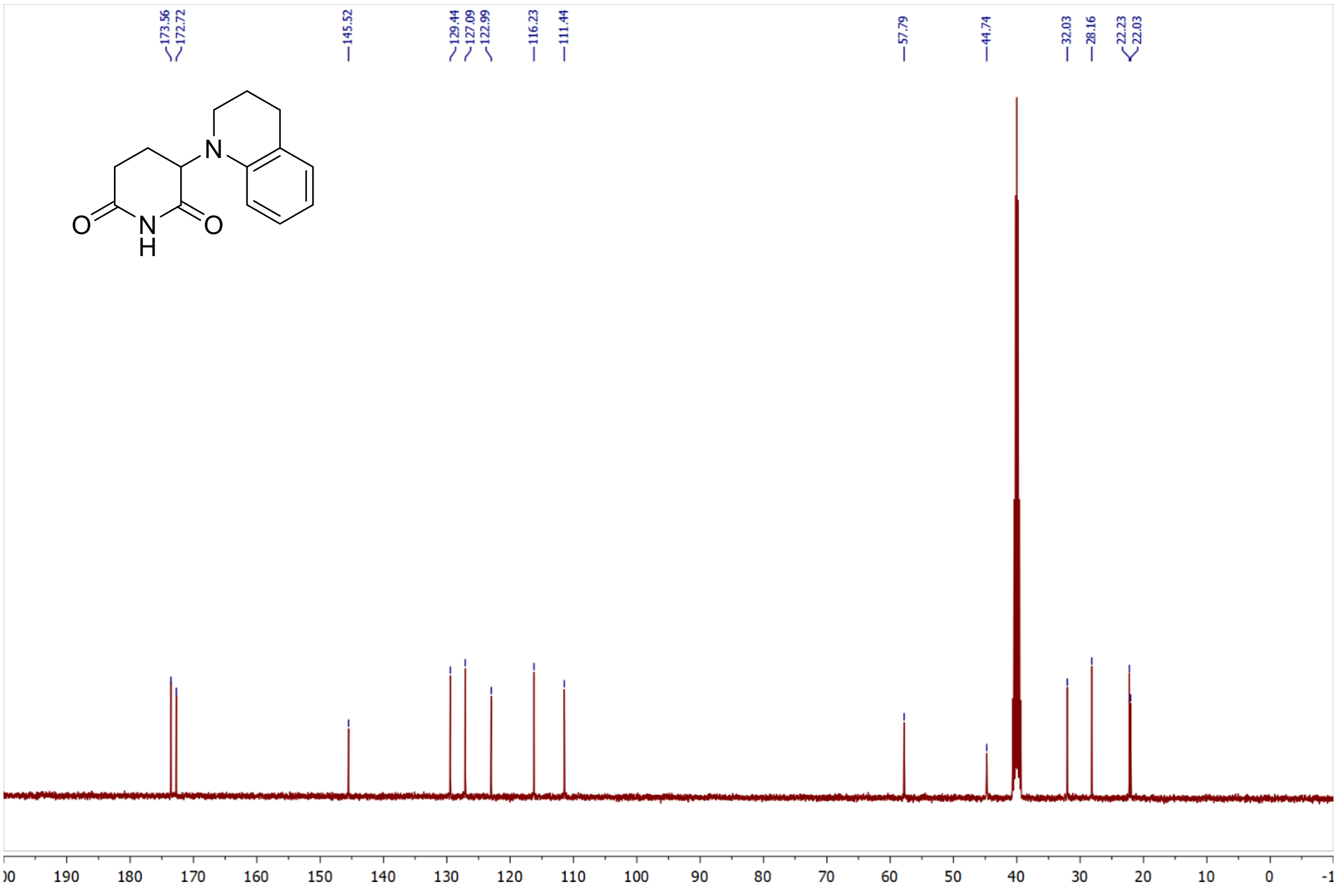

Copies of ^{1}H (400.13 MHz, $CDCl_3$) and $^{13}C\{^{1}H\}$ (101.61 MHz, $CDCl_3$) spectra of **10**

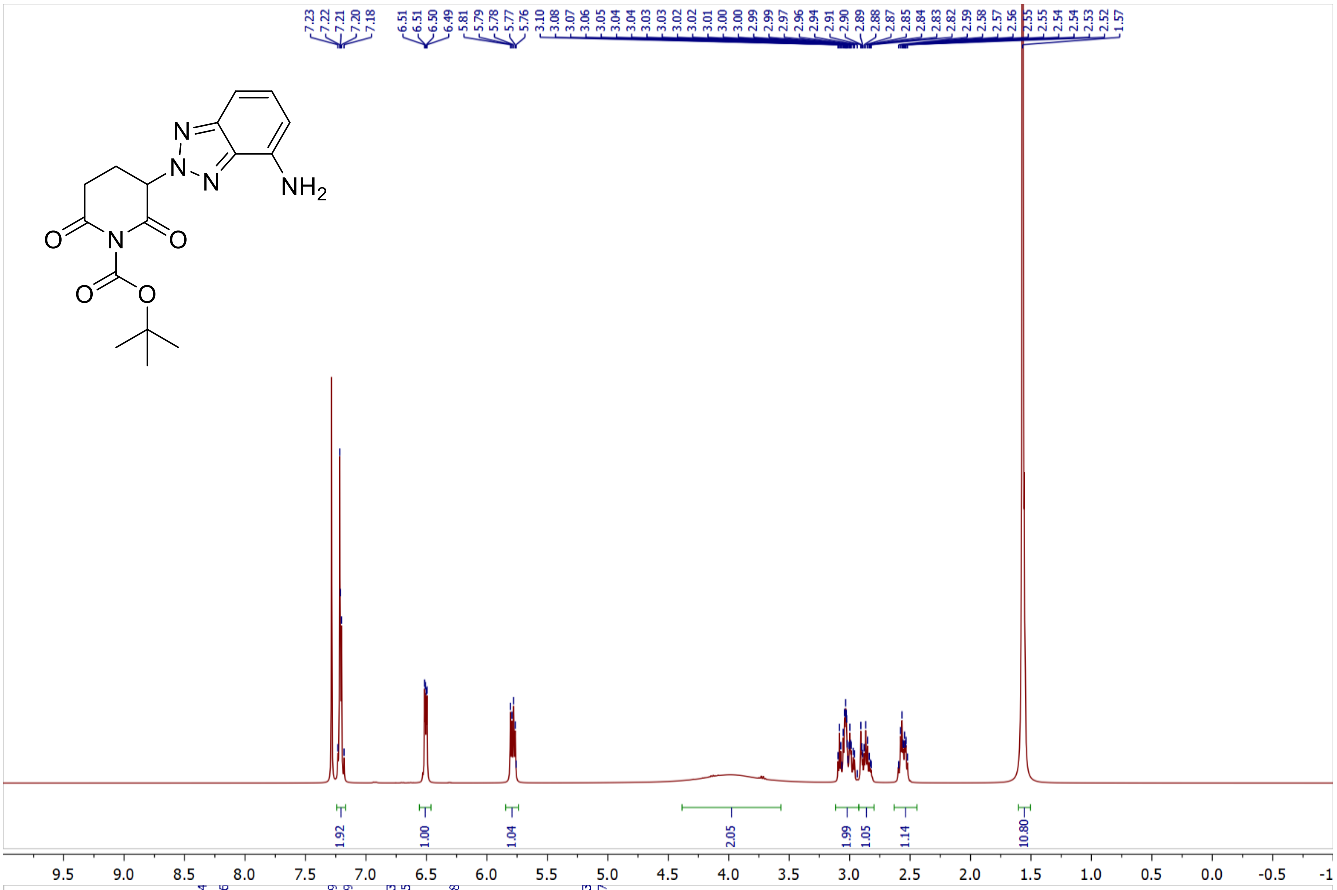

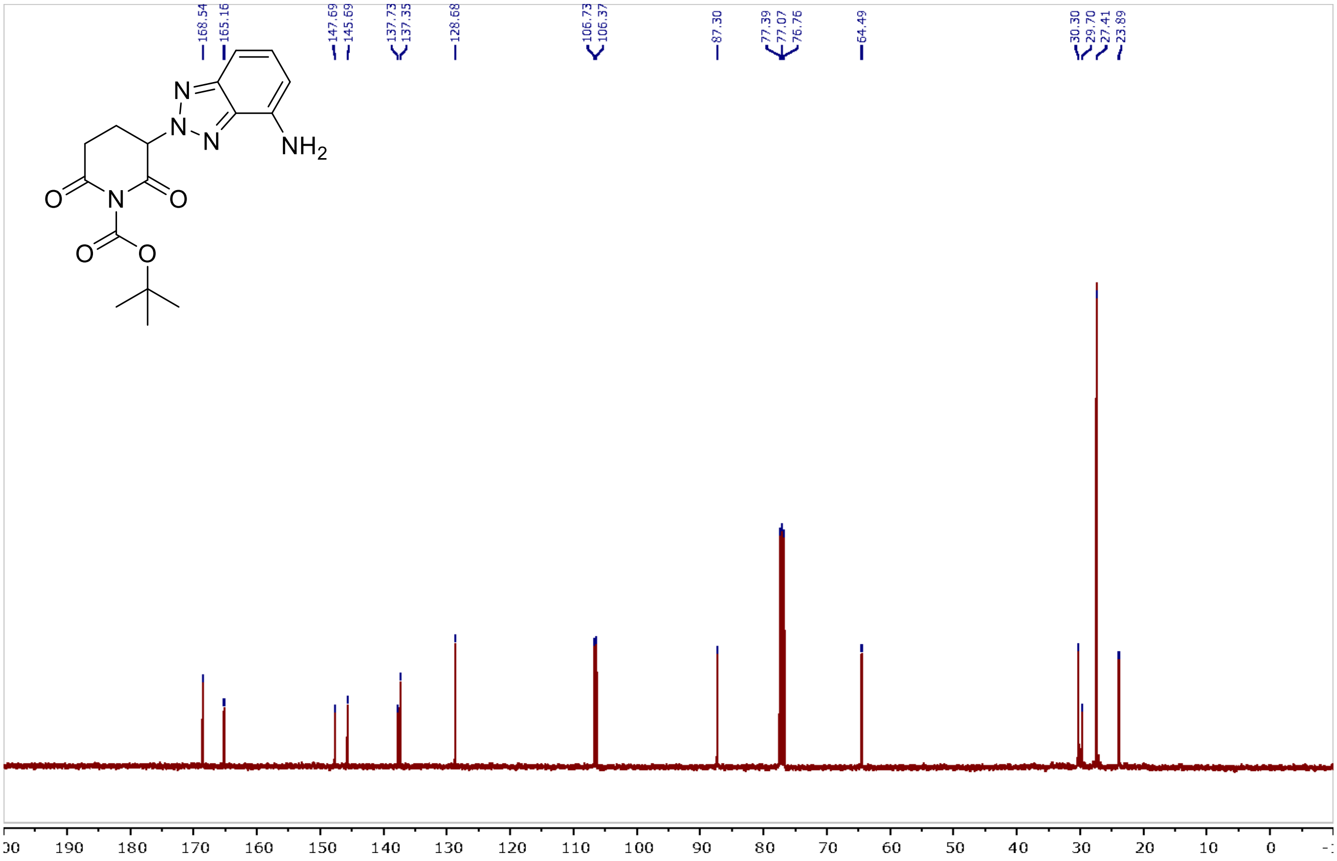

Supplement: File 1 — General experimental information, X-ray crystallographic data, synthetic procedures, analytical data and NMR spectra for the reported compounds. [file Beilstein_J_Org_Chem-19-1841-s001.pdf]
